# On the Origin of Substrate Specificity of Enzymes from the Amidohydrolase Superfamily

**DOI:** 10.1002/anie.202517873

**Published:** 2025-12-06

**Authors:** Lukas Drexler, Torben F. Fürtges, Till Rudack, Reinhard Sterner

**Affiliations:** ^1^ Institute of Biophysics and Physical Biochemistry Regensburg Center for Biochemistry University of Regensburg D‐93040 Regensburg Germany

**Keywords:** Amidohydrolase superfamily, Enantioselectivity, Enzyme catalysis, Enzyme promiscuity, Fleeting chiral intermediates

## Abstract

The sequencing of numerous genomes has led to the identification of open reading frames for millions of enzymes, many of which use unknown substrates. Hence, the identification of both primary and promiscuous activities remains a major challenge for enzyme research. Here, we identified the mechanistic basis of substrate specificity for members of the amidohydrolase superfamily (AHS). Comprehensive analyses of two AHS classes revealed that catalysis proceeds either via 1,4 or 1,6 nucleophilic conjugate addition mediated by a glutamine that is located at two different positions within the active site thereby shaping substrate scope in these enzymes. These different enzymatic properties result in an inverted enantioselectivity for fleeting chiral intermediates, which are transient chiral species on the reaction pathway from an achiral substrate to an achiral product. Moreover, we demonstrated that catalysis focuses on conserved core structures rather than on all moieties of a given substrate, which increases the degree of promiscuity and evolvability in these enzymes. Using site‐directed mutagenesis, we showed that an enzyme specialized in a specific nucleophilic conjugate addition can both readily adapt to secondary substrates and accommodate novel substrates by few amino acid exchanges. Hence, our study reveals mechanistic principles that underly substrate specificity, promiscuity, and enantioselectivity.

## Graphical abstract

Comprehensive analyses of two enzyme classes from the amidohydrolase superfamily (AHS) revealed that catalysis proceeds either via 1,4 or 1,6 nucleophilic conjugate addition and that this property defines substrate specificity. Moreover, although all substrates and products are entirely achiral, this mechanistic difference results in an inverted enantioselectivity for fleeting chiral intermediates in the two analyzed AHS classes.

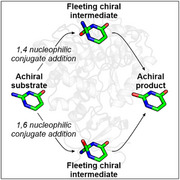

## Introduction

Enzyme catalysis is characterized by the enormous acceleration of chemical reactions, usually coupled with high specificity for the respective substrate. These exquisite properties are the consequence of millions of years of natural evolution resulting in optimized protein structures and highly fine‐tuned active sites.^[^ ^1^ ^]^ Interestingly, numerous studies in recent years have shown that many enzymes can also use substrates other than the ones for which they have been evolutionarily optimized, albeit with lower efficiency.^[^ ^2^, ^3^, ^4^, ^5^ ^]^ Such minor promiscuous side activities can often be improved significantly by only a few mutations and hence constitute promising starting points for the generation of novel enzymes in the context of natural evolution or enzyme engineering approaches.^[^ ^4^, ^5^, ^6^, ^7^ ^]^ However, although enzyme optimization by frequently used experimental techniques such as directed evolution are reliant on initial promiscuous activities, the mechanistic basis of enzyme promiscuity remains poorly understood. Moreover, even highly advanced automatic functional annotation algorithms are often unable to reliably predict the native substrate of an enzyme, let alone the identification of additional natural or even anthropogenic substrates.^[^ ^8^, ^9^, ^10^ ^]^ Therefore, identifying and rationalizing the scope of accessible substrates of a given enzyme and explaining or even predicting substrate specificities would advance our knowledge of enzyme catalysis and would also be important for the use of enzymes in industrial biocatalysis.^[^ ^11^, ^12^, ^13^ ^]^

Here, by comprehensive in silico, in vitro, and in vivo analyses we identified the structural determinants of substrate specificity in a class of enzymes whose reaction mechanism involves the formation of so‐called fleeting chiral intermediates (FCIs). FCIs are transient chiral species that are formed when a prochiral center of the substrate undergoes a reaction that temporarily breaks its symmetry, which is then regained in the product.^[^ ^14^, ^15^, ^16^, ^17^ ^]^ Hence, while the starting substrate and the final product can be entirely achiral,^[^ ^15^, ^17^ ^]^ a short‐lived stereocenter is formed within the reaction intermediate. Current questions about FCIs focus on whether these chiral intermediates appear in a homochiral form and whether they are merely a necessity of the chemical reaction, e.g. during the electrophilic or nucleophilic substitution at an sp^2^‐centered carbon.

**Figure 1:**
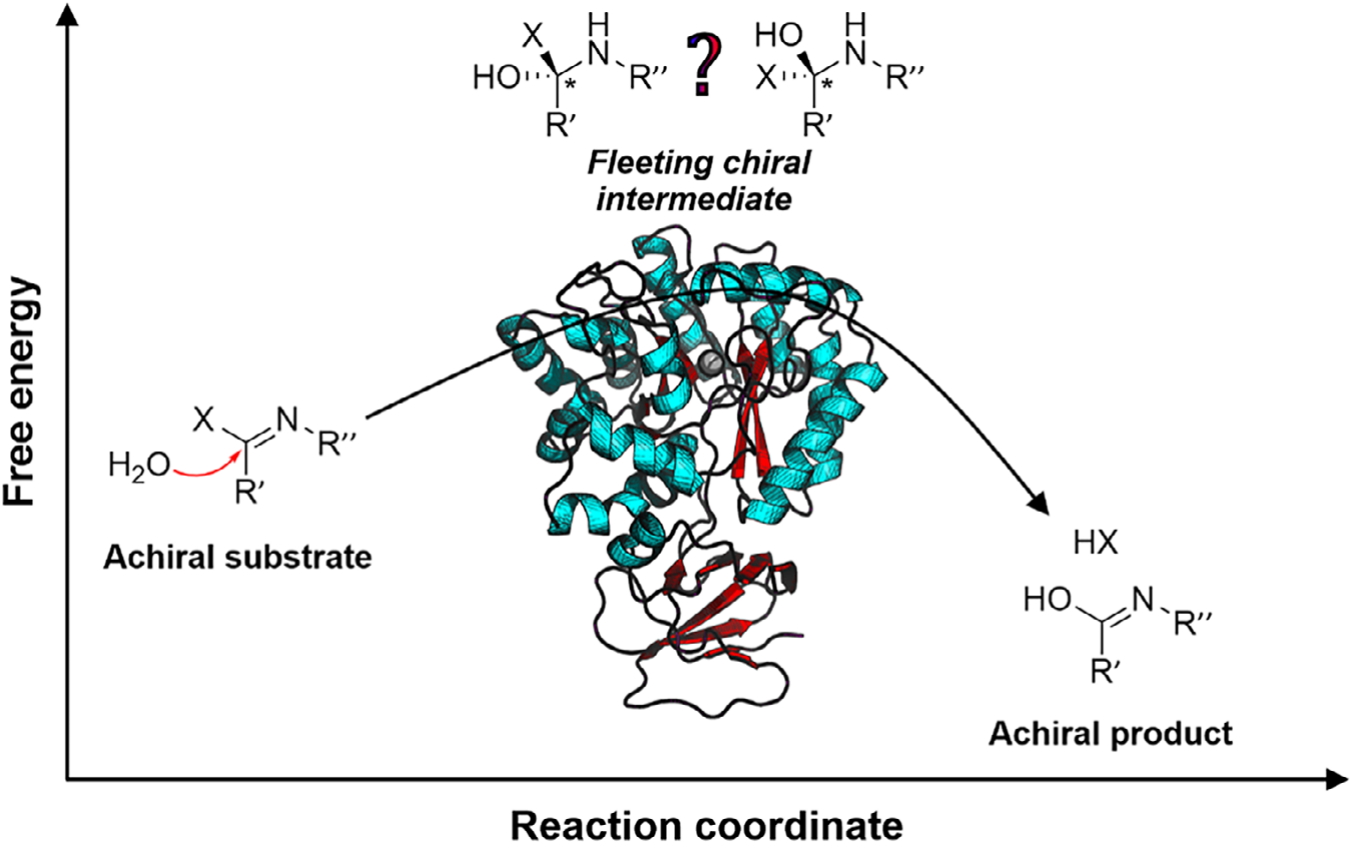
Fleeting chiral intermediates (FCIs) are formed during the reaction of AHS subtype III enzymes. Members of this subtype contain active site metal centers that assist the nucleophilic attack of a water molecule on an achiral substrate, targeting its C═N double bond.^[^ ^2^ ^]^ An energetically high FCI is formed, followed by the release of the leaving group and the concomitant formation of an achiral product.

Going beyond these aspects, we studied the occurrence and characteristics of FCIs in the context of substrate specificity and enzyme promiscuity. For this purpose, we used the example of the large amidohydrolase superfamily (AHS), which contains over one million proteins.^[^ ^2^ ^]^ Because of their versatile functions,^[^ ^2^, ^3^, ^4^, ^18^, ^19^, ^20^, ^21^, ^22^, ^23^, ^24^, ^25^, ^26^, ^27^, ^28^, ^29^, ^30^, ^31^, ^32^, ^33^, ^34^, ^35^, ^36^, ^37^, ^38^ ^]^ pronounced promiscuities,^[^ ^2^, ^3^, ^4^, ^35^, ^39^ ^]^ and the formation of FCIs,^[^ ^2^, ^33^, ^34^, ^35^, ^36^, ^37^, ^38^ ^]^ AHS enzymes are well suited to study enzymatic substrate specificity and its connection to FCIs. We focused on AHS enzymes from subtype III which are deaminases that share the frequently encountered (βα)_8_‐barrel protein fold and use similar metal‐dependent mechanisms that include highly conserved catalytic machineries.^[^ ^2^, ^34^, ^35^, ^39^ ^]^ In these enzymes, the reaction is initiated by the nucleophilic attack of a water molecule on an achiral substrate, targeting its C═N double bond. This leads to the formation of a short‐lived high‐energy FCI, while upon release of the leaving group an achiral product is formed (Figure 1).

To elucidate the determinants that allow an enzyme to bind a given substrate and to subsequently stabilize a particular FCI, we investigated the ability of various AHS III enzymes to hydrolyze a large collection of natural as well as xenobiotic substances (Figure S1, compounds **1‐24**). By analyzing specific features of the underlying enzymatic reaction mechanism, we identified structural determinants for the observed substrate specificity patterns of two different classes of AHS III enzymes: Dictated by the different location of a crucial active site glutamine residue, one class catalyzes the hydrolysis of substrates with a 4‐π‐electron system via 1,4 nucleophilic conjugate addition, while the other class catalyzes the hydrolysis of substrates with a 6‐π‐electron system via 1,6 nucleophilic conjugate addition. These mechanistic differences not only define substrate scopes of two subclasses within a huge and versatile enzyme superfamily but also result in an inverted enantioselectivity for FCIs, although all substrates and products are entirely achiral. Based on these findings, we could modify substrate promiscuity within the two classes by few mutations.

## Results and Discussion

To investigate enzymatic substrate specificity, we concentrated on AHS subtype III enzymes, which are deaminases that are distinguished from other subtypes by their active site metal centers.^[^ ^2^ ^]^ We generated a sequence similarity network (SSN) for 15 000 AHS subtype III sequences, including well characterized members such as guanine deaminases (GuaD), cytosine deaminases (CodA), 8‐oxoguanine deaminases (8‐OxoGuaD), adenine deaminases (ADE), adenosine deaminases (ADA), and the recently evolved xenobiotic‐degrading enzymes hydroxyatrazine ethylaminohydrolase (AtzB), N‐isopropylammelide isopropylaminohydrolase (AtzC), and ammelide aminohydrolase (TrzC).^[^ ^2^, ^24^, ^25^, ^26^, ^27^, ^28^, ^29^, ^30^, ^31^, ^32^, ^33^, ^34^, ^35^, ^36^ ^]^ Members of AHS subtype I such as ureases (URE) and phosphotriesterases (PTE) were also included and served as an external reference.^[^ ^2^ ^]^ At a sequence identity cutoff of 25.0 %, a distinct clustering of ADE/ADA, URE, PTE, and GuaD/CodA was observed (Figure S2A). At a sequence identity cutoff of 31.6 %, the GuaD and CodA clusters diverge (Figure S2B). While AtzC and TrzC are part of the CodA‐cluster at this threshold, 8‐OxoGuaD and AtzB are part of the GuaD‐cluster.

**Figure 2:**
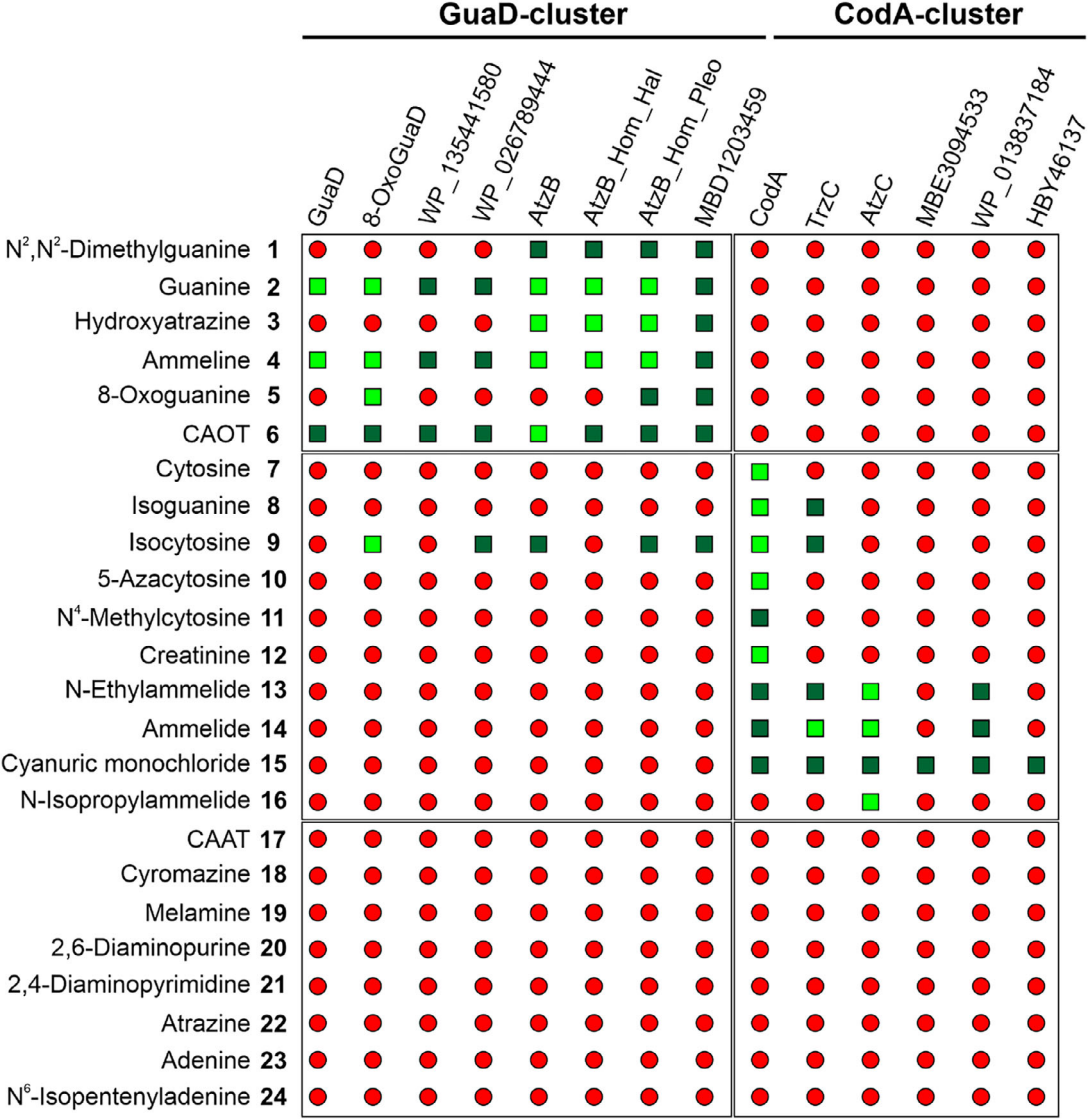
Substrate scopes of AHS III enzymes identified by HPLC‐based enzyme assays. 14 different AHS enzymes from the GuaD‐cluster and the CodA‐cluster were tested for the hydrolysis of 24 natural and xenobiotic substances including purines, pyrimidines, and *s*‐triazines. The underlying HPLC data are shown in **Source Data 1–24**, and the chemical formula for the catalyzed reactions are shown in Figure S4. Dark green squares indicate enzyme‐substrate pairs for which turnover was detected that has not been previously reported. Light green squares indicate enzyme‐substrate pairs for which turnover was detected, confirming data from the literature. Red circles indicate enzyme‐substrate pairs for which no turnover was detected.

We wondered whether the observed sequence divergence between the GuaD‐ and CodA‐clusters is reflected in different substrate scopes and is linked to distinct FCI configurations. To answer this question, we aimed to identify main and promiscuous activities of these two enzyme groups seeking potential overlaps in their substrate scope. For this purpose, we selected characterized and non‐characterized representatives from both clusters, such as GuaD,^[^ ^34^ ^]^ 8‐OxoGuaD,^[^ ^26^ ^]^ WP_135 441 580 (annotated as guanine deaminase), WP_02 678 9444 (annotated as guanine deaminase), AtzB,^[^ ^25^ ^]^ AtzB_Hom_Hal,^[^ ^4^ ^]^ AtzB_Hom_Pleo,^[^ ^4^ ^]^ and MBD1203459 (not annotated) from the GuaD cluster, and CodA,^[^ ^35^ ^]^ TrzC,^[^ ^31^ ^]^ AtzC,^[^ ^22^ ^]^ MBE3094533 (not annotated), WP_01 383 7184 (annotated as cytosine deaminase), and HBY46137 (annotated as cytosine deaminase) from the CodA cluster. We cloned the respective genes into appropriate vectors, expressed the genes in *Escherichia coli*, and purified the recombinant proteins to homogeneity (Figure S3). By using HPLC‐based enzymatic assays we tested the purified proteins for their ability to convert a library of compounds including both natural substances as well as xenobiotics. Beyond well‐known substrates for GuaD (**2**, **4**), 8‐OxoGuaD (**5**), AtzB (**3**, **6**), CodA (**7**, **8**, **9**, **10**, **12**), and AtzC (**13**, **14**, **16**), the screened compound library also included further purines, pyrimidines, and *s*‐triazines, that have not yet been linked to enzymatic hydrolysis. In doing so, we observed nine distinct hydrolysis reactions for enzymes of the GuaD‐cluster (Figure S4A), while for enzymes of the CodA‐cluster ten distinct hydrolysis reactions were observed (Figure S4B). Compounds **17**–**24** did not serve as substrates for any of the tested enzymes (Figure S4C). Figure 2 displays the identified substrate scopes of the 14 AHS enzymes, without differentiation between main and promiscuous activities.

Our comprehensive analysis unveiled the substrate scope of six previously uncharacterized AHS III enzymes: WP_135 441 580 and WP_02 678 9444 exhibit a substrate scope that resembles the one of GuaD, while the substrate scopes of MBD1203459 and WP_01 383 7184 resemble the one for AtzB and AtzC, respectively. MBE3094533 as well as HBY46137 exclusively convert **15**, which is hydrolyzed by all CodA‐cluster enzymes. Our measurements also revealed an extension of the substrate scopes for previously characterized enzymes: We could detect activities that have previously been associated with other enzymes (CodA + **13**/**14**; TrzC + **8**/**9**; AtzB_Hom_Pleo + **5/6/9**; AtzB + **9**; AtzB_Hom_Hal/8‐OxoGuaD/GuaD + **6**) as well as new activities for compounds that have not yet been linked to enzyme catalysis (**1**, **11**, **15**). Moreover, our findings confirm previously reported enzymatic activities as well as the absence of substrate turnover for most enzyme‐substrate combinations.

Particularly intriguing, the analyzed members of the GuaD‐ and CodA‐clusters showed distinct substrate scopes. Enzymes of the GuaD‐cluster were able to hydrolyze compounds **1‐6**, and **9**, whereas enzymes of the CodA‐cluster converted compounds **7–16**. These findings raised several crucial questions: Can this specific substrate distribution be explained mechanistically? Why is there only one overlap in the substrate scopes, and why is it specifically isocytosine **9**? What is the commonality between compounds **1–6**, and **9**, and compounds **7–16**, respectively? To answer these questions, we compared the structural constitution of all substrates (Figure S5).

Compounds converted by GuaD‐cluster enzymes (Figure S5A) and CodA‐cluster enzymes (Figure S5B) share a common conserved C‐N‐C‐O linkage (Figure S5 **, indicated in pink**). Apart from this molecular motif, the compounds are as diverse within one enzyme cluster as they are between clusters: Substrates include compounds as different as *s*‐triazines, purines, and pyrimidines. Moreover, the various leaving groups (Figure S5, depicted as the upper ring substituent) result in the formation of either NH_3_, NH_2_‐R (R = alkyl), R^1^NH‐R^2^ (R^1^, R^2 ^= alkyl), or Cl^−^. Hence, both enzyme clusters exhibit considerable diversity in ring size and leaving group of the substrates, which are thus highly unlikely to be decisive factors for the varying substrate scopes. Furthermore, all substrates of both the GuaD‐cluster and the CodA‐cluster possess an exocyclic oxygen as the second ring substituent (Figure S5, depicted as the right ring substituent), while the third ring substituent is highly variable, both within and between the two enzyme clusters (Figure S5, depicted as the left ring substituent). This indicates that the substituents are not critical for the differing substrate scopes either.

**Figure 3:**
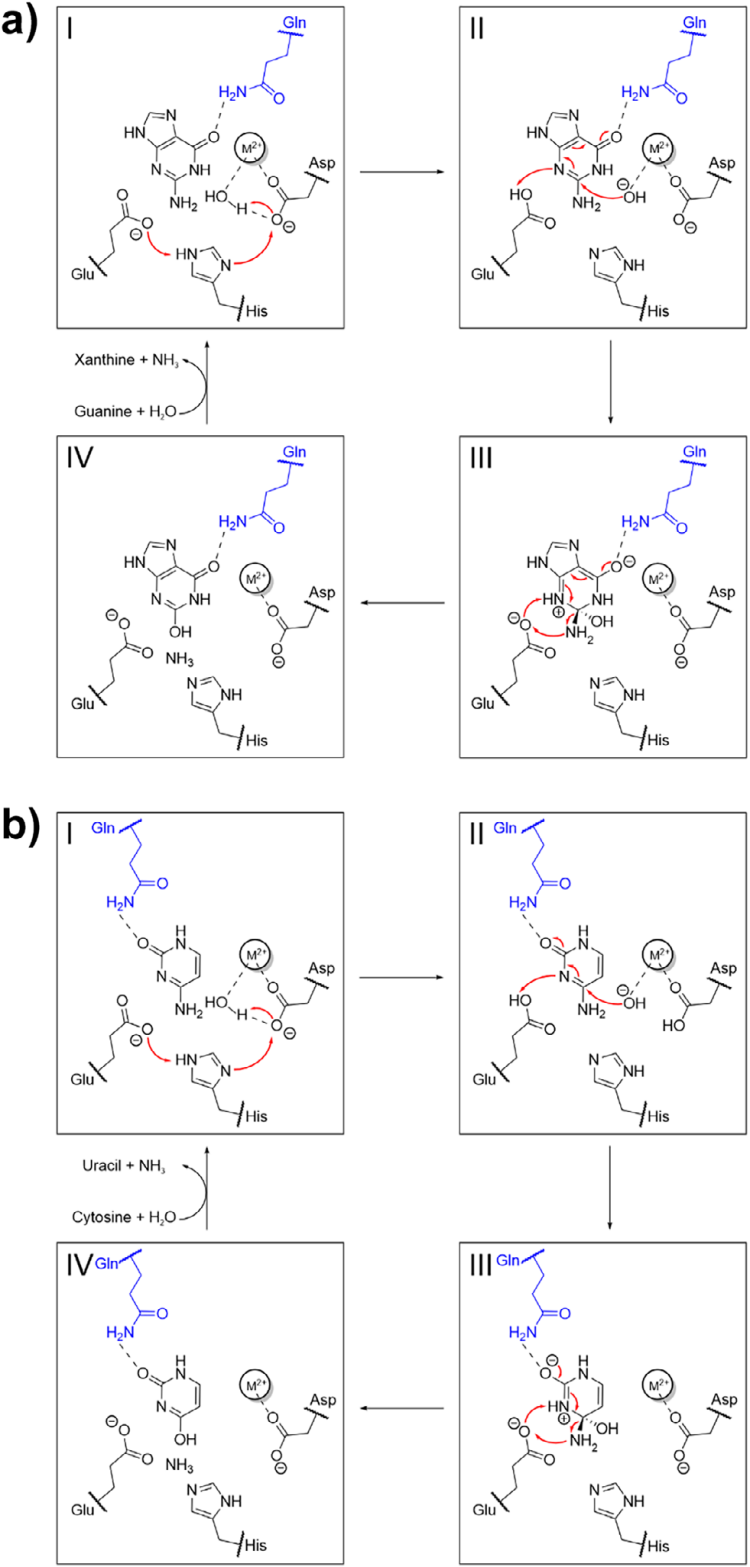
Reaction mechanisms of a) guanine deaminases and b) cytosine deaminases. Both reaction mechanisms proposed here can be divided into four steps: **I**: The water nucleophile is deprotonated by an aspartate and the proton is shuttled from the aspartate to a glutamate by a bridging histidine. **II**: The substrate is protonated at the lone pair localized in the sp^2^ orbital of N3 by the glutamate and the nucleophile attacks the C═N double bond of the substrate at the Bürgi–Dunitz angle (107°) through a (a) 1,6 conjugate addition or a (b) 1,4 conjugate addition. **III**: A new stereocenter is formed within the FCI. Shown is the zwitterionic resonance structure of the FCI in which the oxyanion is stabilized by a glutamine (in blue). The glutamate deprotonates N3 and protonates the amine substituent of the substrate resulting in a backflow of π‐electrons. **IV**: The leaving group is released, and the product xanthine/uracil is formed. While the catalytic machinery (Asp, His, Glu) is conserved in both enzymes, the main difference is the location of the FCI‐stabilizing glutamine. Electron flows and divalent metal ions are represented by red arrows and M^2+^, respectively.

**Figure 4:**
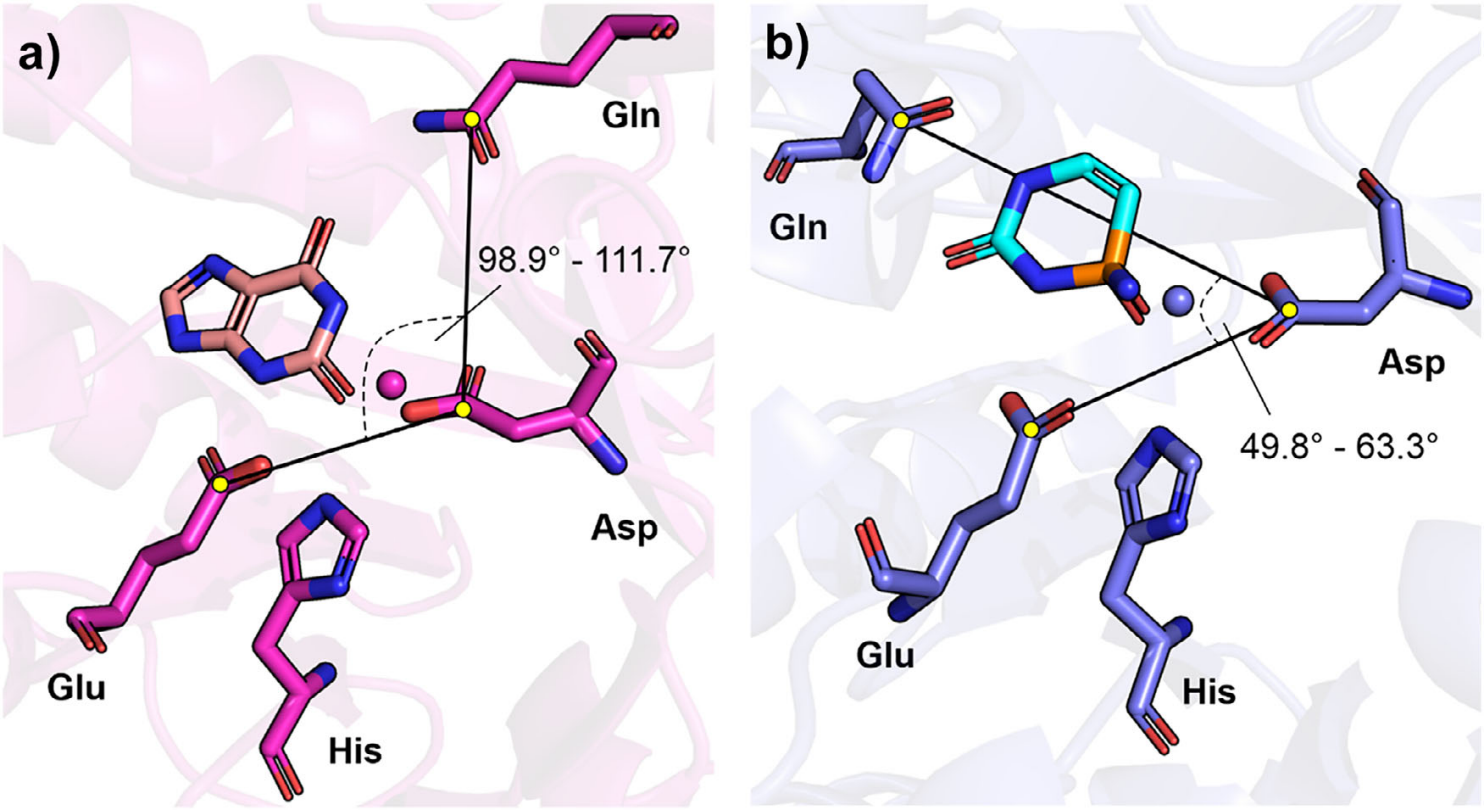
Active site of a) guanine deaminases and b) cytosine deaminases. a) Active site of *Escherichia coli* guanine deaminase (PDB: 6OHB) with the reaction product xanthine, inferred from an overlay with *Saccharomyces cerevisiae* guanine deaminase (6OHA).b) Active site of *Escherichia coli* cytosine deaminase (PDB: 3O7U) with bound FCI analog phosphonocytosine. Residues and divalent metal ions involved in catalysis (cf. Figure 3) are depicted as sticks and spheres, respectively. The angle between the substrate protonating (Glu), nucleophile activating (Asp), and FCI stabilizing (Gln) residues falls within a range of **a**) 98.8°–111.7° in GuaD‐cluster enzymes and **b**) 49.8°–63.3° in CodA‐cluster enzymes (cf. Figure S9).

For this reason, we turned our attention beyond the substrates to the catalyzed reactions and conducted a comparative analysis of the enzymatic mechanisms of GuaD and CodA, specifically focusing on how each enzyme binds the substrate and stabilizes the FCI. The type of reaction catalyzed by this class of enzymes corresponds to a nucleophilic substitution at an sp^2^‐centered carbon. Catalysis is initiated by activation of the water nucleophile via metal‐mediated deprotonation as well as activation of the electrophile by protonation:^[^ ^2^, ^34^, ^35^, ^36^ ^]^ In both GuaD (Figure 3a‐I) and CodA (Figure 3b‐I) water deprotonation is performed by an aspartate. The water‐abstracted proton is transferred from the aspartate to a glutamate by a bridging histidine residue. From the currently available literature it is uncertain whether this proton shuttle occurs before or after the substrate binds to the active site. In the next step, the glutamate transfers a proton to the substrate at the lone pair localized in the sp^2^ orbital of N3 (Figure 3a–II,3b‐II). These aspartate, histidine, and glutamate residues constitute the catalytic machinery, which is highly conserved among enzymes of both SSN clusters.^[^ ^2^, ^34^, ^35^, ^36^ ^]^ An active site glutamine hydrogen bonds the exocyclic oxygen of the substrate, thereby polarizing the carbonyl moiety and the π‐conjugated C═N double bond, which makes the latter more receptive to the nucleophile. The hydroxide anion can now attack the prochiral C‐atom of the C═N double bond in guanine **2** (Figure 3a‐II) or cytosine **7** (Figure 3b‐II) in a nucleophilic manner at the Bürgi–Dunitz angle (107°), which is accompanied by interference of the nucleophile‐HOMO with the electrophile‐LUMO. This results in a flow of π‐electrons within the attacked substrate toward the exocyclic oxygen and the formation of a new stereocenter.^[^ ^34^, ^35^, ^36^, ^40^, ^41^, ^42^, ^43^ ^]^ The chirality of the FCI (Figure 3a–III,3b‐III) is determined by the direction from which the nucleophile is approaching: An attack on the *Re*‐face of the prochiral ring yields one specific enantiomer, an attack on the *Si*‐face yields the other enantiomer. In GuaD and CodA, the conserved active site composition (Figure S6) unambiguously determines the direction from which the hydroxide anion approaches the substrate. Consequently, the absolute configuration of the resulting FCI is only dependent on the orientation of the substrate within the active site which in turn is dictated by the active site glutamine that coordinates the exocyclic oxygen of the substrate and stabilizes the oxyanion FCI (Figure 3a–III,3b‐III).^[^ ^34^, ^35^, ^36^ ^]^ Mutational analyses indicate the importance of these glutamine residues during catalysis as their replacement by asparagine and alanine yields inactive enzyme variants of GuaD and CodA.^[^ ^34^, ^35^ ^]^ Moreover, they differ considerably in their position in the active site and sequence: While the glutamine in GuaD succeeds β‐strand 1 of the (βα)_8_‐barrel core, the glutamine in CodA succeeds β‐strand 3 (Figure S6). In both cases, the reaction proceeds via deprotonation of N3 and protonation of the amine substituent by the glutamate. Backflow of the electrons (Figure 3a‐III and 3b‐III) finally expels the leaving group thereby producing xanthine and uracil, respectively (Figure 3a‐IV,3b‐IV). Both reaction mechanisms are well established and the positioning of the substrates as well as the chirality of the FCIs are further supported by several crystal structures (Figure 4, Figure S6, PDB: 6OHA, 4AQL, 1K70, 3O7U). Additionally, we corroborated the positioning of the respective substrate and FCI as shown in Figure 3‐I/III by computational modeling (Figures S7, S8).

Because of the crucial role of the glutamine residue in activating the electrophile and stabilizing the FCI, we wondered whether its differing positioning in GuaD and CodA relative to the catalytic triad Asp‐His‐Glu (Figure 3a‐III, 3b‐III, Figure S6) might explain the different substrate scopes of the two SSN clusters as shown in Figure 2. To investigate this, we first examined the active site composition of all experimentally analyzed enzymes (Figure S9). Proteins of the GuaD‐cluster have a glutamine in the same position as in GuaD (Figure 4a), whereas proteins of the CodA‐cluster have a glutamine in the same position as in CodA (Figure 4b).

**Figure 5:**
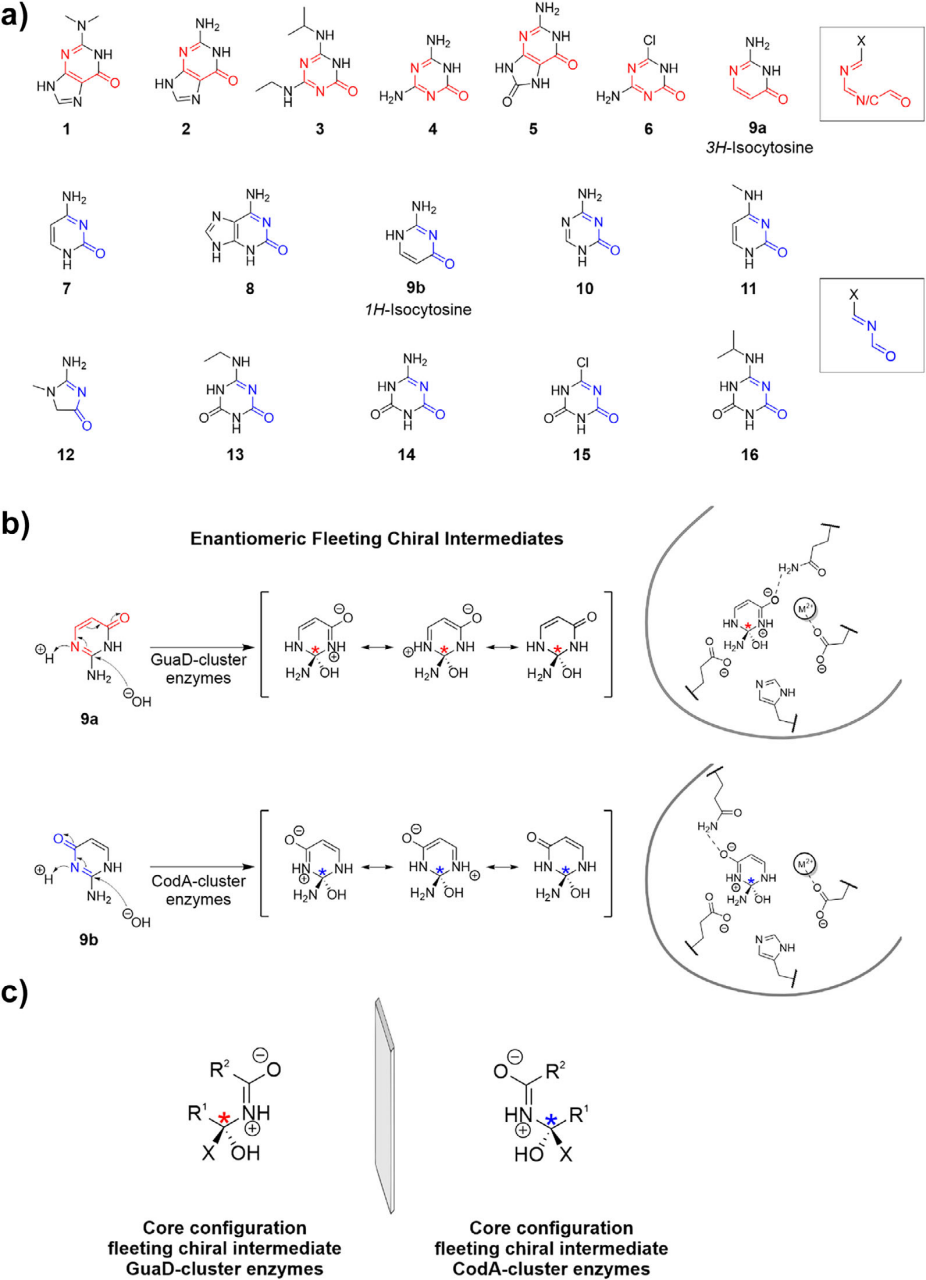
Substrates and FCIs of AHS III enzymes from the GuaD‐ and CodA‐clusters. a) Most reactive and most prevalent keto tautomeric forms of the identified substrates (cf. Figure 2, Figure S5). Substrates accepted by GuaD‐cluster enzymes exhibit a conjugated 6‐π‐electron system that links the prochiral electrophilic carbon and the exocyclic oxygen (indicated in red). Substrates accepted by CodA‐cluster enzymes exhibit a conjugated 4‐π‐electron system that links the prochiral electrophilic carbon and the exocyclic oxygen (indicated in blue). These moieties of the substrates participate in catalysis (cf. Figure 3) and are shown in a generalized form framed with a box. **9a** and **9b** have been shown to occur in a 1:1 ratio. **b**) FCI formation for the deamination of isocytosine as inferred from Figure S13. While in GuaD‐cluster enzymes substitution proceeds through 1,6 nucleophilic conjugate addition to **9a** at the Bürgi–Dunitz angle (107°), in CodA‐cluster enzymes substitution proceeds through 1,4 nucleophilic conjugate addition to **9b** at the Bürgi–Dunitz angle (107°). Shown are all plausible resonance structures of the FCI. The differing position of the essential glutamine in the two enzyme groups goes along with a 180°‐flipped orientation of the isocytosine ring in the respective active site. This results in the formation and stabilization of enantiomeric FCIs. Divalent metal ions are represented by M^2+^. **c**) By inferring the chirality of the FCIs for all observed reactions (Figure S14) distinct core configurations of the arising FCIs were identified that involve the conserved molecular motif as shown in Figure S5. The FCI core configurations of GuaD‐ and CodA‐cluster enzymes are mirror images, hence these enzymes show inverted enantioselectivities for FCIs.

To unveil the reason for this correlation, we analyzed the substrate requirements with respect to the enzymatic reaction mechanisms. For productive hydrolysis, the substrate has to be in a reactive tautomeric form. In this respect, the presence of a C═N double bond that can be activated by protonation and attacked by a nucleophile at the Bürgi–Dunitz angle (107°) is obligatory (Figure 3a–I/II, 3b‐I/II). This is particularly relevant for **7**, **8**, **11**, and **12** where only the tautomer as shown in Figure 5a can adopt this structural feature. Moreover, the nucleophilic attack is further facilitated by that tautomeric form of the substrate in which the exocyclic oxygen exists in its keto form: Such an electron‐withdrawing carbonyl decreases the electron density within the ring system and polarizes the C═N double bond, making it more receptive to the water molecule. Upon nucleophilic attack, the keto form also facilitates the immediate formation of the oxyanion, which is then stabilized by the active site glutamine (Figure 3a‐III, 3b‐III). Taking these aspects into account, the substrates are shown in their most reactive and most prevalent keto tautomeric form in Figure 5a.^[^ ^44^, ^45^, ^46^, ^47^, ^48^ ^]^ Notably, only isocytosine has two reactive tautomers (Figure 5a, **9a and 9b**), both of which allow for deamination and have been shown to occur in a 1:1 ratio.^[^ ^46^, ^49^, ^50^, ^51^ ^]^

Substrates accepted by the GuaD‐cluster (Figure 5a, compound 1–6 and 9a) and the CodA‐cluster (Figure 5a, 7–16) possess different π‐conjugated systems that link the prochiral electrophilic carbon and the exocyclic oxygen. Although in GuaD a movement of 6 π‐electrons is observed within the substrate (Figure 3a‐II), a movement of 4 π‐electrons is observed within the substrate of CodA (Figure 3b‐II) upon nucleophilic attack. Likewise, hydrolysis of reactive substrate tautomers proceeds through 1,6 nucleophilic conjugate addition to a 6‐π‐electron system (Figure 5a, **shown in red**) in GuaD‐cluster enzymes, while it proceeds through 1,4 nucleophilic conjugate addition to a 4‐π‐electron system (Figure 5a, **shown in blue**) in CodA‐cluster enzymes. Hence, our results suggest that enzymatic substrate specificity is influenced by different electron movement mechanisms and π‐system compositions of the reactive states of a given substrate. In this regard, isocytosine is unique as its two equally abundant tautomers^[^ ^46^, ^49^, ^50^, ^51^ ^]^ show a 6‐π‐electron system in the case of **9a** and a 4‐π‐electron system in the case of **9b**, which can be attacked by a nucleophile via 1,6 and 1,4 nucleophilic conjugate addition, respectively. This indicates why isocytosine is the only substrate that can be hydrolyzed by enzymes from both SSN clusters. Although for other substrates, such as guanine, further tautomers with differing π‐system compositions are conceivable (Figure S10), our results are consistent with previous observations that these constitute minor tautomeric forms.^[^ ^44^, ^45^, ^46^, ^47^, ^48^ ^]^ In addition, we predicted the orientation of guanine within CodA and of cytosine within GuaD in silico (Figure S11, Table S1). These analyses revealed a substrate positioning that is unproductive for catalysis either due to a 180° flipped orientation compared with productive binding as shown in Figure S7 and S8 or due to missing interactions with the active site glutamine.

These findings explain the correlation between the substrate scope and the location of the glutamine with respect to the catalytic machinery. The different π‐systems are chemically distinct entities requiring different enzymatic environments for productive catalysis. Specifically, the angle between the catalytic residues and the glutamine in GuaD‐cluster proteins falls within a range of 98.9°–111.7°, enabling productive hydrolysis of substrates through a 1,6 conjugate addition, while for CodA‐cluster proteins this angle lies between 49.8° and 63.3°, enabling productive hydrolysis of substrates through a 1,4 conjugate addition (Figure 4, Figure S9C). Hence, we have identified molecular patterns underlying substrate specificity within these enzymes: The presence of a 6‐π‐electron or a 4‐π‐electron system is a necessary criterion for the substrate to be acceptable for GuaD‐cluster or CodA‐cluster enzymes, respectively (Figure 5a). However, not all substrates fulfilling this criterion are used by all representatives of the respective cluster (Figure 2), indicating the influence of the individual active site environment for substrate acceptance.

Next, to investigate a possible connection between enzymatic substrate scope and FCIs in these AHS enzymes, we determined and compared the absolute configuration of the FCIs in both SSN clusters. To this end, we first focused on isocytosine as it is the sole substrate being hydrolyzed by enzymes from both clusters and thus provides a unique opportunity to compare the reaction mechanisms and the involved FCIs: The differing position of the essential glutamine (Figure 4) goes along with a 180°‐flipped orientation of the isocytosine ring in the respective active site (Figures S12, S13, Figure 5b) and allows GuaD‐cluster enzymes to catalyze a 1,6 nucleophilic conjugate addition to **9a**, while CodA‐cluster enzymes catalyze a 1,4 nucleophilic conjugate addition to **9b**. Hence, this results in a nucleophilic attack on different ring faces of the substrate in the two enzyme groups, since the catalytic machinery itself is conserved and unambiguously defines the direction of the water attack. As a consequence, the orientation of the substrate within the active site determines the chirality of the arising FCI: Enzymes of the GuaD‐cluster produce and stabilize the enantiomeric form of the FCI compared to enzymes of the CodA‐cluster (Figure S13, Figure 5b).

Based on these findings we inferred the chirality of the FCIs for all observed reactions (Figure S14). Interestingly, while for the nucleophilic attack taking place an appropriate π‐electronic composition of the substrates is essential (Figure 5a), the arising FCIs as depicted in Figure S14 show a distinct constitution. Whereas catalysis in GuaD‐cluster enzymes proceeding through 1,6 conjugate addition results in the formation of a specific FCI core configuration (Figure 5c **, left panel**), catalysis in CodA‐cluster enzymes proceeding through 1,4 conjugate addition results in the formation of the respective enantiomeric FCI core configuration (Figure 5c **, right panel**). Hence, similar to what was observed for isocytosine hydrolysis (Figure 5b) these FCI core configurations are mirror images (Figure 5c). The enzyme clusters thus show an inverted enantioselectivity for FCIs, even though all substrates and products are entirely achiral.

Hitherto, we have shown that catalysis in the analyzed amidohydrolases proceeds via 1,6 or 1,4 nucleophilic conjugate addition to distinct π‐electron systems and via stabilization of specific core configurations of the respective FCIs. We assume that these properties set the basis for enzymatic substrate specificity and hypothesize that these characteristics allow an enzyme to easily modify substrate promiscuity by few mutations. To address this point, we focused on the AtzB enzyme from the GuaD‐cluster and tested the effect of amino acid exchanges on the conversion of the substrates N^2^,N^2^‐dimethylguanine **1**, guanine **2**, and hydroxyatrazine **3**. Results listed in Table S2 show that the combinatorial replacement of only four active site residues allows the AtzB enzyme scaffold to rapidly adjust catalytic efficiencies thereby exploring a substantial portion of the specificity space for the three substrates (Figure S15). These findings indicate that enzymes can adapt to a specific substrate rapidly if the underlying core structure and π‐electron system of the primary substrate resembles that of the alternative substrate thereby allowing for a rapid specialist‐to‐specialist transition. Next, we tested whether mutations in a given enzyme can not only modify but also extend substrate promiscuity by enabling the acceptance of novel substrates with alternative R^1^ and R^2^ moieties while retaining the π‐electron system and thus the core configuration of the respective FCI as shown in Figure 5c. To address this point, we focused on the AtzC enzyme from the CodA‐cluster (Figures S16, S17) and determined the substrate scopes for five AtzC variants (Figure S18). Remarkably, the introduction of a maximum of three mutations into AtzC is sufficient to accept four novel substrates (**7**, **8**, **9**, **10**). Hence, our results reinforce that the ability of the examined AHS enzymes to catalyze the nucleophilic conjugate addition to a distinct π‐electron system and to stabilize specific core configurations of an FCI allows them to accommodate new substrates by few mutations. We substantiated this finding by steady‐state enzyme kinetics and growth experiments: Although wildtype AtzC did not show any activity toward cytosine **7** in vitro and in vivo, the acquisition of just two mutations enables the promiscuous hydrolysis of cytosine **7** in vitro (Figure S19) and growth of transformed *E. coli* on minimal media plates containing cytosine as sole nitrogen source, indicative of cytosine deamination in vivo (Figure S20). Moreover, the AtzC variants match the substrate specificity pattern observed for other CodA‐cluster enzymes (Figure 2) and thereby further indicate that the presence of a correct π‐electronic composition within the substrate is necessary for its hydrolytic degradation.

## Conclusion

In the current work, we deciphered the structural determinants of substrate specificity of two different classes of AHS enzymes by analyzing the π‐electronic composition of the productive state of a given substrate: GuaD‐ and CodA‐cluster enzymes catalyze either a 1,6 or 1,4 conjugate addition of a nucleophile to a distinct π‐electron system mediated by a glutamine that is located at two different positions close to the active site. This specific glutamine residue determines the orientation of the substrate within the active site and thus defines the absolute configuration of the arising FCI. Within each AHS enzyme, the FCI appears in a homochiral form, however, due to the differing position of the essential glutamine, GuaD‐ and CodA‐cluster enzymes generate enantiomeric forms of the FCI, meaning that they possess inverted enantioselectivities for FCIs. Equivalent glutamines are absent in other AHS clusters and uniquely appear in either the GuaD‐ or the CodA‐cluster. A putative common ancestor of both enzyme groups likely lacked an FCI‐stabilizing glutamine probably being capable of catalyzing both 1,6 and 1,4 nucleophilic conjugate additions. However, this would be accompanied by insufficient FCI stabilization and thus a much lower catalytic efficiency, as evidenced by previously reported glutamine mutants.^[^ ^34^, ^35^ ^]^ Plausibly, the diverging enzyme groups convergently invented a glutamine to stabilize the FCI, thus increasing their catalytic efficiency while losing the complete breadth of the substrate scope due to the accompanying specialization in a specific substrate group (with either a 6‐π‐electron or 4‐π‐electron composition). Hence, our results demonstrate that enantioselectivity can arise in a catalyst even when both, substrates and products, are entirely achiral. One should note that the observed FCI‐enantioselectivity is rather a consequence of evolution for activity than a selective advantage itself. A convincing demonstration of this well‐known phenomenon was recently published.^[^ ^52^ ^]^

Moreover, in the analyzed AHS members catalysis focuses on conserved core structures rather than on all moieties of a given substrate, which increases the degree of promiscuity and evolvability in these enzymes. This rationalizes the widespread occurrence of enzymatic promiscuity in general and the occurrence of unexpectedly high promiscuous activities for xenobiotic substrates with which an enzyme has probably never come into contact during its evolution (Figure S21). Moreover, we showed that an enzyme can adapt to secondary substrates rapidly if the underlying π‐system of the original substrate resembles that of the new substrate thereby allowing for a rapid specialist‐to‐specialist transition, which is substantiated by previous findings in our group for the evolution of the AtzB enzyme from an ancestor with guanine deaminase activity.^[^ ^4^ ^]^ Furthermore, an enzyme specialized in a specific nucleophilic conjugate addition can traverse the substrate scope by few mutations. This is reflected in the observation that single mutations within the AtzC enzyme can readily generate novel enzymatic activities, while the acquisition of no more than two amino acid exchanges can ensure cytosine deamination and thus bacterial growth. This prompted us to assume that regardless of a pre‐existing promiscuous activity for a specific substrate and based on the inherent potential to catalyze conjugate additions and to stabilize specific FCI core configurations, the mere existence of an enzyme scaffold can be evolutionarily advantageous under conditions that promote mutagenesis. Beyond natural protein evolution our findings have major implications for enzyme engineering approaches: An initial promiscuous activity for a particular substrate is not necessarily required, however, choice of an appropriate protein template that is already capable of stabilizing an FCI resembling that of the desired reaction might increase the chances of success.

## Supplementary Information

The authors have cited additional references within the Supporting Information.^[^ ^53^, ^54^, ^55^, ^56^, ^57^, ^58^, ^59^, ^60^, ^61^, ^62^, ^63^, ^64^, ^65^, ^66^, ^67^ ^]^

## Conflict of Interests

The authors declare no conflict of interest.

## Supporting information



Supporting Information

## Data Availability

The data that support the findings of this study are available from the corresponding author upon reasonable request.
