# Supplementary material for: On the Origin of Substrate Specificity of Enzymes from the Amidohydrolase Superfamily
Source: Angew Chem Int Ed Engl. 2025 Dec 6;65(4):e17873. doi: 10.1002/anie.202517873 (PMC12828455; doi:10.1002/anie.202517873)
Supplement: Supplementary file 1 — Supporting Information [file ANIE-65-e17873-s001.pdf]

Supporting Information

On the Origin of Substrate Specificity of Enzymes from the Amidohydrolase Superfamily

*Lukas Drexler, Torben F. Fürtges, Till Rudack, Reinhard Sterner**

Institute of Biophysics and Physical Biochemistry, Regensburg Center for Biochemistry, University of Regensburg, D-93040 Regensburg, Germany.

***Corresponding Author:**
Reinhard Sterner: Email: reinhard.sterner@ur.de

ORCID

Lukas Drexler: orcid.org/0000-0001-8193-3256

Till Rudack: orcid.org/0000-0003-2693-9561

Reinhard Sterner: orcid.org/0000-0001-8177-8460

Table of Contents

1. Experimental Section – Material and Methods (p. 3 - 7)
2. Supplementary Figures and Tables (p. 8 - 39)
3. Source Data – HPLC Chromatograms (p. 40 - 92)
4. References (p. 93)

Experimental Section - Material and Methods

Bacterial Strains and Chemicals

BL21 (DE3) Gold (Agilent Technologies), NEB Turbo (New England Biolabs), and BW25113 Δ*codA* (JW0328, CGSC Yale) *Escherichia coli* strains were used for protein production, plasmid amplification, and growth experiments, respectively. Chemicals were purchased from commercial sources and were of analytical grade or higher. N-ethylammelide and N-isopropylammelide were synthesized as described previously.[1]

Sequence Similarity Networks (SSN)

To generate sequence similarity networks, homologous sequences of previously characterized AHS enzymes were retrieved by BLAST searches using standard parameters (500 hits for adenine deaminase, phosphotriesterase, urease, adenosine deaminase; 1000 hits for AtzB, AtzC, TrzC, 8-oxoguanine deaminase; 5000 hits for guanine deaminase and cytosine deaminase).[2] Then, the sequences were submitted to the EFI-EST webserver and SSNs were created to cluster closely related sequences using standard parameters.[3] SSNs with a 60% representative node identity (RepNode60) were visualized in Cytoscape (3.10.1) at a sequence identity threshold of 25.0% and 31.6%.

Multiple Sequence Alignment (MSA) and Sequence Logos

For mutational planning of AtzC variants the 500 closest homologues of AtzC were identified by BLAST and used to generate a multiple sequence alignment with MAFFT.[2,4] Based on this MSA, sequence logos for the positions corresponding to K65, W251, and W309 in AtzC were created by means of WebLogo.[5]

Analysis of Protein Structures

While for GuaD (PDB: 6OHB, 6OHC), CodA (PDB: 1K70, 3O7U), and AtzC (PDB: 4CQB, 2QT3) crystal structures are available, structures for all other proteins were retrieved from the AlphaFold2 database.[6] Substrate positioning within the active sites was predicted using Chai-1.[7] Visualization of protein structures, calculation of RMSD values, and determination of angles between catalytic residues was conducted using PyMol.

Molecular Modeling

To compare the detailed active site architecture of cytosine deaminase (CodA) and guanine deaminase (GuaD) in complex with their respective native substrates, we constructed structural models of the pre-reaction state I (cf. Figure 3-I) and the FCI-state III (cf. Figure 3-

III) of both deaminases from *E. coli*. First, the active sites were modeled based on related experimentally resolved X-ray crystallography structures (as detailed in the following paragraph) followed by an iterative cycle of energy optimization and molecular mechanics (MM) simulation to relax the substrate with respect to the protein environment.

Cytosine Deaminase Modeling

As starting point for the modeling of cytosine deaminase in complex with cytosine we used the X-ray crystallography structure of *E. coli* cytosine deaminase with bound inhibitor 4-hydroxy-3,4-dihydro-1*H*-pyrimidine-2-one (PDB: 1K70) that mimics the FCI-state III. Based on the atom positions of this structure, the cytosine FCI-state III was built by conversion of the inhibitor into cytosine by substituting the hydrogen at the chiral center with an amine group and subsequent energy optimization of the ligand utilizing the AMBER all-atom force field (Zitat 10). Then, the active site was relaxed as detailed below. We modeled the pre-reaction state I using the relaxed FCI-state III as starting point and converted the hydroxyl group into a water molecule followed by planarizing of the former sp^3-hybridized chiral reaction center. Finally, the resulting active site structure was relaxed.

Guanine Deaminase Modeling

As starting point for the modeling of guanine deaminase in complex with guanine we used the X-ray crystallography structure of the apo *E. coli* guanine deaminase (PDB: 6OHC). Based on the position of the zinc ion coordinated water, the FCI-state III was constructed by docking of the FCI into the active site using MAXIMOBY/MOBY.[8] State I was model in an analogous manner as described for cytosine deaminase by conversion of the substrate and subsequent relaxation of the active site.

Active Site Relaxation

To relax the active sites of each of the above-described constructed models including the water molecules resolved in the corresponding crystals structures, we used the following workflow. We first protonated the structures based on local pK_a value calculations of each residue using the algorithm implemented in MAXIMOBY that is based on the approach of Nielsen and Vriend.[9] The active center was relaxed using MAXIMOBY/MOBY to perform an energy optimization followed by a short MM simulation and a second step of energy optimization. For the energy optimization the conjugate gradient optimizer was used. To relax the structures a 100 steps simulation with 1 fs step size at 300 K of all residues within 4 Å of the respective substrate was performed using the generalized born model with augmented hydrophobic

solvent accessible surface area (GB/SA)[10] continuum water model. For both, optimization and MM simulation, the AMBER all-atom force field[11] was used and only the atoms within 4 Å distance to the substrate were movable. The rest of the protein was kept fixed.

Force Field Parametrization of Substrates

Parameters of the substrates of guanine and cytosine deaminases were generated based on the AMBER all-atom force field. Based on the chemical properties of each center, a corresponding general atom type of the force field was assigned. All bonding and non-bonding parameters were derived from their predefined center types. Charges of the newly defined residues were determined from quantum chemical calculations using the PBE functional and the 6-31G* basis set.

Computational Substrate Interaction Analysis

To gain insights into the substrate scopes of the GuaD- and CodA-clusters, the interactions of the identified substrates were analyzed in complex with *E. coli* GuaD and CodA as representatives of each cluster. Therefore, all substrates were superimposed with the native substrate in the relaxed pre-reaction state I models of both deaminases. Specifically, for this superimposition the alignment of the leaving group, the prochiral carbon, and the unprotonated nitrogen (*e.g.* N3 in guanine and cytosine) of the π-electron system were considered, resulting in a C=N double bond correctly oriented for nucleophilic conjugate addition. For the interaction analysis we used the contact matrix algorithm in MAXIMOBY and the PyContact plugin[12] for VMD 1.9.4.[13] If an interaction between the exocyclic oxygen of the 6/4-π-electron system and the respective active site glutamine was observed, we considered the complex as catalytically active. If no such interaction was observed, we considered the complex as inactive.

Gene Cloning and Site-Directed Mutagenesis

Genes coding for the 14 wildtype proteins were codon-optimized for recombinant expression in *E. coli* and purchased from GeneArt (Thermo Fisher Scientific) as gene strings. Flanking *Bsa*I restriction sites enabled cloning of the genes into pUR22_*Bsa*I and pUR23_*Bsa*I (expression vectors) as well as pTNA_*Bsa*I (vector for growth assays) using a coupled digestion/ligation reaction with *Bsa*I and T4-DNA ligase.[14] AtzC variants were created by site-directed mutagenesis employing a modified version of the QuikChange protocol. Primers harboring specific mismatches were designed to amplify the entire plasmid. The resulting linear amplicon was treated with T4 polynucleotide kinase and T4 DNA ligase (5 U; 10 U; 30 min 37 °C; 30 min 25 °C) in 1×T4 ligase buffer in a total volume of 50 µl. The resulting ligation

solution containing circular, mutated plasmids, was used for the transformation of *E. coli* cells (NEB Turbo) without further purification. After plasmid isolation, the integrity of the construct and the presence of the desired mutation was confirmed in each case by Sanger sequencing (Microsynth Seqlab).

Gene Expression and Protein Purification

All proteins analyzed in this study were produced as either N-terminal or C-terminal His_6-tagged fusion constructs. For gene expression, the *E. coli* strain BL21 (DE3) Gold (Agilent Technologies) was transformed with the respective expression plasmid coding for the protein of interest. Cells were grown in LB medium (37 °C, 140 rpm) supplemented with 150 mg/ml ampicillin to an OD_{600} of 0.6 followed by induction of gene expression by the addition of 0.5 mM IPTG. Cells were cultivated overnight (20 °C, 16 h), harvested by centrifugation (4000 g, 20 min, 4 °C), and resuspended in 100 mM Tris/HCl (pH 7.5), 300 mM KCl, 10 mM imidazole. Afterwards, cells were disrupted by sonication (Branson Sonifier W-250D, 60 % amplitude, 3 min, 2 s pulse, 2 s pause) and cell debris and insoluble aggregates were removed by centrifugation (16000 g, 45 min, 4 °C). The proteins were purified from the supernatant by immobilized metal affinity chromatography (IMAC) using an ÄKTA-purifier system with a HisTrap excel column (GE Healthcare) applying a linear imidazole gradient (10 mM – 1 M over 15 CV). The proteins were further purified by preparative size exclusion chromatography (SEC) using an ÄKTA-purifier system with a HiLoad 26/60 Superdex 75 or a HiLoad 26/60 Superdex 200 column (GE Healthcare) equilibrated with 50 mM Tris/HCl (pH 7.5), 50 mM KCl. Protein elution was continuously monitored at 280 nm and fractions containing the protein of interest were identified by SDS-PAGE and pooled. Protein concentrations were determined by absorption spectroscopy at 280 nm (Thermo Fisher Scientific, NanoDrop One) using a molar extinction coefficient that was calculated from the amino acid sequence (**Table S3**).[15] The purified proteins were frozen in liquid nitrogen and stored at -70°C.

HPLC-based Substrate Screening and Determination of Substrate Scope

To confirm the activity of an enzyme toward a certain substrate and to identify product composition, qualitative enzyme assays based on HPLC analysis were conducted. All enzymatic assays contained 500 µM of the respective substrate, 50 mM potassium phosphate (pH 7.5), and 2 µM of the tested enzyme in a total volume of 150 µl. As a reference, genuine substance standards were set up in the same way without any enzyme present. Following incubation at 25 °C and 500 rpm for 24 h, all reactions were stopped by centrifugation using a filter tube with a pore size of 10 kDa to remove the enzymes. The reaction products were subsequently analyzed by reversed-phase HPLC using an Agilent system (1100 series) with

an Eclipse XDB-C18 (4.6x150) column. The separation was performed at 20 °C using 0.1% formic acid in water as buffer A and 0.1% formic acid in acetonitrile as buffer B with an isocratic elution (5 % buffer B, 1 ml/min, 0 - 7.5 min) followed by a gradient elution (5 - 100 % buffer B, 1 ml/min, 7.5 - 20 min). The hydrolysis products of compounds **13**, **14**, **15**, and **16** were additionally analyzed using a Luna Omega 5 µm Polar C18 100Å (150x3.0) column with isocratic elution (100 % buffer A, 1 ml/min, 0 - 10 min) for direct detection of compound **32**.

Steady-State Enzyme Kinetics

Catalytic parameters for the hydrolysis of N^2,N^2-dimethylguanine **1,** cytosine **7**, and ammelide **14** were determined under steady-state conditions by a direct photometric assay. The difference in absorbance between **1** - **27** ($\Delta\varepsilon_{294}$ = 3187 $M^{-1}cm^{-1}$), **7** - **29** ($\Delta\varepsilon_{286}$ = 680 $M^{-1}cm^{-1}$), and **14** - **32** ($\Delta\varepsilon_{230}$ = 7030 $M^{-1}cm^{-1}$) was used to monitor the respective reaction on a spectrophotometer (JASCO V-750). Reactions were performed in triplicates at 25 °C. Standard assays contained either 10−6000 µM N^2,N^2-dimethylguanine **1**, or 500−4000 µM cytosine **7**, or 30−2000 µM ammelide **14** in a total volume of 300 µL of potassium phosphate buffer (50 mM, pH 7.5). When a constant baseline absorbance was reached, reactions were initiated by the addition of either an AtzB variant, or CodA, or AtzC_D188H_H219E. In all cases, it was assured that the enzyme concentration was at least 10 times lower than the substrate concentration. Initial velocities (v_i) were calculated from the initial linear part of the resulting curve via division by the respective differential molar extinction coefficient. The determined reaction velocities were then normalized to the applied enzyme concentration (v_i/E_0) and plotted against the substrate concentration. By fitting the data to the Michaelis-Menten equation in Origin 2022 (© OriginLab Corporation), the Michaelis constant K_M and the turnover number k_{cat} were determined.

Growth assays

To assess cytosine deaminase activity *in vivo*, bacterial growth on M9 minimal medium containing cytosine as sole nitrogen source was investigated. To this end, *E. coli* strains that either harbor a gene for cytosine deaminase within their genome (BL21 (DE3) Gold) or a deletion of this gene (BW25113 Δ*codA*) were transformed with different pTNA-based constructs of *codA*, *atzC*, and *atzC_D188H_H219E*. The transformed cell suspensions were then plated on M9 plates supplemented with 150 mg/ml ampicillin, while lacking NH_4Cl and instead containing cytosine (0.5 mg/ml). The plates were then incubated at 37 °C. The pTNA vector ensures low constitutive expression of the gene of interest by a tryptophanase promoter.[14] Deamination of cytosine *in vivo* would release ammonia that can be used by the cells as a nitrogen source, thereby enabling bacterial growth.

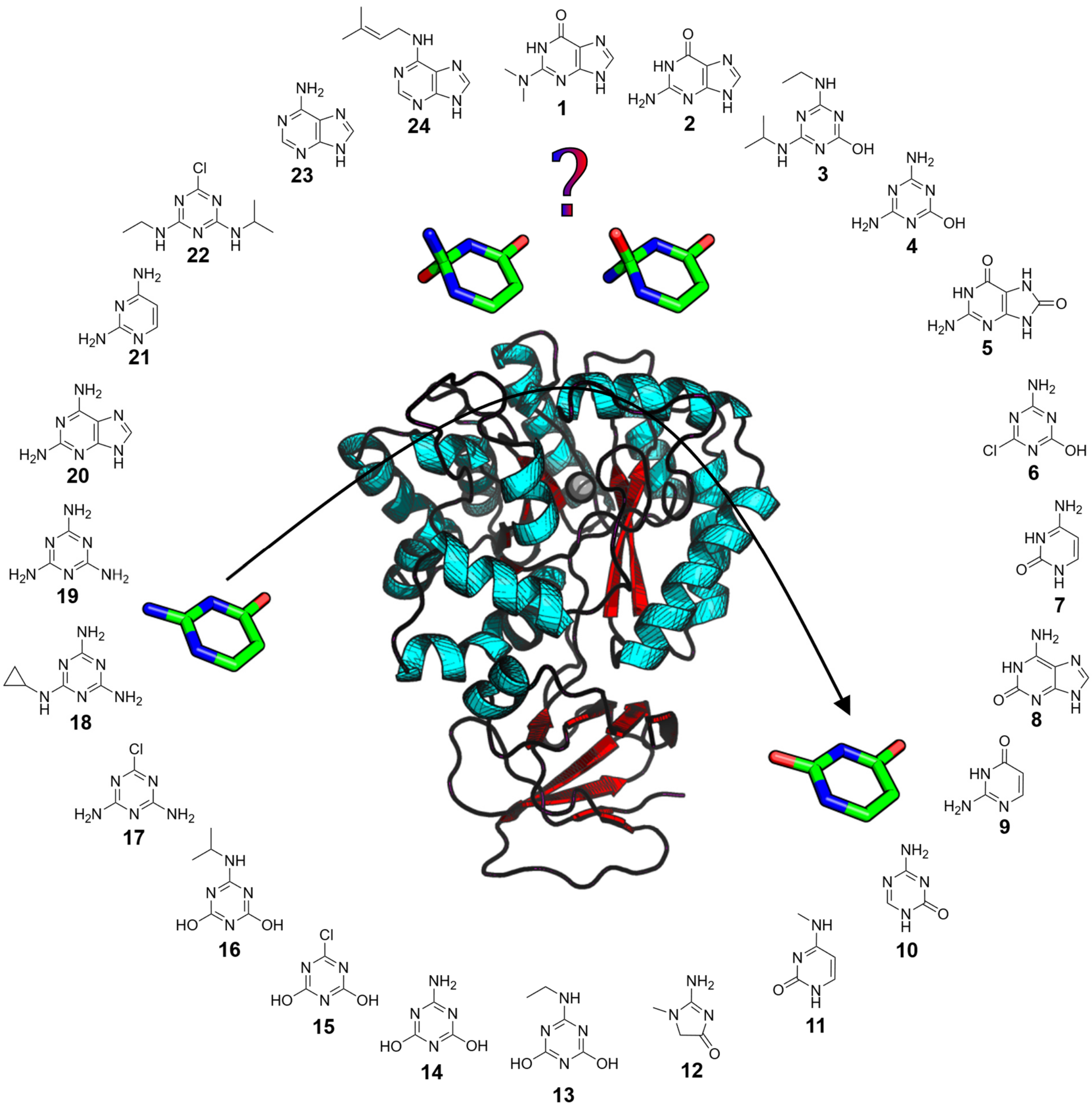

Figure S1: Connection between fleeting chiral intermediates and enzymatic substrate scope in the amidohydrolase superfamily. To investigate the link between FCIs and enzymatic substrate scope, we examined a diverse set of natural and xenobiotic compounds (**1-24**) as potential substrates for AHS enzymes. Then, the absolute configuration of the FCIs generated during the reactions was determined. Here, exemplarily, the two possible tetrahedral fleeting chiral intermediates of isocytosine deamination to uracil are shown.

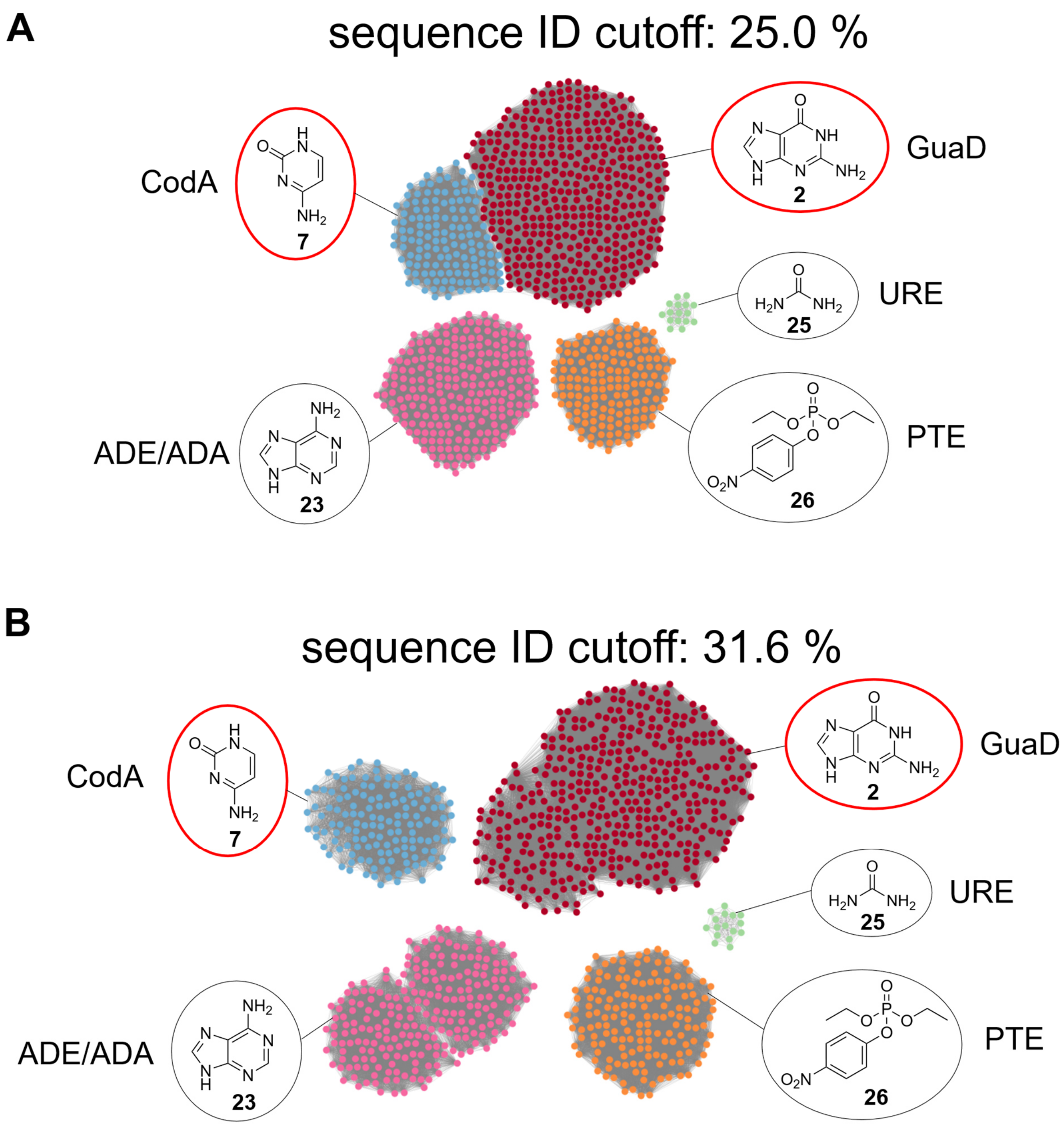

Figure S2: Sequence similarity networks (SSNs) of amidohydrolase superfamily (AHS) enzymes. Shown are enzymes that have been experimentally characterized in previous studies[16–20] and that catalyze the nucleophilic attack on C=O double bonds (e.g. ureases hydrolyzing urea **25**), P=O double bonds (e.g. phosphotriesterases hydrolyzing paraoxon **26**), and C=N double bonds (e.g. deaminases hydrolyzing guanine **2**, cytosine **7**, or adenine **23**). The latter ones are representatives of AHS subtype III which are distinguished from other subtypes by their active site metal centers.[21] (**A**) SSN at a sequence identity (ID) cutoff of 25.0 %. (**B**) SSN at a sequence identity (ID) cutoff of 31.6 %. At this threshold, the cytosine deaminase cluster (blue) separates from the guanine deaminase cluster (red).

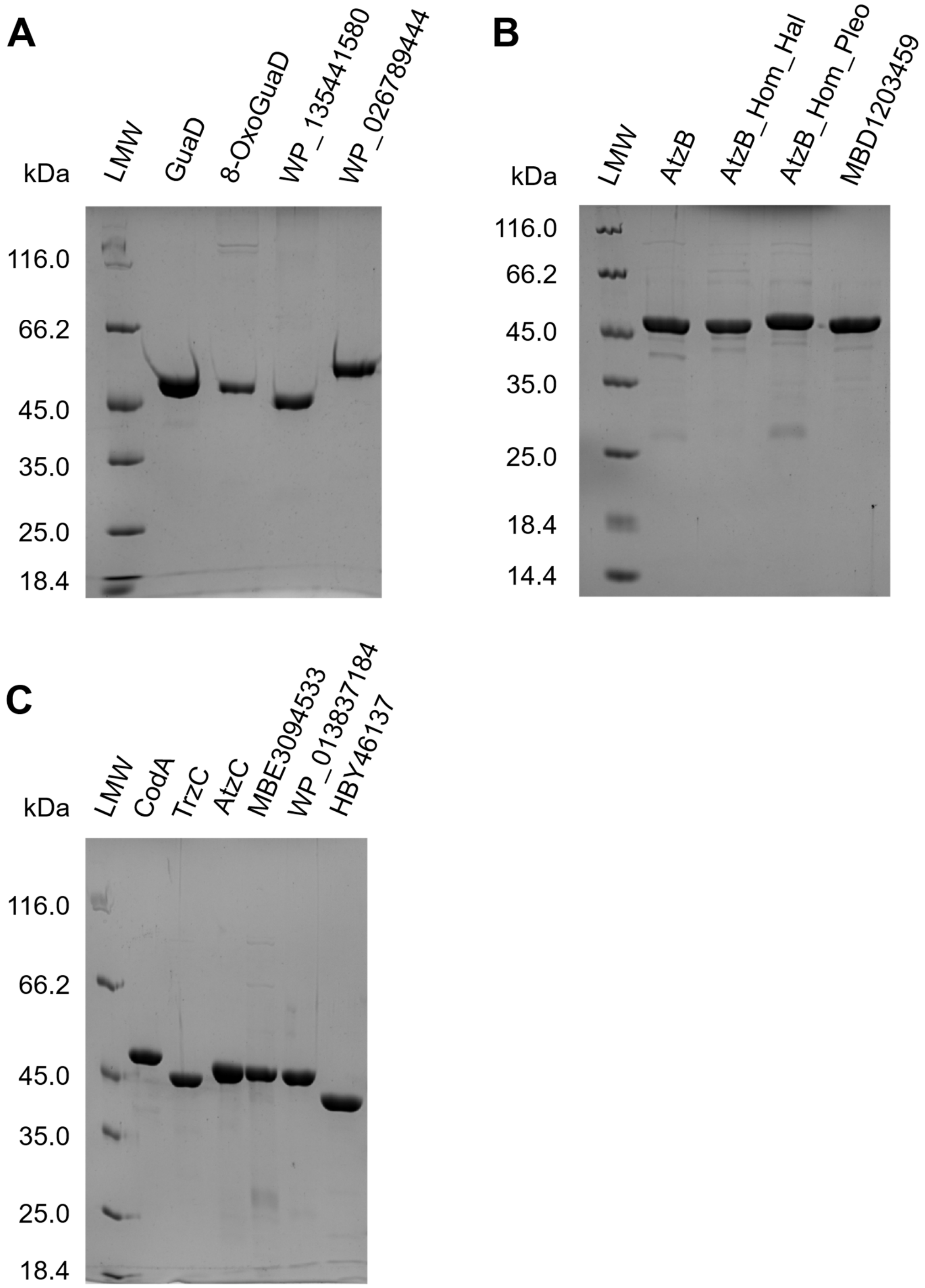

Figure S3: Assessment of the purity of the experimentally characterized AHS enzymes. After enrichment by IMAC and SEC, the purity of the proteins (3 µg each) was assessed by SDS-PAGE using LMW protein standard (Thermo Fisher Scientific). (**A**, **B**) Assessment of the purity of GuaD-Cluster enzymes: GuaD from *Escherichia coli*, 8-OxoGuaD from *Pseudomonas aeruginosa*, WP_135441580 from *Haliea sp*. SAOS-164, WP_026789444 from *Pleomorphomonas oryzae,* AtzB from *Pseudomonas sp.* strain ADP, AtzB_Hom_Hal from *Haliea sp*. SAOS-164, AtzB_Hom_Pleo from *Pleomorphomonas oryzae*, and MBD1203459 from *Rhodobacteraceae bacterium*. (**C**) Assessment of the purity of CodA-cluster enzymes: CodA from *Escherichia coli*, TrzC from *Paracidovorax citrulli*, AtzC from *Pseudomonas sp.* strain ADP, MBE3094533 from *Actinobacteria bacterium*, WP_013837184 from *Novosphingobium sp*. PP1Y, and HBY46137 from *Chloroflexi bacterium*.

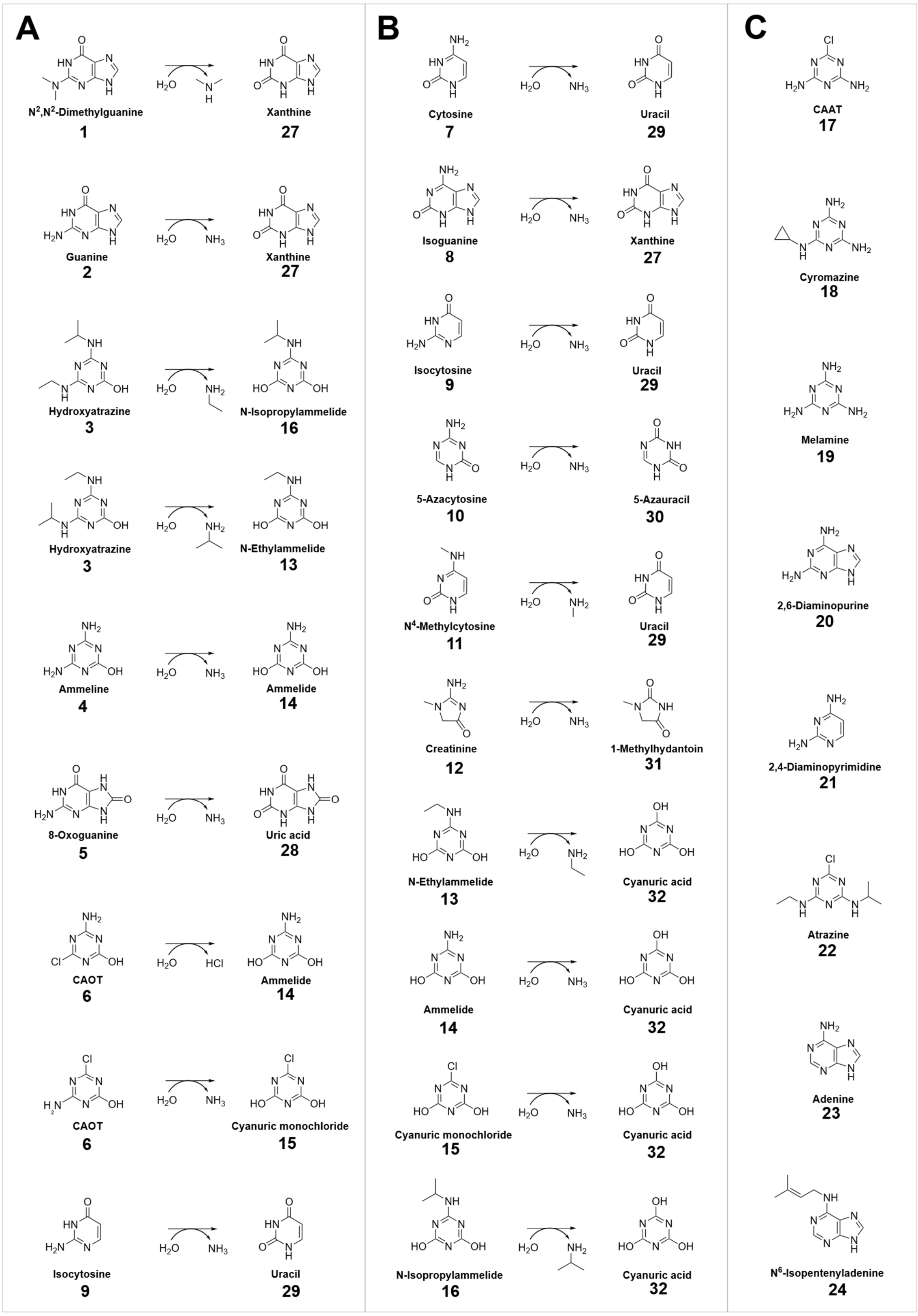

Figure S4: Substrate profiling of various AHS enzymes using HPLC-based enzymatic assays (**Source Data 1-24**) revealed (**A**) nine different hydrolysis reactions for GuaD-cluster enzymes and (**B**) ten for CodA-cluster enzymes. (**C**) Compounds **17**-**24** were not hydrolyzed by any of the enzymes tested.

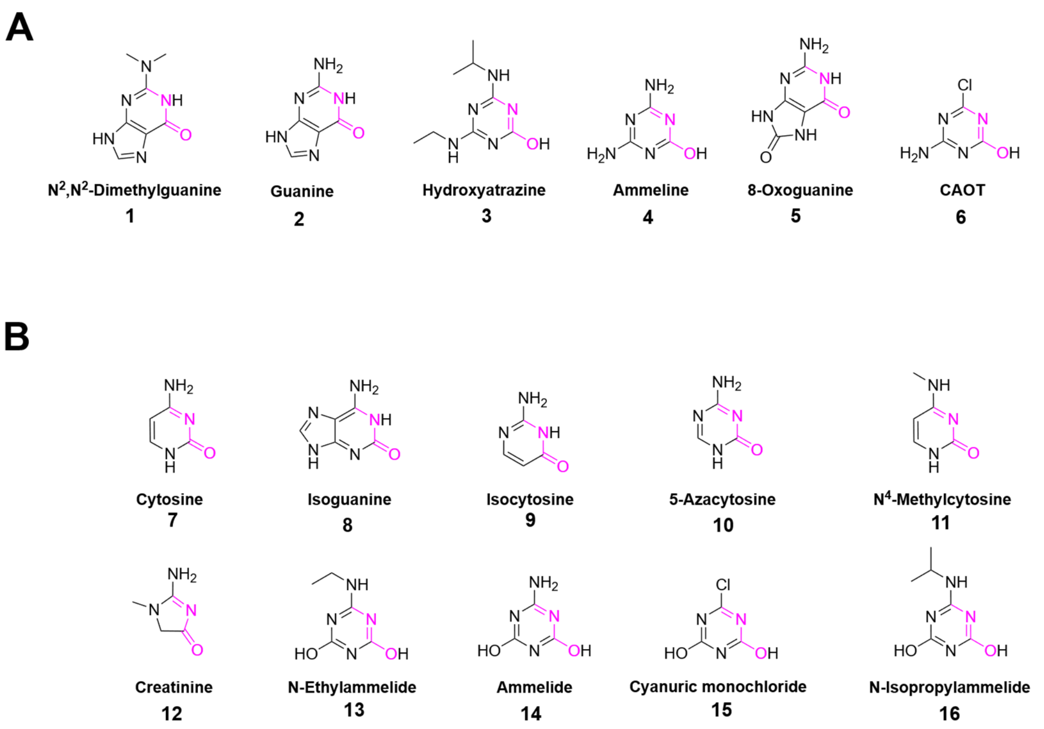

Figure S5: Comparison of the structural constitution of the tested substrates. Shown are compounds converted by (**A**) GuaD-cluster enzymes and (**B**) CodA-cluster enzymes. All compounds share a common conserved C-N-C-O linkage marked in pink. Apart from this molecular motif, all compounds are highly diverse: Both groups of compounds (**A** and **B**) include *s*-triazines, purines, and pyrimidines, while the leaving group (upper ring substituent) can either result in the formation of NH_3, NH_2-R (R = alkyl), R^1NH-R^2 (R^1, R^2 = alkyl), or Cl^-. Furthermore, each substrate possesses an exocyclic oxygen (right ring substituent in meta position to the leaving group). The third substituent (left ring substituent in meta position to the leaving group) can again be highly diverse among both groups of compounds (**A** and **B**) including C-H, C-N, and C-O bonds. Hence, all substrates are as diverse within one group as they are between the two groups.

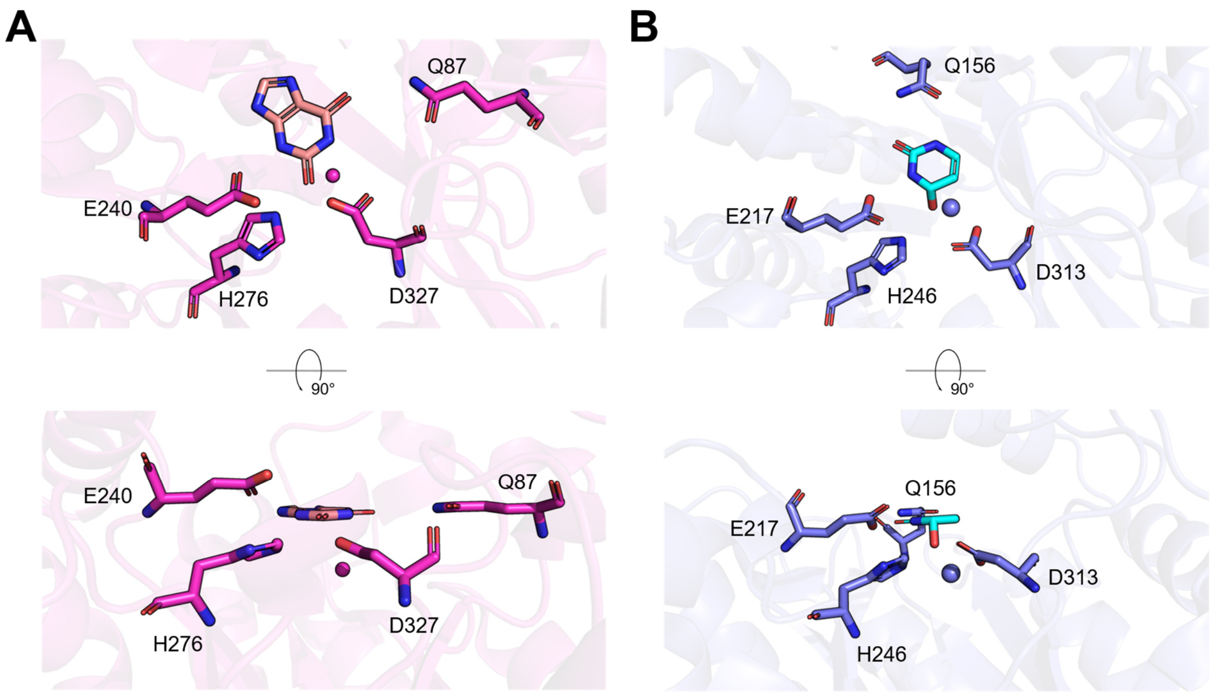

Figure S6: Active site composition of guanine deaminases compared to cytosine deaminases. (**A**) Active site of *E. coli* guanine deaminase (PDB: 6OHB). The product xanthine was inferred from an overlay with *S. cerevisiae* guanine deaminase (6OHA). (**B**) Active site of *E. coli* cytosine deaminase with bound transition-state inhibitor 4-hydroxy-3,4-dihydro-1H-pyrimidin-2-one mimicking the FCI (PDB: 1K70). Residues involved in catalysis as shown in **Figure 3** are depicted as sticks and divalent metal ions are shown as spheres. While the positioning of residues involved in water nucleophile activation (D327 in 6OHB and D313 in 1K70), proton transfer (H276 in 6OHB and H246 in 1K70), and substrate protonation (E240 in 6OHB and E217 in 1K70) is highly fixed (RMSD = 0.226, superposition of 16 to 16 atoms) between both enzymes, the main difference in active site composition is the varying position of the glutamine residue involved in stabilization of the FCI oxyanion (Q87 in 6OHB and Q156 in 1K70). While Q87 in 6OHB succeeds β-strand 1 of the $(\beta\alpha)_8$-barrel core, Q156 in 1K70 succeeds β-strand 3. In both enzymes, the substrate protonating glutamate as well as the FCI stabilizing glutamine lie in the same plane as the ligand. However, the water nucleophile activating aspartate is coordinated to the divalent metal ion and is positioned, similar to the bridging histidine residue, beneath the ring system of the ligand. This active site architecture unambiguously defines the direction from which the water nucleophile, which is coordinated to the metal ion as well as the aspartate, is approaching the ring of a substrate.

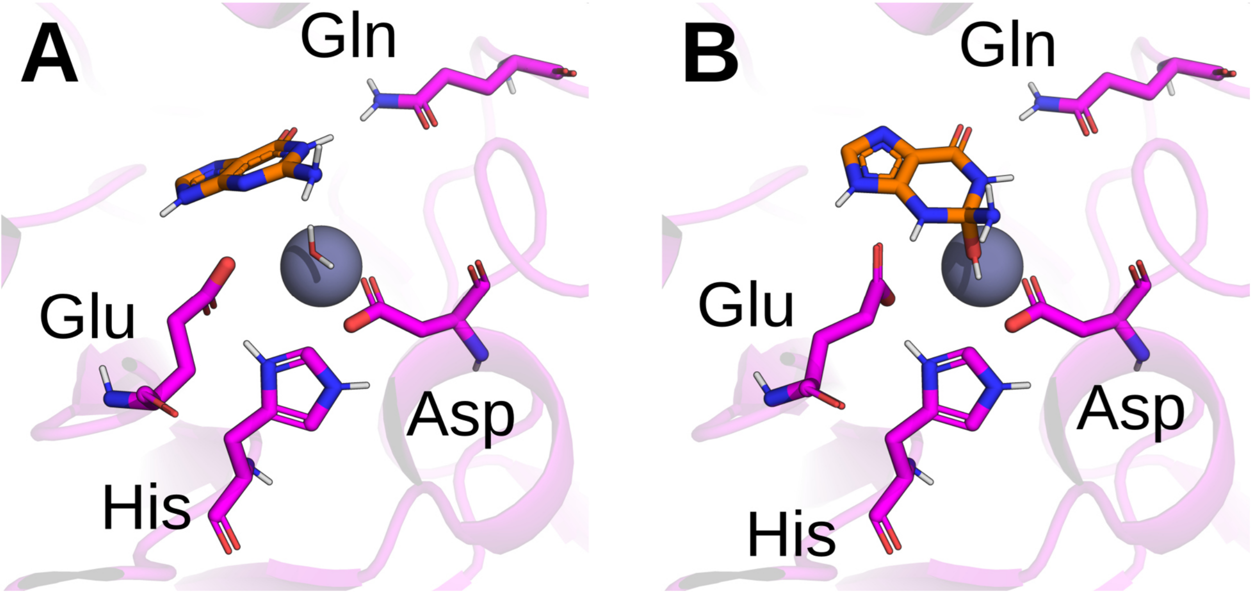

Figure S7: Computational modeling of the active site architecture of guanine deaminase. (**A**) Active site of *E. coli* guanine deaminase in the pre-reaction state I (cf. Figure 3A-I) with bound native substrate guanine (sticks, orange carbon atoms, dark blue nitrogen atoms, red oxygen atoms, white hydrogen atoms). Key residues (sticks, magenta carbon atoms), the attacking water molecule (lines), and the zinc ion (spheres, dark gray) that are involved in catalysis are highlighted. (**B**) Active site of *E. coli* guanine deaminase in the FCI-state III (cf. Figure 3A-III) with bound guanine-FCI. Both states were computationally modeled based on the X-ray crystallography structure of *E. coli* guanine deaminase (PDB: 6OHC). These structural models were energy optimized and subsequently relaxed using physics based molecular mechanics force field approaches as detailed in the Materials and Methods section.

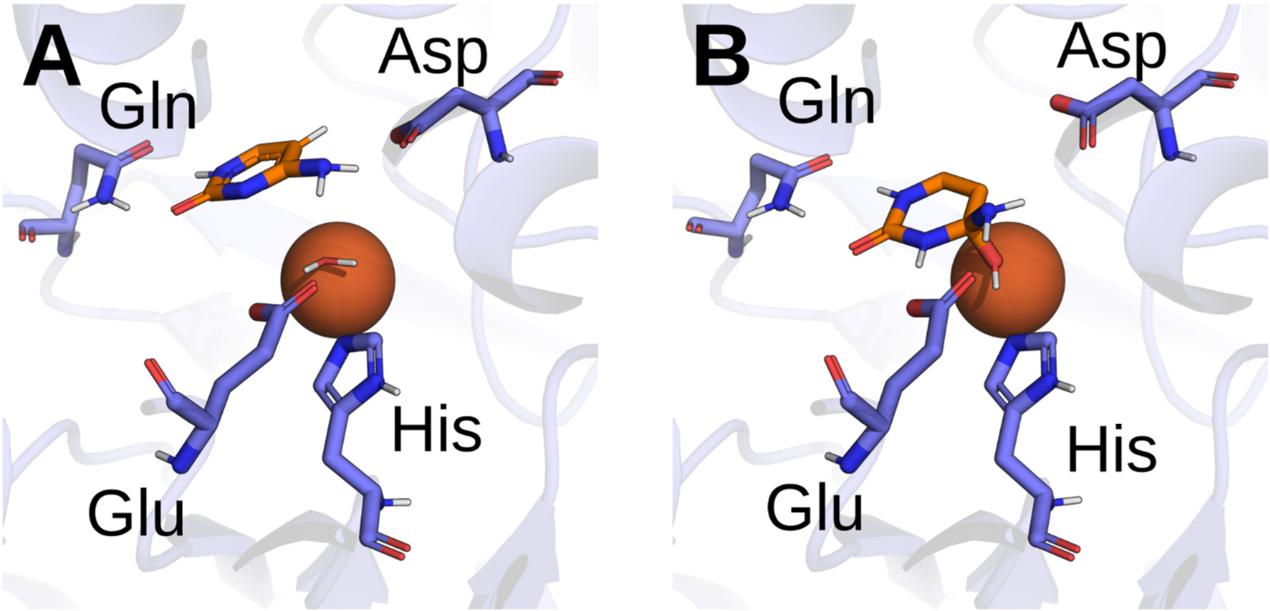

Figure S8: Computational modeling of the active site architecture of cytosine deaminase. (**A**) Active site of *E. coli* cytosine deaminase in the pre-reaction state I (cf. Figure 3B-I) with bound native substrate cytosine (sticks, orange carbon atoms, dark blue nitrogen atoms, red oxygen atoms, white hydrogen atoms). Key residues (sticks, light blue carbon atoms), the attacking water molecule (lines), and the iron ion (spheres, dark orange) that are involved in catalysis are highlighted. (**B**) Active site of *E. coli* cytosine deaminase in the FCI-state III (cf. Figure 3B-III) with bound cytosine-FCI. Both states were computationally modeled based on the X-ray crystallography structure of *E. coli* cytosine deaminase (PDB: 1K70). These structural models were energy optimized and subsequently relaxed using physics based molecular mechanics force field approaches as detailed in the Material and Methods section.

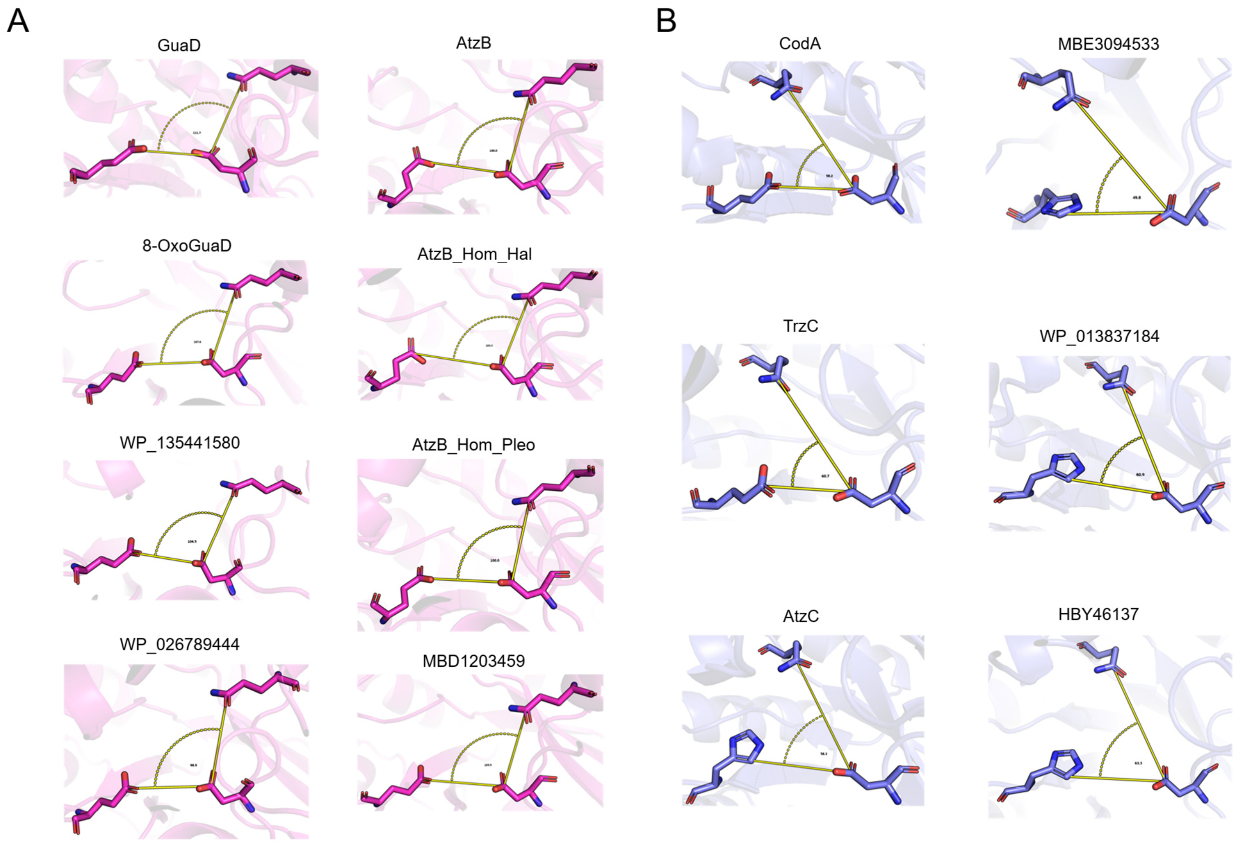

C

	GuaD	8-OxoGuaD	WP_135441580	WP_026789444	AtzB	AtzB_Hom_Hal	AtzB_Hom_Pleo	MBD1203459	CodA	TrzC	AtzC	MBE3094533	WP_013837184	HBY46137
**angle**	**111.7°**	**107.6°**	**104.5°**	**98.9°**	**100.0°**	**103.3°**	**100.0°**	**103.5°**	**58.2°**	**60.7°**	**58.3°**	**49.8°**	**60.9°**	**63.3°**

Figure S9: Active site composition of (A) GuaD-cluster and (B) CodA-cluster enzymes and angles between catalytic residues. For GuaD (PDB: 6OHB), CodA (PDB: 1K70), and AtzC (PDB: 4CQB) crystal structures are available, while for all other proteins AlphaFold2 structures were used. Depicted in sticks are those residues that execute water nucleophile activation (Asp), substrate protonation (Glu/His), and FCI stabilization (Gln), whereas the bridging proton-shuttling histidine is not shown. In some enzymes (MBE3094533, WP_013837184, AtzC, HBY46137) the substrate protonating Glu is replaced by a second His residue having the same function, as already known for AtzC.[22,23] All eight enzymes selected from the GuaD-cluster possess an equivalent glutamine residue, corresponding to the one shown in **Figure 3A** and **Figure S6A**. Similarly, the six enzymes selected from the CodA-cluster possess an equivalent glutamine residue, corresponding to the one shown in **Figure 3B** and **Figure S6B**. (**C**) The Glu/His-Asp-Gln angles of GuaD-cluster proteins fall within a range of 98.9° to 111.7°, while for CodA-cluster proteins, this angle lies between 49.8° and 63.3°.

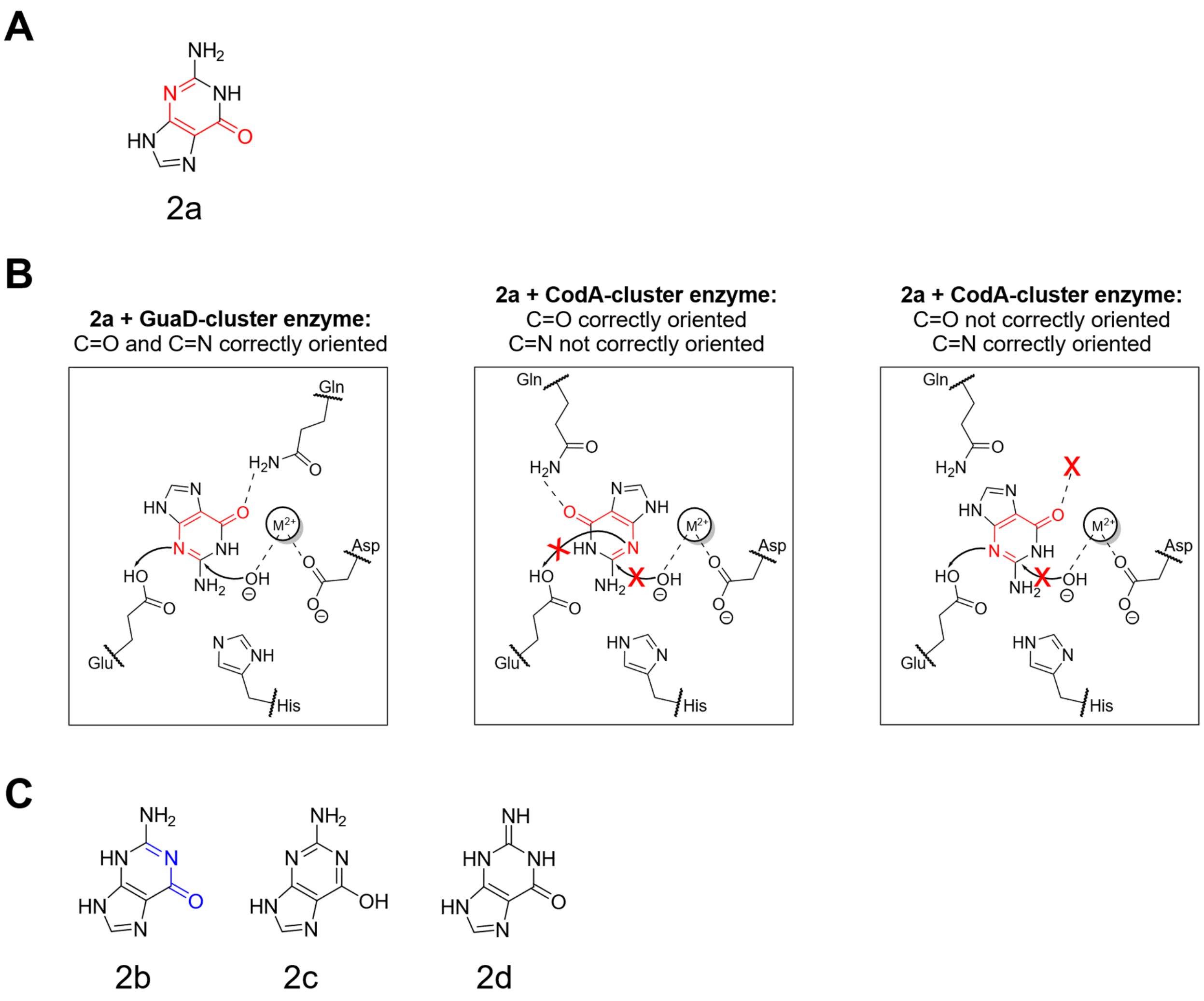

Figure S10: (**A**) Tautomer **2a** of guanine exhibits a 6-π-electron system (indicated in red) and corresponds to the most reactive and most prevalent tautomeric form as shown in **Figure 5A**. (**B**) Tautomer **2a** can be accepted by GuaD-cluster enzymes (left panel, **Figure 3**, **Figure S7**), while it cannot be accepted by CodA-cluster enzymes due to incorrect orientation of either the electrophilic C=N double bond (middle panel, **Figure S11B**) or the exocyclic carbonyl (right panel). Hence, either the water nucleophile cannot attack the C=N double bond at the Bürgi-Dunitz angle (107°) and the nitrogen (N3) of the C=N double bond cannot be protonated (middle panel), or the exocyclic carbonyl is not activated and an arising FCI not stabilized by the essential glutamine (right panel). Divalent metal ions are represented by M^{2+}. (**C**) **2b**, **2c**, and **2d** are further conceivable tautomers of guanine **2**. Although tautomer **2b** exhibits a 4-π-electron system (indicated in blue) similarly to CodA-cluster substrates, a turnover of guanine **2** by CodA-cluster enzymes was not observed, consistent with the observation that **2b** constitutes a minor tautomeric form.[24] In addition to their minor occurrence, tautomers **2c** and **2d** either do not occur in a reactive keto form (**2c**) or do not possess a C=N double bond that can be attacked by the nucleophile at the Bürgi-Dunitz angle (**2d**).

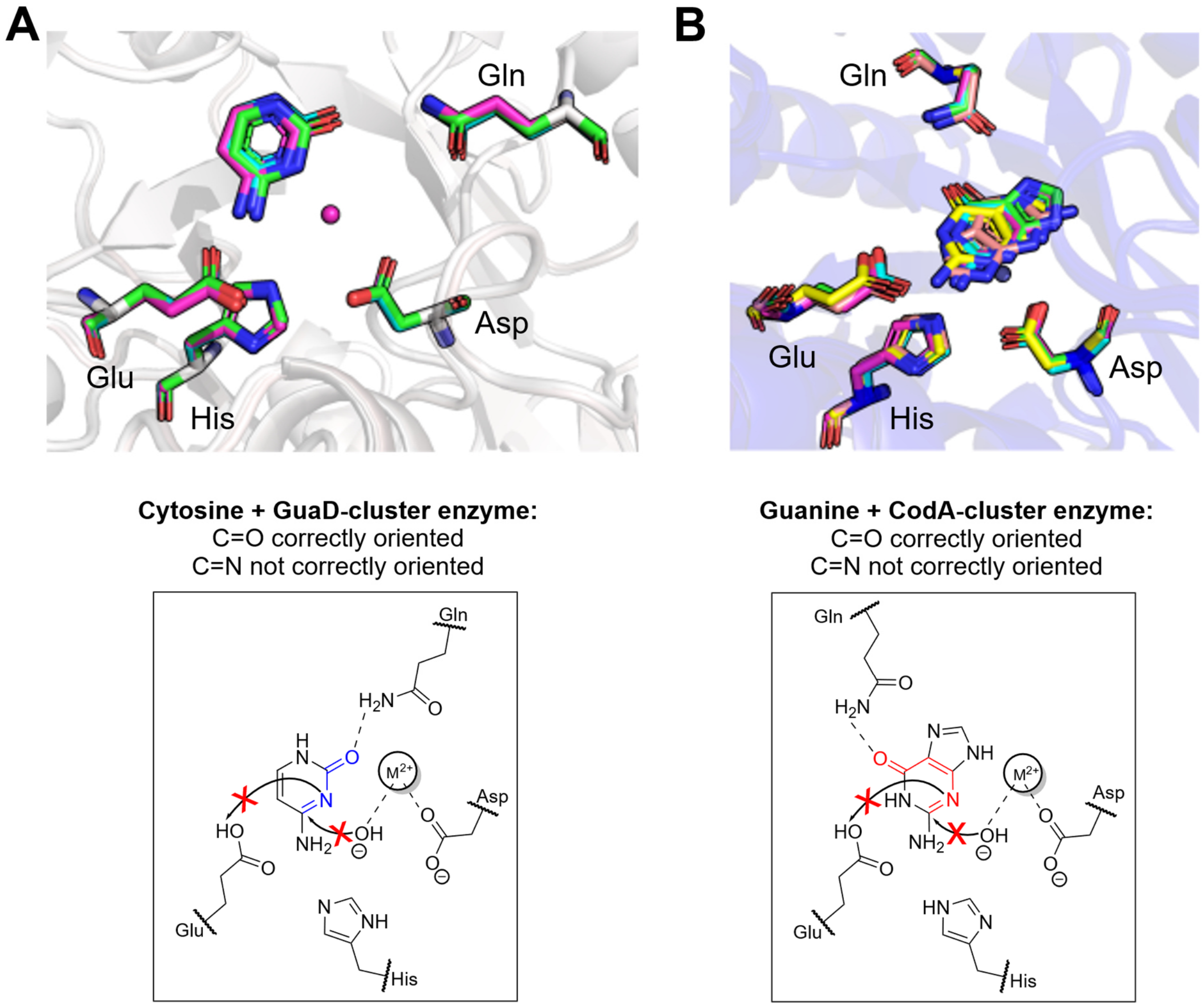

Figure S11: Unproductive substrate positioning in GuaD and CodA. (**A**) Superposition of the three best ranked Chai-1[7] predictions suggests an orientation of cytosine in *E. coli* guanine deaminase that is unproductive for catalysis. (**B**) Superposition of the five best ranked Chai-1[7] predictions suggests an orientation of guanine in *E. coli* cytosine deaminase that is unproductive for catalysis. This is due to incorrect orientation of the electrophilic C=N double bond. Hence, the water nucleophile cannot attack the C=N double bond at the Bürgi-Dunitz angle (107°) and the nitrogen (N3) of the C=N double bond cannot be protonated. Residues involved in catalysis are depicted as sticks and divalent metal ions are shown as spheres or represented by M^{2+}. The Chai-1 predictions show the most likely substrate orientations and indicate that the substrate's exocyclic oxygen is positioned close to the crucial glutamine in each enzyme, explaining why guanine can be hydrolyzed by guanine deaminases but not by cytosine deaminases and *vice versa*. Moreover, even a differently oriented substrate with the C=N double bond correctly oriented (cf. Figure S10B right panel) would be accompanied by an incorrect orientation of the exocyclic carbonyl (cf. Table S1).

A

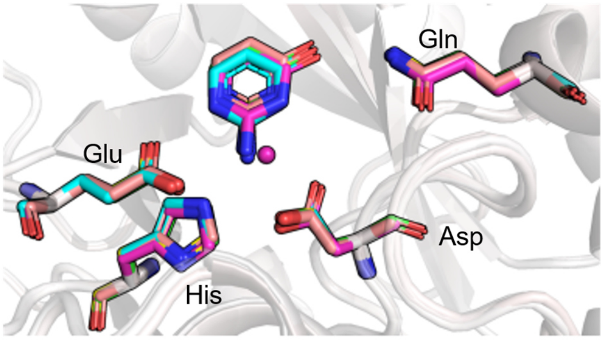

3*H*-Isocytosine (9a) + GuaD-cluster enzyme:
C=O correctly oriented
C=N correctly oriented

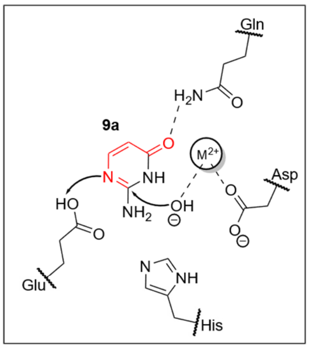

1*H*-Isocytosine (9b) + GuaD-cluster enzyme:
C=O correctly oriented
C=N not correctly oriented

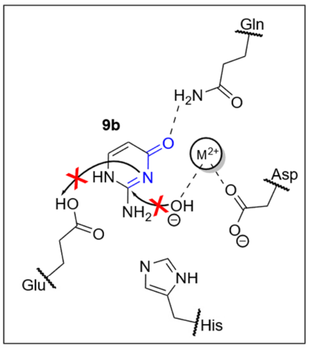

B

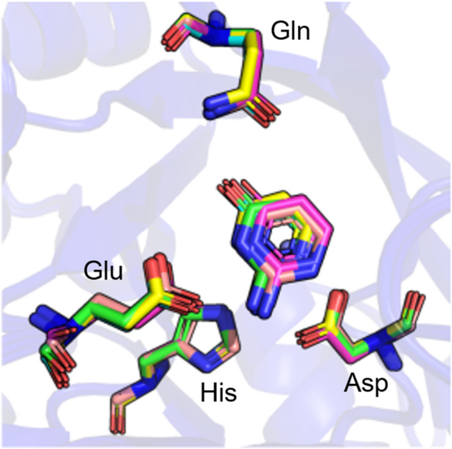

1*H*-Isocytosine (9b) + CodA-cluster enzyme:
C=O correctly oriented
C=N correctly oriented

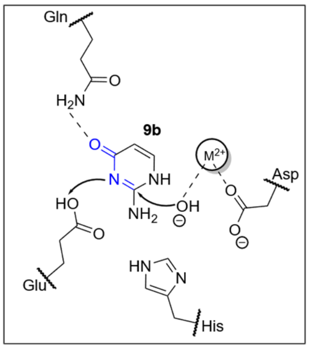

3*H*-Isocytosine (9a) + CodA-cluster enzyme:
C=O correctly oriented
C=N not correctly oriented

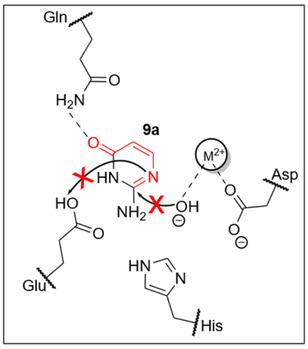

Figure S12: Positioning of isocytosine in (A) 8-OxoGuaD and (B) CodA. Superposition of the five best ranked Chai-1[7] predictions each suggests an orientation of isocytosine in 8-OxoGuaD that is flipped by 180° compared to CodA. These enzymes were chosen as representatives of the two SSN clusters, as they showed the highest activities for isocytosine. Residues involved in catalysis are depicted as sticks and divalent metal ions are shown as spheres or represented by M^{2+}. In GuaD-cluster enzymes, only **9a** possessing a 6-π-electron system is correctly oriented for productive hydrolysis (**A**), whereas in CodA-cluster enzymes, only **9b** possessing a 4-π-electron system is correctly oriented for productive hydrolysis (cf. Table S1). These findings align with the observation that GuaD-cluster enzymes proceed via 1,6 nucleophilic conjugate addition, whereas CodA-cluster enzymes proceed via 1,4 nucleophilic conjugate addition, which is shown in detail in **Figure S13**.

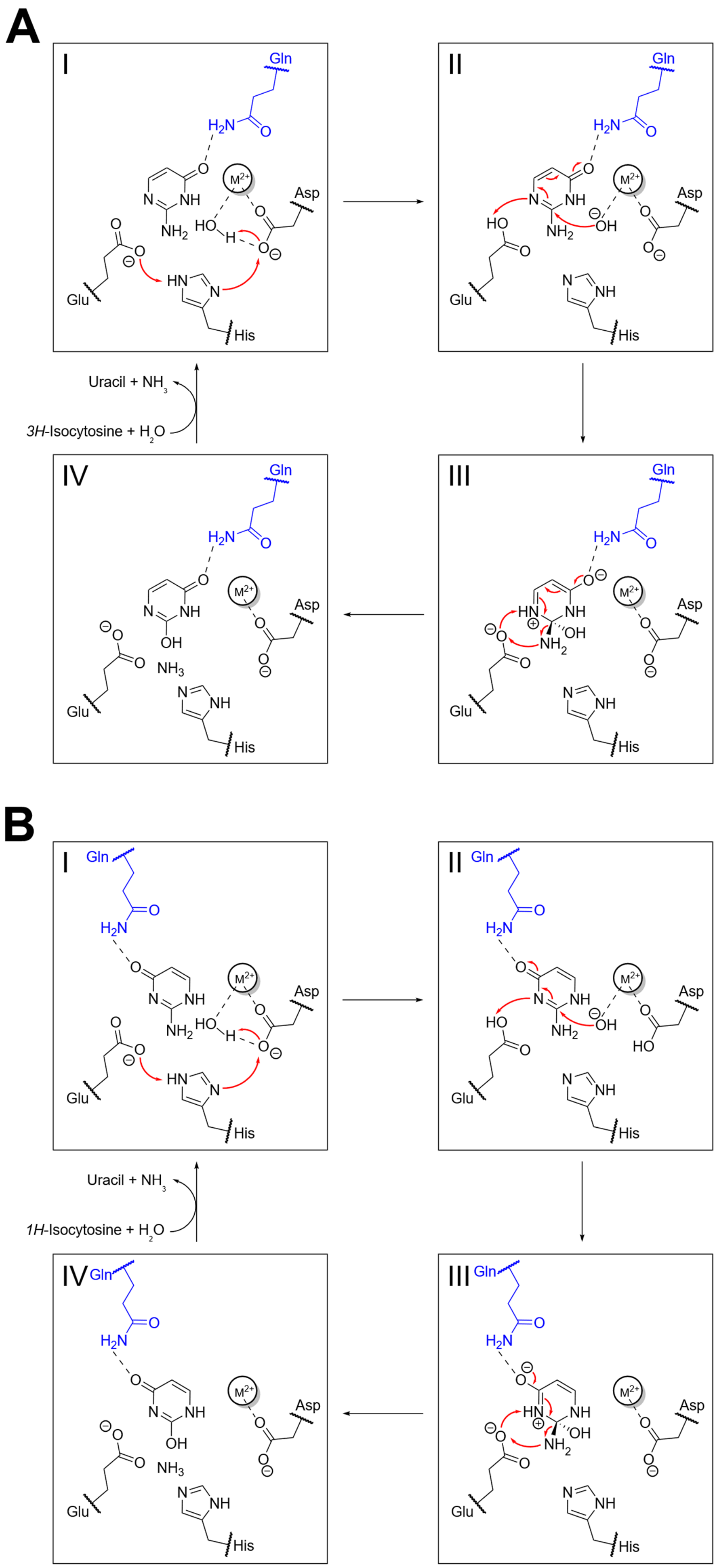
A
I
II
III
IV
Gln
Asp
Glu
His
M2+
H2N
Uracil + NH3
3H-Isocytosine + H2O
B
1H-Isocytosine + H2O

Figure S13: Reaction mechanism for the deamination of isocytosine in (A) GuaD-cluster enzymes and (B) CodA-cluster enzymes. Both reaction mechanisms proposed here can be divided into four steps: **I**: While the GuaD-cluster enzymes can target **9a** bearing a 6 π-system, the CodA-cluster enzymes can target **9b** bearing a 4 π-system (cf. Figure 5A, Figure S12). The differing position of the essential glutamine (indicated in blue) in the two enzyme groups goes along with a 180°-flipped orientation of the isocytosine ring in the respective active site. In both groups, the water nucleophile is deprotonated by an aspartate and the proton is shuttled from the aspartate to a glutamate by a bridging histidine. **II**: Isocytosine is protonated by the glutamate and the nucleophile attacks the C=N double bond of isocytosine at the Bürgi-Dunitz angle (107°) through a (**A**) 1,6 conjugate addition or a (**B**) 1,4 conjugate addition. **III**: A new stereocenter is formed within the FCI. Shown is the zwitterionic resonance structure of the FCI in which the oxyanion is stabilized by a glutamine. The glutamate deprotonates the ring nitrogen and protonates the amine substituent resulting in a backflow of π-electrons. **IV**: The leaving group is released and the product uracil is formed. Electron flows and divalent metal ions are represented by red arrows and M^{2+}, respectively. From the currently available literature[19], it is uncertain whether the proton shuttle occurs before or after the substrate binds to the active site. Moreover, it is highly probable that the glutamate is deprotonated at the beginning of the reaction, prior to the initiation of the proton shuttle (**I**). Otherwise, a likewise protonated glutamate would result in significant clashes with the FCI (**III**). These facts, however, would not change the overall reaction mechanism.

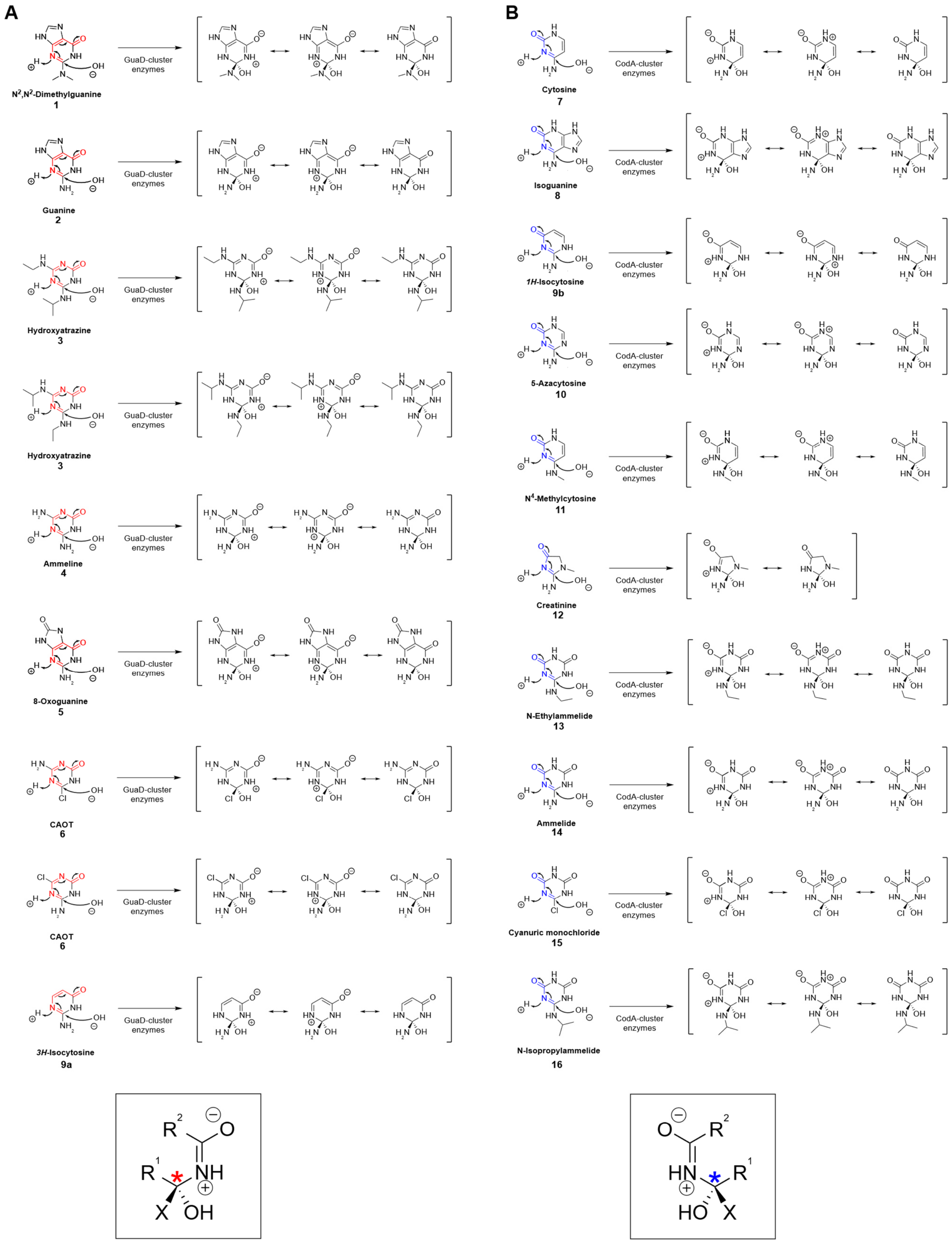

core configuration
fleeting chiral intermediate
GuaD-cluster enzymes

core configuration
fleeting chiral intermediate
CodA-cluster enzymes

Figure S14: Illustration of the absolute configuration of the FCIs for all detected reactions. (**A**) Catalysis within GuaD-cluster enzymes proceeds through a 1,6 nucleophilic conjugate addition to a 6-π-electron system resulting in the formation of resonance stabilized FCIs with distinct core configuration involving the conserved molecular motif as shown in **Figure S5**. (**B**) Catalysis within CodA-cluster enzymes proceeds through a 1,4 nucleophilic conjugate addition to a 4-π-electron system resulting in the formation of resonance stabilized FCIs with distinct core configuration involving the conserved molecular motif as shown in **Figure S5**. These FCI core configurations of GuaD- and CodA-cluster enzymes are mirror images and are exemplarily shown as zwitterions (**Figure 5C**).

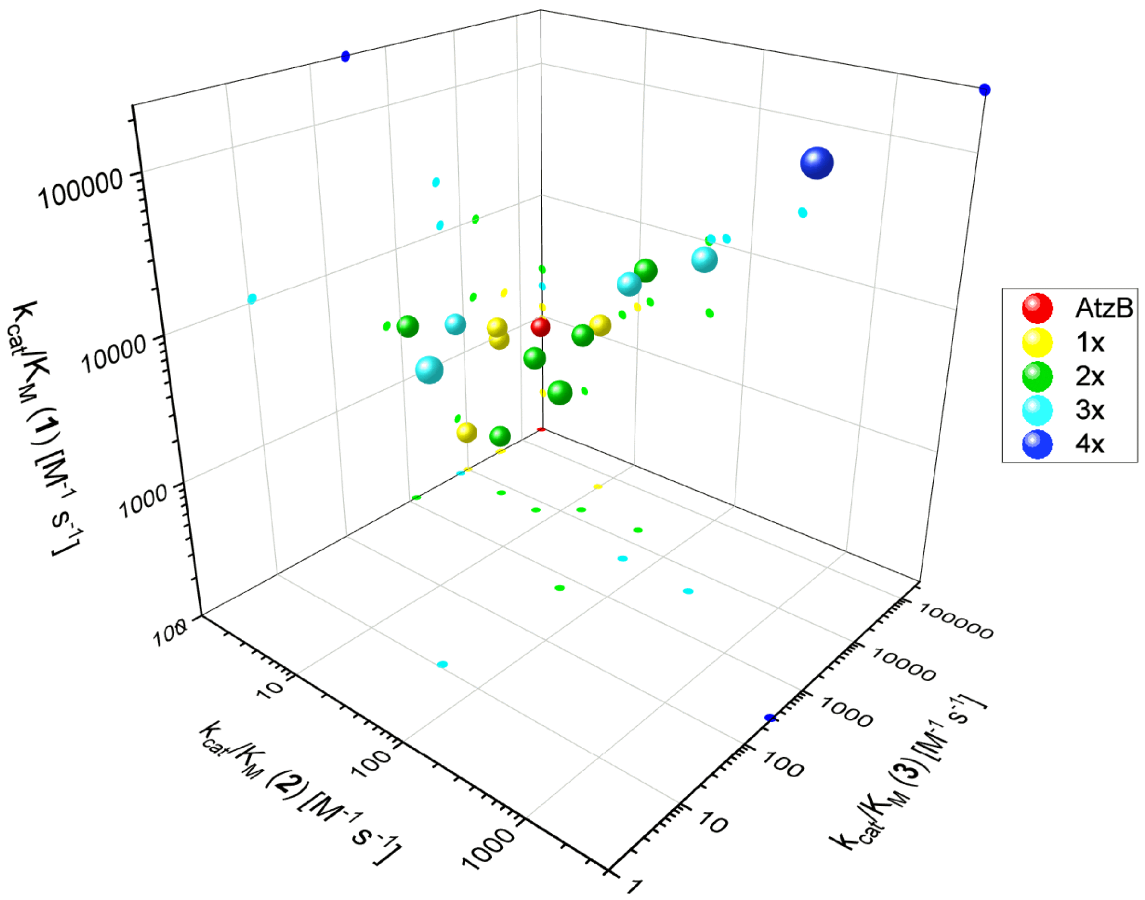

Figure S15: Catalytic efficiencies at 25°C for the hydrolysis of N^2,N^2-dimethylguanine 1, guanine 2, and hydroxyatrazine 3 by AtzB wildtype and variants. The red sphere corresponds to AtzB wildtype, while yellow/green/cyan/blue spheres correspond to single/double/triple/ quadruple mutants. The shown data are taken from **Table S2**. Both **1**, **2**, and **3** possess a conjugated 6-π-electron system (cf. Figure 5A) targeted by GuaD-cluster enzymes via 1,6 nucleophilic conjugate addition.

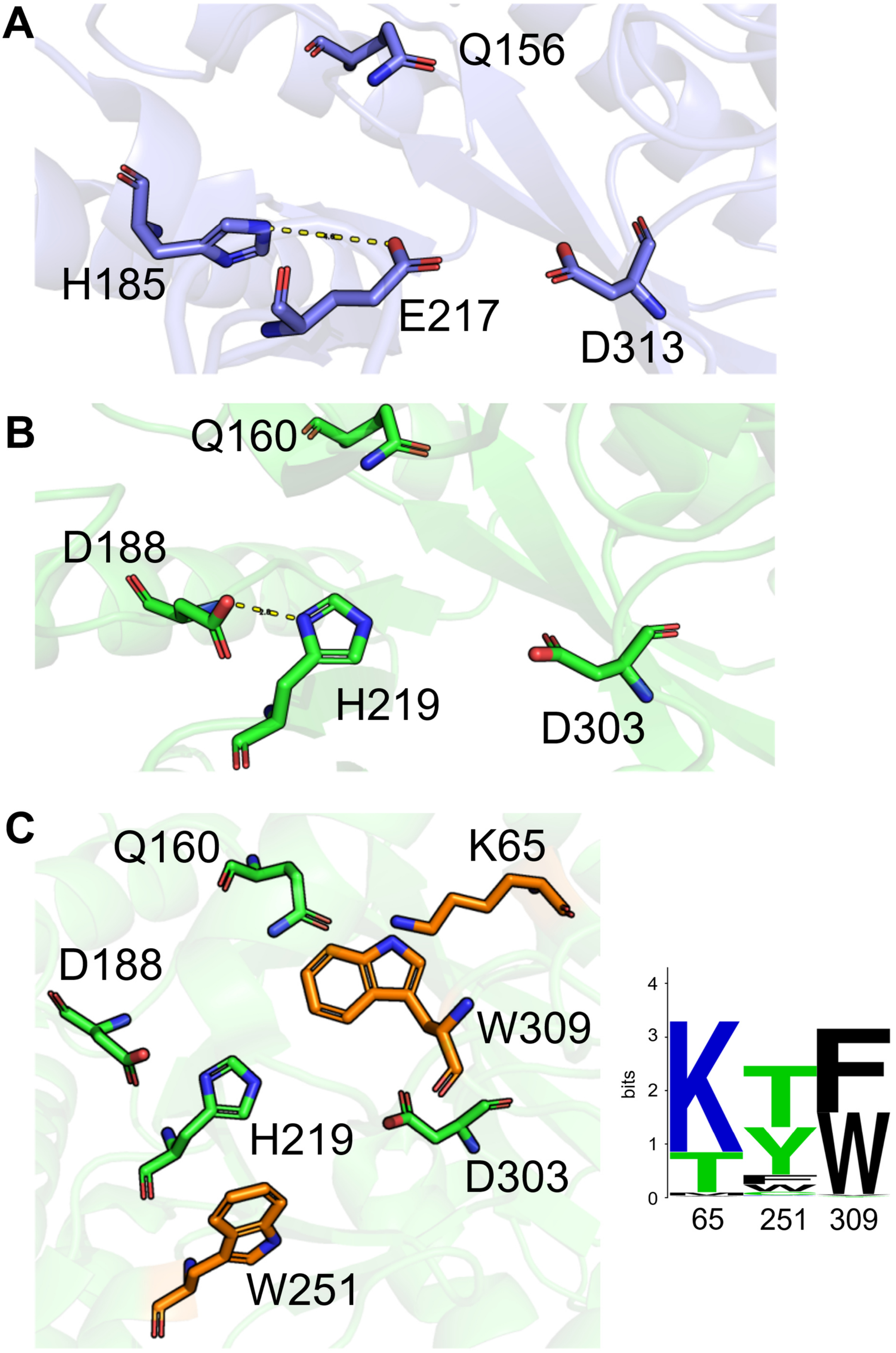
A
Q156
H185
E217
D313
B
Q160
D188
H219
D303
C
Q160
K65
D188
W309
H219
D303
W251
bits
4
3
2
1
0
65
251
309

Figure S16: AtzC mutational planning. (**A**) Active site of CodA (PDB: 1K70) in which D313 activates the water nucleophile, Q156 stabilizes the oxyanion FCI, and E217 mediates protonation of the substrate. Residue H185 is in hydrogen bond distance to E217, thereby forming a catalytic dyad and likely decreasing the pk_a of E217. (**B**) Active site of AtzC (PDB: 4CQB) in which D303 activates the water nucleophile and Q160 stabilizes the oxyanion FCI. Substrate protonation is mediated by H219, which occupies the position corresponding to E217 in CodA. Similarly, D188 in AtzC resembles H185 in CodA and is in hydrogen bond distance to H219, thereby forming a catalytic dyad and likely decreasing the pk_a of H219. To create AtzC variants that show altered substrate scopes, we focused on such active site residues that differ between AtzC and homologous enzymes. By comparing CodA (**A**) with AtzC (**B**) we proposed the H219E mutation in AtzC to be a plausible first step to alter substrate scope without disturbing the reaction mechanism. Then, the double mutant AtzC_D188H_H219E was designed with the aim to create a catalytic dyad similar to that in CodA. (**C**) Based on the double mutant AtzC_D188H_H219E three triple mutants were created that additionally target one of the active site residues that are depicted as orange sticks. In addition to the above discussed residues (depicted as green sticks), the AtzC active site is surrounded by further residues (K65, W251, W309, depicted as orange sticks) whose identities are different in homologous enzyme: Generation of a multiple sequence alignment of the 500 closest homologues of AtzC and subsequent creation of sequence logos for these three positions (positions 65/251/309 in AtzC) demonstrated that K65, T251, and F309 are the most frequently occurring residues, respectively. Hence, we mutated the respective residues in AtzC to these consensus residues (W251T, W309F). Since K65 (as found in AtzC) is already the most common amino acid at this position, we mutated this residue to the second most frequent amino acid (K65T). This yielded the three triple mutants AtzC_K65T_D188H_H219E, AtzC_D188H_H219E _W251T, and AtzC_D188H_H219E _W309F.

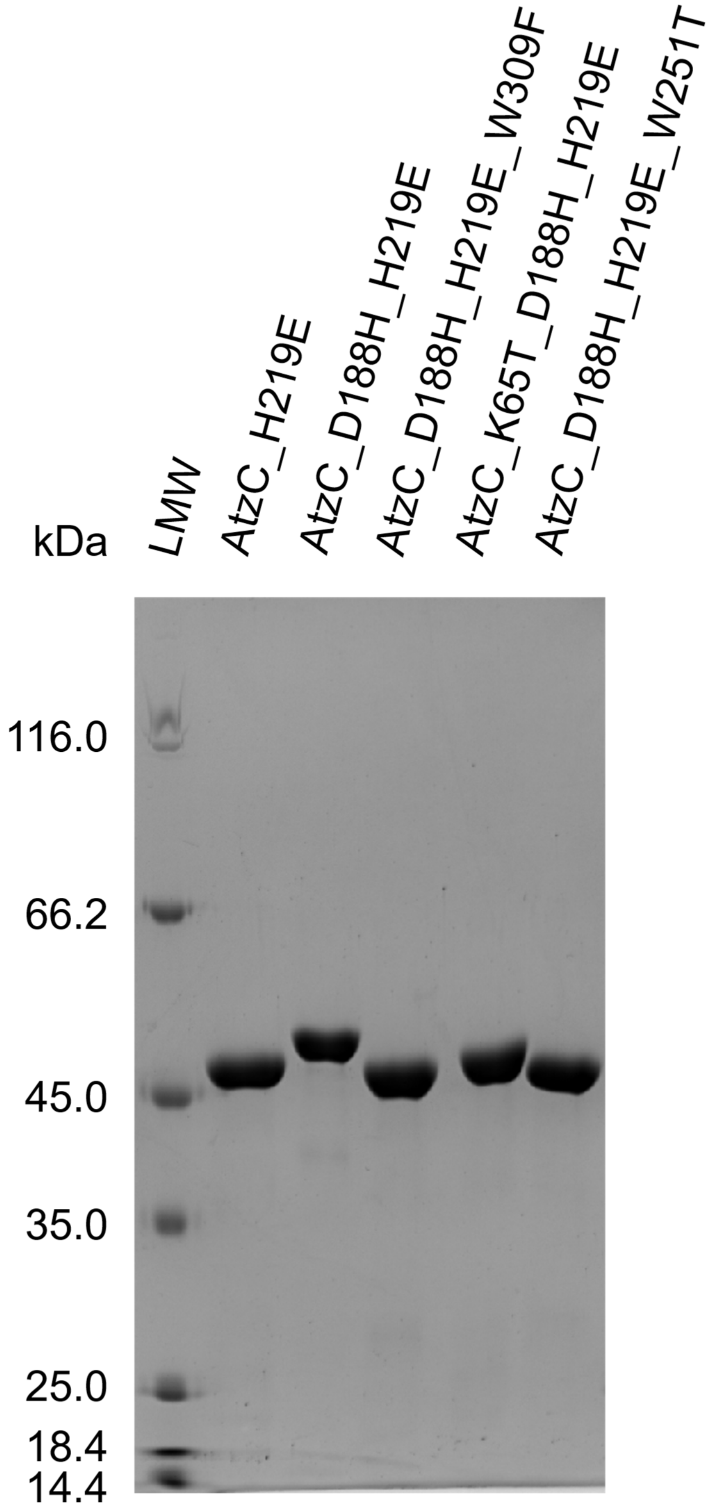

Figure S17: Assessment of the purity of AtzC mutants. After enrichment by IMAC and SEC, the purity of AtzC_H219E, AtzC_D188H_H219E, AtzC_D188H_H219E_W309F, AtzC_K65T_D188H_H219E, and AtzC_D188H_H219E_W251T (3 µg each) was assessed by SDS-PAGE using LMW protein standard (Thermo Fisher Scientific).

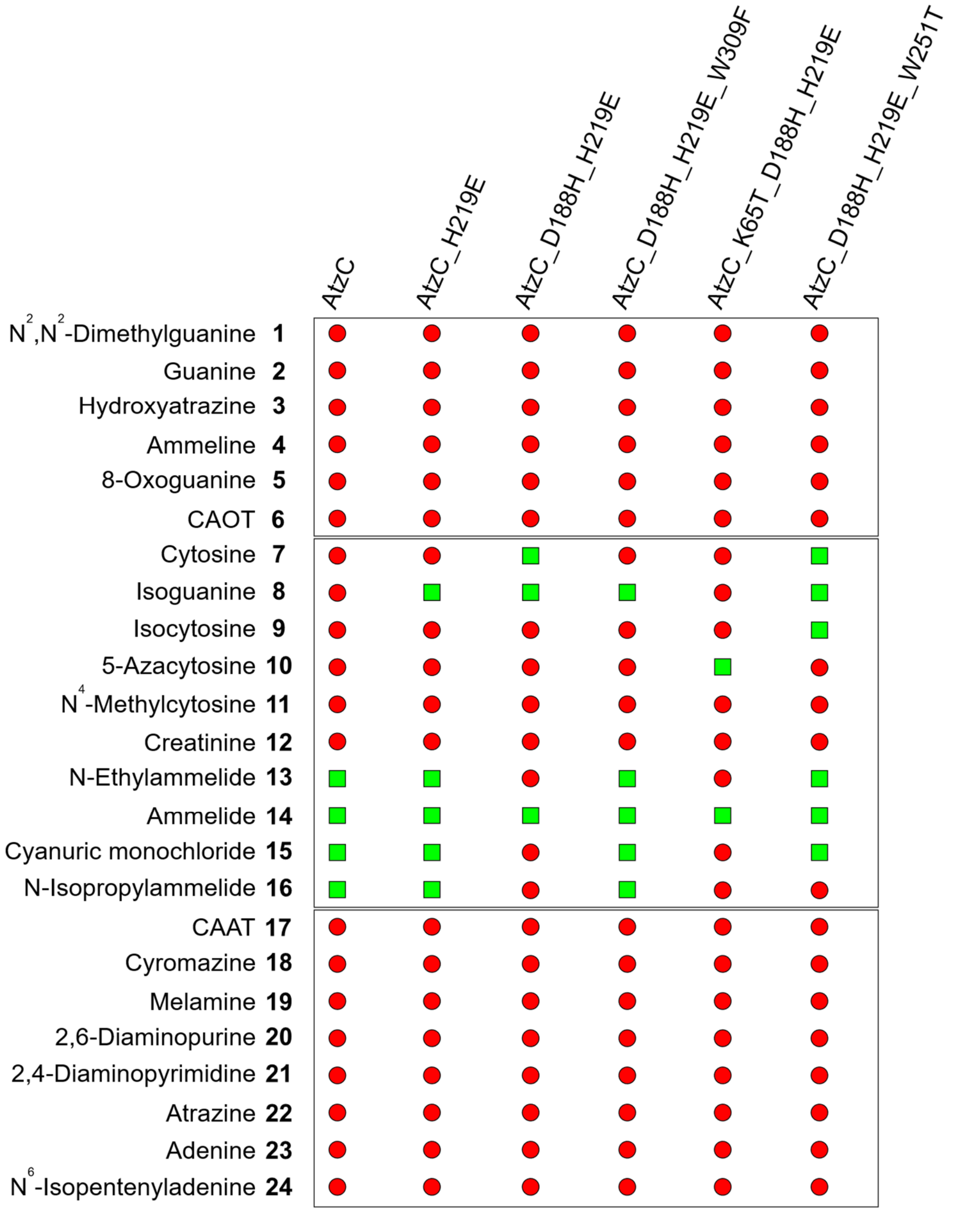

Figure S18: Substrate scope of AtzC variants in comparison to the wildtype enzyme. The tested substrate range comprised 24 natural and xenobiotic substances including purines, pyrimidines, and *s*-triazines. The underlying HPLC data are shown in **Source Data 25-48**. Red circles indicate enzyme-substrate pairs for which no turnover was detected, whereas light green squares indicate enzyme-substrate pairs for which turnover was detected. The AtzC variants match the substrate pattern from previously characterized enzymes from the CodA-cluster as they exclusively hydrolyze substrates bearing a 4-π-electronic composition (cf. Figure 5A) through a 1,4 nucleophilic conjugate addition (cf. Figure S14B). Moreover, this analysis showed that entirely new activities can easily arise within an enzyme through single mutations: AtzC_H219E has a new activity for compound **8** compared to the wildtype, the further mutation D188H in the AtzC_D188H_H219E double mutant enables the additional hydrolysis of **7**, and once again a further mutation is sufficient to include additional new substrates: Either K65T as in AtzC_K65T_D188H_H219E, which enables the hydrolysis of **10**, or W251T as in AtzC_D188H_H219E_W251T, which enables the hydrolysis of **9**. On the other hand, while gaining the ability to hydrolyze **7**, AtzC_D188H_H219E concurrently loses its ability to hydrolyze **13**, **15**, and **16**, which in turn is reverted in AtzC_D188H_H219E_W309F and partially reverted in AtzC_D188H_H219E_W251T.

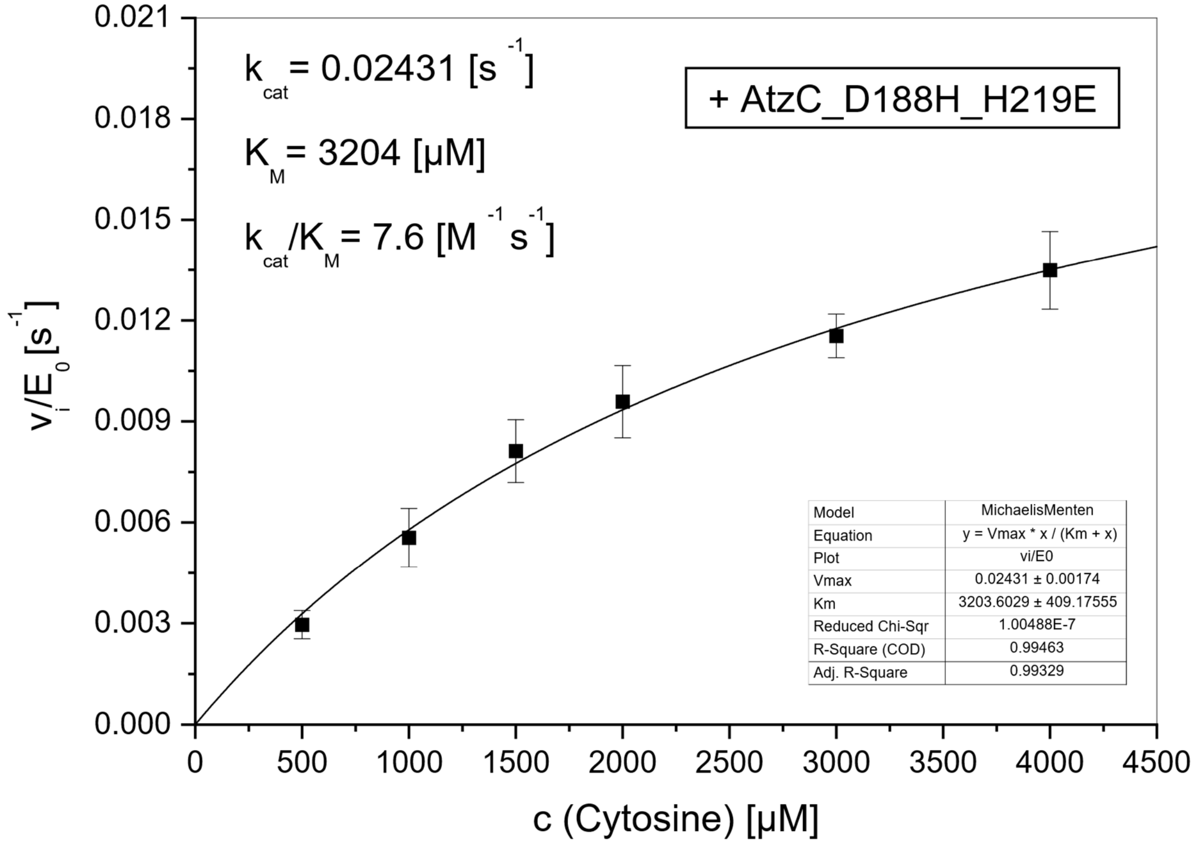

Figure S19: Steady-state enzyme kinetics of AtzC_D188H_H219E with cytosine 7 as substrate. Assay conditions were 50 mM KP buffer (pH 7.5), 10 µM AtzC_D188H_H219E, a variable concentration of cytosine ranging from 500 - 4000 µM, 25 °C. Triplicate measurements were performed at 286 nm using a differential molar extinction coefficient between cytosine and uracil of 680 M^{-1} cm^{-1}.[19] Determined initial velocities v_i were normalized to the total enzyme concentration E_0 and plotted against the substrate concentration. The data was fitted to a hyperbolic saturation curve with Origin (2022), yielding a catalytic efficiency of 7.6 M^{-1} s^{-1}.

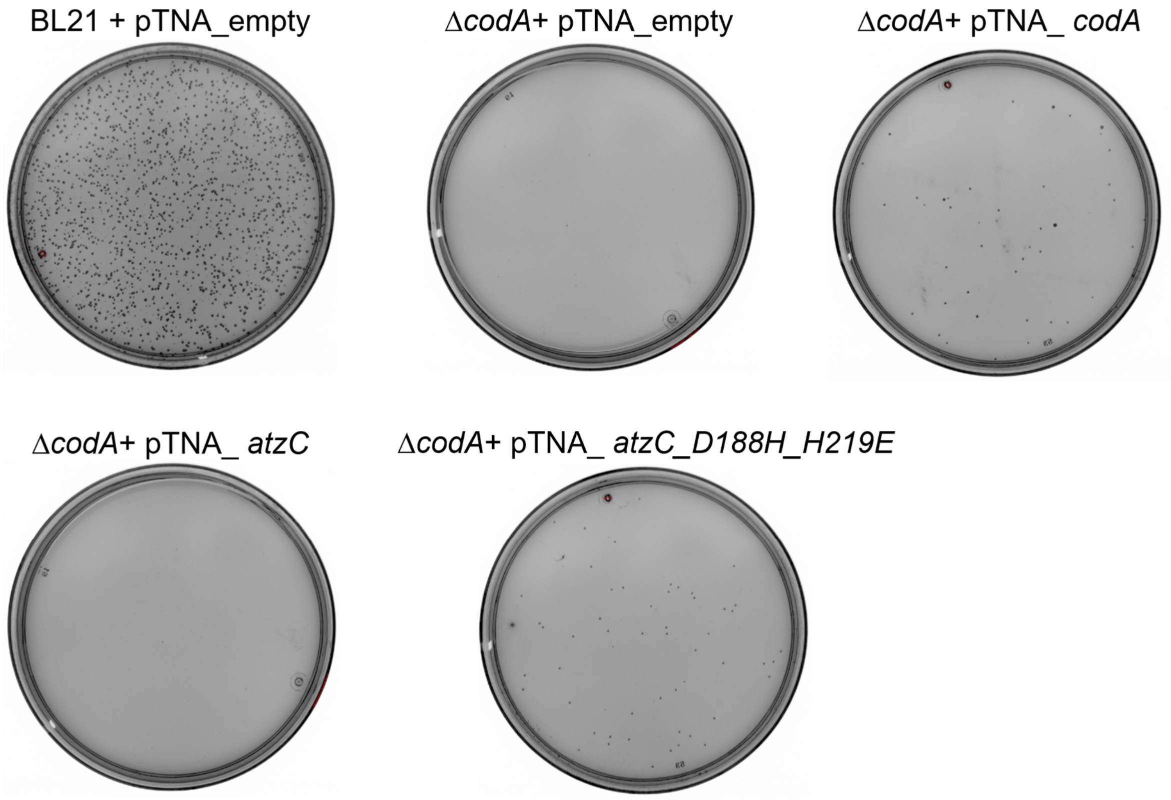

Figure S20: Assessment of cytosine deaminase activity *in vivo*. Growth assays were performed on M9 minimal media plates that contained cytosine (0.5 mg/ml) as sole nitrogen source. BL21 *E. coli* cells transformed with an empty pTNA vector (pTNA_empty) showed colony formation after one day of incubation at 37 °C. In contrast, *E. coli* cells harboring a deletion of the gene encoding for cytosine deaminase (Δ*codA*) did not show any colony formation after transformation with pTNA_empty and incubation at 37 °C for ten days. Transformation of the same Δ*codA*-knockout strain with pTNA_*codA*, which ensures low constitutive expression of the *codA* gene by a tryptophanase promoter[14], resulted in colony formation after two days of incubation at 37 °C. Transformation of the Δ*codA*-knockout strain with pTNA_*atzC* did not show any colony formation after ten days of incubation at 37 °C. Transformation of the Δ*codA*-knockout strain with pTNA_*atzC_D188H_H219E* resulted in colony formation after four days of incubation at 37 °C.

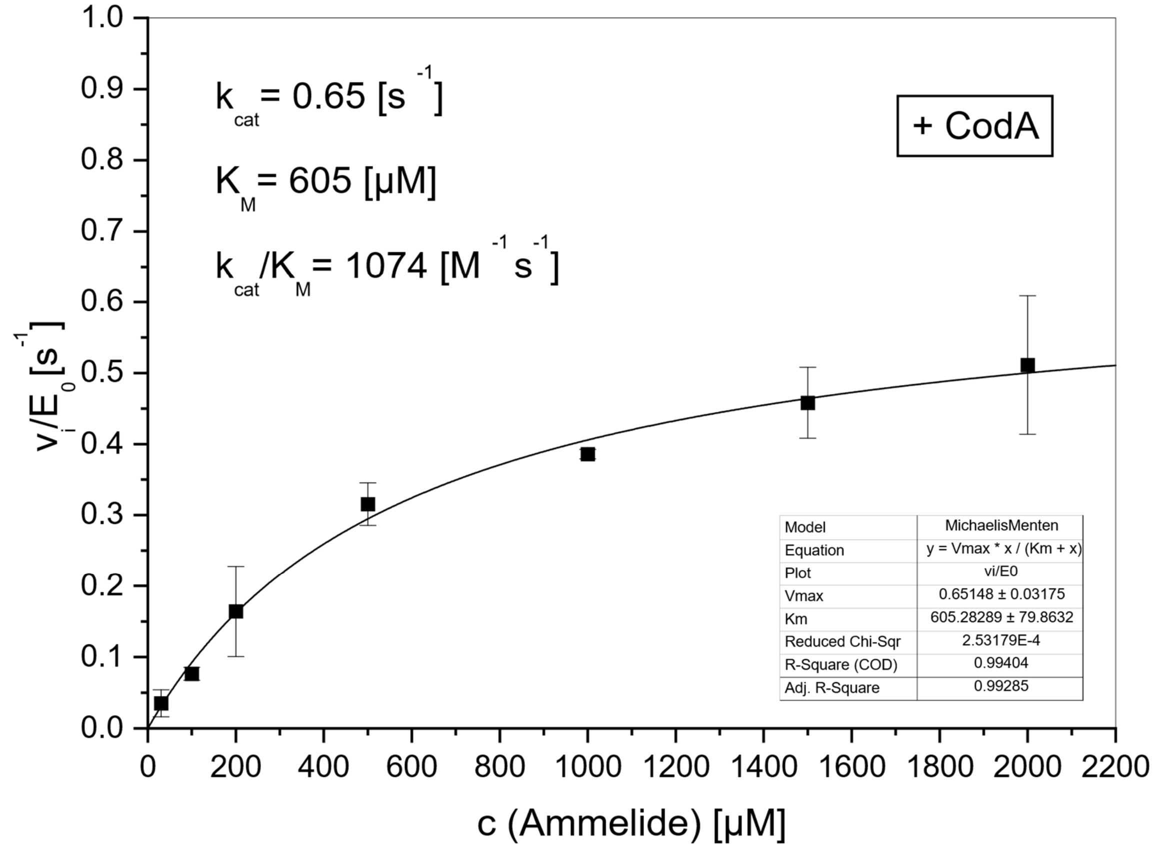

Figure S21: Steady-state enzyme kinetics of CodA with ammelide 14 as substrate. Assay conditions were 50 mM KP buffer (pH 7.5), 2 µM CodA, a variable concentration of ammelide ranging from 30 - 2000 µM, 25 °C. Triplicate measurements were performed at 230 nm using a differential molar extinction coefficient between ammelide and cyanuric acid of 7030 M^{-1} cm^{-1}. Determined initial velocities v_i were normalized to the total enzyme concentration E_0 and plotted against the substrate concentration. The data was fitted to a hyperbolic saturation curve with Origin (2022). A remarkably high promiscuous activity of 1074 M^{-1} s^{-1} for the xenobiotic compound ammelide **14** was found, which is similar to the promiscuous activity of some guanine deaminases for the xenobiotic substance ammeline **4** as identified in previous studies.[18,25]

Table S1: Computational substrate interaction analysis.

	E. coli **GuaD**	*E. coli* **CodA**
N^2,N^2-Dimethylguanine **1**	y	n
Guanine **2**	y	n
Hydroxyatrazine **3**	y	n
Ammeline **4**	y	n
8-Oxoguanine **5**	y	n
CAOT **6**	y	n
Isocytosine **9a**	y	n
Cytosine **7**	n	y
Isoguanine **8**	n	y
Isocytosine **9b**	n	y
5-Azacytosine **10**	n	y
N^4-Methylcytosine **11**	n	y
Creatinine **12**	n	y
N-Ethylammelide **13**	n	y
Ammelide **14**	n	y
Cyanuric monochloride **15**	n	y
N-Isopropylammelide **16**	n	y

The interactions of the identified substrates were analyzed in complex with *E. coli* GuaD/CodA. If an interaction between the exocyclic oxygen of the 6/4-π-electron system and the respective active site glutamine was observed (**y**), we considered the complex as catalytically active. If no such interaction was observed (**n**), we considered the complex as inactive. To obtain structural models as input for the interaction analysis, all in this study experimentally identified substrates were superimposed with the native substrate in our pre-reaction state I structural models of guanine deaminase (**Figure S7A**) and cytosine deaminase (**Figure S8A**). Details of the modeling and interaction analyses are available in the Materials and Methods section. This analysis of the substrate interaction with the active site glutamine fully agrees with the observed experimental results as shown in **Figure 3**.

Table S2: Catalytic efficiencies for the hydrolysis of N^2,N^2-dimethylguanine 1, guanine 2, and hydroxyatrazine 3 by AtzB wildtype and variants.

Protein	k_{cat}/K_M [M^{-1} s^{-1}] (N^2,N^2-dimethylguanine **1**)	k_{cat}/K_M [M^{-1} s^{-1}] (guanine **2**)[a]	k_{cat}/K_M [M^{-1} s^{-1}] (hydroxyatrazine **3**)[a]
**AtzB wildtype**	820±140	n.d.	240000±120000
**I170N**	940±240	n.d.	41000±6900
**S218C**	2200±590	9.8±0.23	52000±10000
**S219Q**	1200±190	n.d.	38000±9100
**I222N**	210±60	n.d.	10000±1600
**S218C I170N**	2600±680	13±0.66	15000±3700
**S218C S219Q**	10000±2300	43±1.3	18000±8100
**S218C I222N**	300±80	2.9±0.10	7100±1600
**S219Q I170N**	2500±430	n.d.	1300±170
**I170N I222N**	1700±270	7.0±0.34	6700±1200
**S219Q I222N**	2900±990	47±14	630±100
**S218C S219Q I170N**	11000±1200	61±2.3	4800±1600
**S218C S219Q I222N**	1800±470	n.d.	7400±1400
**S218C I170N I222N**	24000±3800	250±8.3	4400±520
**S219Q I170N I222N**	11000±1800	45±1.3	11
**S218C S219Q I222N I170N**	240000±28000	3900±640	290±33

The shown k_{cat}/K_M values were determined by steady-state kinetic experiments at 25°C. Mean values and standard deviations are derived from triplicate measurements. Previously, we showed that the four listed active site mutations lead to a stepwise change of the specificity of AtzB from **3** to **2**.[1] Here, we have tested the effect of the same four mutations for the hydrolysis of **1**. The results show that the four mutations also lead to a stepwise change of the specificity of AtzB from **3** to **1.** Taken together, only few mutations in the AtzB enzyme are required to efficiently explore the substrate specificity space of **1**, **2**, and **3** (**Figure S15**).

[a]Values were taken from our previous study.[1]

Table S3: Characteristics and amino acid sequences of the analyzed AHS enzymes.

Protein	MW [Da]	ε_{280} [$M^{-1}cm^{-1}$]	Number of amino acids	Sequence
**GuaD (*Escherichia coli*)**	51309	76780	447	MMSGEHTLKAVRGSFIDVTRTIDNPEEIASA<br>LRFIEDGLLLIKQGKVEWFGEWENGKHQI<br>PDTIRVRDYRGKLIVPGFVDTHIHYPQSEMV<br>GAYGEQLLEWLNKHTFPTERRYEDLEYAR<br>EMSAFFIKQLLRNGTTTALVFGTVHPQSVD<br>ALFEAASHINMRMIAGKVMMDRNAPDYLLD<br>TAESSYHQSKELIERWHKNGRLLYAITPRFA<br>PTSSPEQMAMAQRLKEEYPDTWVHTHLCE<br>NKDEIAWVKSLYPDHDGYLDVYHQYGLTGK<br>NCVFAHCVHLEEKEWDRLSETKSSIAFCPT<br>SNLYLGSGLFNLKKAWQKKVKVGMGTDIGA<br>GTTFNMLQTLNEAYKVLQLQGYRLSAYEAF<br>YLATLGGAKSLGLDDLIGNFLPGKEADFVV<br>MEPTATPLQQLRYDNSVSLVDKLFVMMTLG<br>DDRSIYRTYVDGRLVYERNLEHHHHHH
**8-OxoGuaD (*Pseudomonas aeruginosa*)**	49368	54430	457	MSRTWIRNPLAIFTANGLDAAGGLVVEDGRI<br>VELLGAGQQPAQPCASQFDASRHVVLPGL<br>VNTHHHFYQTLTRAWAPVVNQPLFPWLKTL<br>YPVWARLTPEKLELATKVALAELLLSGCTT<br>AADHHYLFPGGLEQAIDVQAGVVEELGMRA<br>MLTRGSMSLGEKDGGLPPQQTVQEAETILA<br>DSERLIARYHQRGDGARVQIALAPCSPFSV<br>TPEIMRASAEVAARHDVRLHTHLAETLDEE<br>DFCLQRFGLRTVDYLDSVGWLGPRTWLAH<br>GIHFNAEEIRRLGEAGTGICHCPSSNMRLAS<br>GICPTVELEAAGAPIGLGVDGSASNDASNMI<br>LEARQALYLQRLRYGAERITPELALGWAT<br>RGSARLLGRSDIGELAPGKQADLALFKLDEL<br>RFSGSHDPLSALLLCAADRADRVMVGGAW<br>RVVDGAVEGLDLAALIARHRAAASALIAGLE<br>HHHHHH
**WP_135441580 (*Haliea sp.* SAOS-164)**	47464	46870	438	MHHHHHHLDMGSGILRGRILHCIDTPGADG<br>AGVEYLEDGVLQFEDGVITLLADAREASAG<br>GLQLGDVPHLGAGLIVPGFIDTHVHAPQLAI<br>LGSYGEQLMAWLERYTFPAEARFADPDYA<br>AAAMDEFLGEMLRHGTTSALVFSTSHEDAT<br>EALFTAARARDLRLVAGKVLMDRNAPQGLL<br>DTAASGEAASRRLIERWHGAGRLAYAVTPR<br>FSITCSDEQLAAAGRLLRDYPGVYLQTHLAE<br>NPGEIAAVAELFPDAAHYLDTYDRHGLCGE<br>RSFFAHCVHLQPDELERLAATDSRVSLCPS<br>SNMFLGSGLYDWAQLEREGVCISLGSDVG<br>AGTSLSMLRTLGDAYRVCQLQEMSLPPMQ<br>GLYAVTLGNARALGVAERIGNLAVGSEADF<br>LVLDPAGNPQVQRRLQDVSDIDEEWFVYM<br>MLGDERLVASTWVAGREVVR

**WP_026789444 (*Pleomorphomonas oryzae*)**	49753	31860	455	MHHHHHHLDMTFSDRFPSDRTLVRGRVLSFKRRPQRAGDTDAYTYLEDGVIVIDAGKVTDVIDASEIGRVGGKGVVLHDFSGKLILPGFIDTHIHFPQTQVIASYGEQLLEWLTRYTFPAESRYGDPAFAAAQARFFIDELLRNGTTTAVCYGSVHKGAAEALLTESERRGTAMFVGKTAMDRNAPPDVLDTAQSAYDDTASLITAWHGRGRQKVVITPRFAITSTPEQLEALGNLARAHPDCLVQTHLSENLEEIATVERLFPERSDYLDVYDHYGLVGPKSLMGHAIHLTPREIVRMSESGAVAVFCPTSNLFIGSGLFDYKGLEGEPYPVRIALATDVGGGTSYSMLATAAEAYKVMQLRGQKLSAIEAFHLMTRGNAEALGEPDLGRIEPGAHADLVVLDSTARPAMAHRLAAGNCDLEEELFVLMTLGGEQNVQEVFIGGQPQGLHRADE
**AtzB (*Pseudomonas sp.* strain ADP)**	53179	49390	489	MTTTLYTGFHQLVTGDVAGTVLNGVDILVRDGEIIGLGPDLPRTLAPIGVGQEQGVEVVNCRGLTAYPGLINTHHHFFQAFVRNLAPLDWTQLDVLAWLRKIYPVFALVDEDCIYHSTVVSMAELIKHGCTTAFDHQYNYSRRGGPFLVDRQFDAANLLGLRFHAGRGCITLPMAEGSTIPDAMRESTDTFLADCERLVSRFHDPRPFAMQRVVVAPSSPVIAYPETFVESARLARHLGVSLHTHLGEGETPAMVARFGERSLDWCENRGFVGPDVWLAHGWEFTAADIARLAATGTGVAHCPAPVFLVGAEVTDIPAMAAAGVRVGFGVDGHASNDSSNLAECIRLAYLLQCLKASERQHPVPAPYDFLRMATQGGADCLNRPDLGALAVGRAADFFAVDLNRIEYIGANHDPRSLPAKVGFSGPVDMTVINGKVVWRNGEFPGLDEMELARAADGVFRRVIYGDPLVAALRRGTGVTPCLEHHHHHH
**AtzB_Hom_Hal (*Haliea sp.* SAOS-164)**	50682	43430	473	MSTVLFRNFRQLVCAGAPGSVLRDVDLCARDGMITAIGPQLPLTDVDEVVDCGGLTAYPGLVNTHHHFFQALVRNLPGLDWTTLSLLEWLDTIYPIFARLDEDCIYHASLISLADLLKHGCTTAFDHQYNFNSNMGSRVVDRQFEAAALLGARLHVGRGCNTLPMSAGSTIPDAMLETTDAFLADCERLIGAFHNPAPGAMAQVVVAPCQPVNSLPETFPEAAALARRHGVRLHTHLSEGENAAMLDRFGMRSLDWCESVGFVGPDVWFAHGWEFTPPEIARLAATGTGVAHCPAPVFLVGAEVTDLPAMVAADMTVGMGVDGQASNDSSNLAECMRLAYLLQCLNARHNPLPAPPPERYLHMATAGGAACLGRTDIGELAVGKAADFFCADLNGLDYAGADSDPLSLPAKVGFAGPAAMTVVHGRVVWRDGEFPGLDETQLRSAADALLREKLDGHLAPLRTPGLEHHHHHH

**AtzB_Hom_Pleo (*Pleomorphomonas oryzae*)**	51181	48360	467	MGNYLLKNCAAVMVDDGAGLNARRNVDILTDGPAIKAIEPHLAETPQSVGAEVIDASGWFVYPGLVNTHHHFFQTFVRNRAELDWTKLSVLEWLDRIYPIFSQLTEDCFYHSSLTAMAELIKHGCTTALDHQYCFPRHAGKYLVDRQFEAAERLGIRYHAGRGGNTLPKSEGSTIPDAMLETTDEFLADCERLIDRYHDASPFSLRQVVISPCQPVNSYRETFVESVALARDKGVFLHTHVGEGESPVMEARHGKRTVDYLEEMGFAGPDVFYAHCWELTHTELAKLAASGTGVSHCPEPVYLVGAEVTDIPAMAALGVRVGLGCDGSASNDNSNLMHCIHSAYMLQCLVASSRSHPVPAPAEFLRFATTGSASLLGRADIGRLAPGMAADLFAIDTRRMDYVGTRHDPLSLPAKLGIGMATDLTMINGRIVWANGEFPGIDEAEMAAEAEATLATIDFLEHHHHHH
**MBD1203459 (*Rhodobacteraceae bacterium*)**	50303	46870	462	MHHHHHHLDMTAHLFKGCAAVICDPHSVLRDVDLLVEGPKIAAIGKGLVAPTGAEVIDARGWFLYPGLVNTHHHFFQTFVRNRADLDWTKLSVIEWLDLIYPIFSRLTEDCFYHSSLTAMAELAKHGCTTAFDHQYNYPRHAGKRLVDRQFEAAAKIGLRFHAGRGGNTLPKSQGSTIPDEMLESTDEFIADCARLIDTYHDSAPFSMAQVVVSPCQPVNCYRETFVESAALARDKGVFLHTHVGEGESQVIAARHGMRTVDYLEQIGFAGPDTFYAHCWELTDTELRQLAASGTGVAHCPEPVYLVGAEVTDVPAMAAFGVRLGLGCDGSASSDNSNLMHCIHSGYMLQCLVASRRAHPVPEPRDFLGYATAGGAALLGRSDIGRLAPGMAADLFAIDTRRMDYVGTRHDPASLIAKVGIAMPTDLTMVNGRIVWAKGEFPGLDEAQMAAEAEAVLATINA
**CodA (*Escherichia coli*)**	48656	56380	435	MSNNALQTIINARLPGEEGLWQIHLQDGKISAIDAQSGVMPITENSLDAEQGLVIPPFVEPHIHLDTTQTAGQPNWNQSGTLFEGIERWAERKALLTHDDVKQRAWQTLKWQIANGIQHVRTHVDVSDATLTALKAMLEVKQEVAPWIDLQIVAFPQEGILSYPNGEALLEEALRLGADVVGAIPHFEFTREYGVESLHKTFALAQKYDRLIDVHCDEIDDEQSRFVETVAALAHHEGMGARVTASHTTAMHSYNGAYTSRLFRLLKMSGINFVANPLVNIHLQGRFDTYPKRRGITRVKEMLESGINVCFGHDDVFDPWYPLGTANMLQVLHMGLHVCQLMGYGQINDGLNLITHHSARTLNLQDYGIAAGNSANLIILPAENGFDALRRQVPVRYSVRGGKVIASTQPAQTTVYLEQPEAIDYKRLEHHHHHH

**TrzC (*Paracidovorax citrulli*)**	45002	4470	420	MSMETHSYVDVAIRNARLADTEGIVDILIHD<br>GRIASIVKSTKTKGSVEIDAHEGLVTSGL<br>VEPHIHLDKALTADRVPAGSIGDLRTRRGLE<br>MAIRATRDIKRTFTVEDVRERAIRAALMA<br>SRAGTTALRTHVDVDPIVGLAGIRGVLEARE<br>VCAGLIDIQIVAFPQEGLFCSAGAVDLMR<br>EAIKLGADAVGGAPALDDRPQDHVRAVFDL<br>AAEFGLPVDMHVDESDRREDFTLPFVIEAA<br>RERRVPNVTVAHISSLSVQTDDVARSTIAAL<br>ADADVNVVVNPIIVKITRLSELLDAGVSV<br>MFGSDNLRDPFYPLGAANPLGSAIFACQIAA<br>LGTPQDLRRVFDAVTINAARMLGFPSLLG<br>VVEGAVADLAVFPSATPEEVVLDQQSPLFV<br>LKGGRVVAMRLAAGSTSFRDYSLEHHHHH<br>H
**AtzC (*Pseudomonas sp.* strain ADP)**	46004	50880	411	MSKDFDLIIRNAYLSEKDSVYDIGIVGDRIIKI<br>EAKIEGTVKDEIDAKGNLVSPGFVDAH<br>THMDKSFTSTGERLPKFWSRPYTRDAAIED<br>GLKYYKNATHEEIKRHVIEHAHMQVLHGTL<br>YTRTHVDVDSVAKTKAVEAVLEAKEELKDLI<br>DIQVVAFAQSGFFVDLESESLIRKSLDMG<br>CDLVGGVDPATRENNVEGSLDLCFKLAKEY<br>DVDIDYHIHDIGTVGVYSINRLAQKTIENG<br>YKGRVTTSHAWCFADAPSEWLDEAIPLYKD<br>SGMKFVTCFSSTPPTMPVIKLLEAGINLGC<br>ASDNIRDFWVPFGNGDMVQGALIETQRLEL<br>KTNRDLGLIWKMITSEGARVLGIEKNYGIE<br>VGKKADLVVLNSLSPQWAIIDQAKRLCVIKN<br>GRIIVKDEVIVALEHHHHHH
**MBE3094533 (*Actinobacteria bacterium*)**	48598	44350	430	MIMDFDLIIRQAYVRKQDNILDIGIVKDRITRI<br>SEHIEEKAKEEINANGKFLSPGFVNSHVHM<br>DKSMTSIGERFPKYNNDSRDEDNALRDVN<br>RRKRIEAGLKYYSTASIEEVKSNTIQHAYSSI<br>ENGTSFIRTFVDIDKVARLKALEGVLAARNE<br>LQELIDIQVVAFAQSGFLADPESEPLVRKAIE<br>MGADLVGSLNPVNPVTCEGNIEKALDLVFKI<br>AKDYNVDIDNHNMDIGTLGIYTLEAQAKKAI<br>ENDYIGRVTASHFYALGDAPIAWIDRAIPKF<br>KEAGMKFVTCYLSTPYEMPVKKLLLAGITIAI<br>ATDNVRDFWRAYGNTDLVQAVLIEIHKLKM<br>TTNPDLDLLWDMITTEGAKVLGIEEDYGIEE<br>GKKADLVLLDALSPQWAIVDQAKKLYVIKNG<br>KVIVRNEEILPEFKKYEVLEHHHHHH

**WP_013837184 (*Novosphingobium sp.* PP1Y)**	43233	34950	406	MDNWLINGRLADGSPLAIGIADGRIDHLCAAAPDVVEGEVHDLEGRLVVPPFIDGHIHLDKSFLPGGWQPHRACTGAFDVRERVAFEKAALANARPVRERAMALAELALGNGTLHLRSHADVDMKAGLSNVEALLDLREAMADAITVQIVAFPQSGILSDPGTADLLSAAVDAGADLVGGIDPVGFDGAQDDHLDIVFGIADRKGVPIDIHLHDFGMVGIAQLEDIAQRTQALGMGGRVTVSHAYALGDVPLDVARRTGALLARAGVAIMTNGPGPQPCPPVAALAQEGVLLFSGSDNVRDAWWPYGDADMLERAMMVGYRAGLYTDKELELAFAMVTGNAARALGLRDYGLAEGMPADFVVLNAQSVAEAVVARPKRREVWRGGRRIAVEEKKIDAELEHHHHHH
**HBY46137 (*Chloroflexi bacterium*)**	42145	26930	399	MNDLILRNATLPGGETATIAIAGQRIAAIDRTGHSPVSGSAELDLRGALVLPGLVDGHIHLDKTYLGDGWHSHRPSTSIVAHVAAERQERHALAPVEQRASALIERAITNGTTILRTHADIDPVSGLSNLDGILAARERYRDAITIQVVAFPQSGIIAAPGTYDLLDEALRCGADLIGGLDPAGFDNDVDGHLEAIFGLADRHSVGVDIHLHDAGDVGLAELQQIAERAVALGMQGRVTVGHAYALGTEPWRRVAPVAEALASAGVSIMTTAPGSHAFPPVLALRAAGVNVFAANDNIRDSWSPFGEADMLERMMLVAYRSGFTTDAELDVAFDLGTNAAARALGIEGYGLAVGNPADLVVLDARHVAEAVVARPPRRYVMRHGVIVAQDGLEHHHHHH

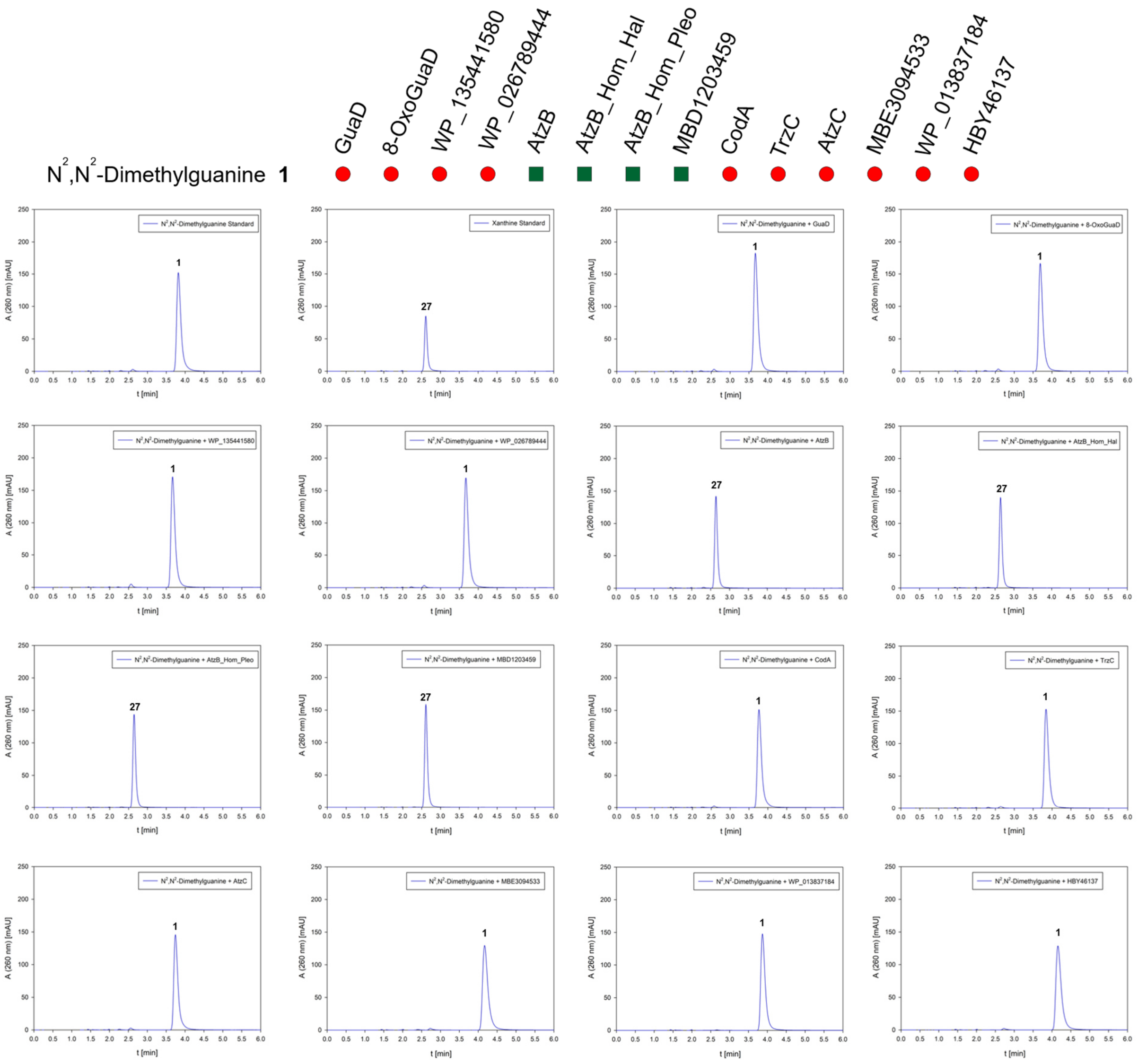

Source Data 1: HPLC-based analysis of the turnover of N^2,N^2-dimethylguanine 1 to xanthine 27 by different AHS enzymes. Substance standards as well as reaction mixtures of 500 µM **1**, 50 mM KP pH 7.5, and 2 µM of the tested enzyme were incubated at 25 °C and 500 rpm for 24 h. After centrifugation with a filter tube, the reaction products were analyzed via reversed-phase HPLC. Shown are the respective HPLC chromatograms at 260 nm. All substances were unambiguously identified using the retention times as well as the spectra of each peak. Dark green squares indicate enzyme-substrate pairs for which product formation was detected that has not been previously reported. Red circles indicate enzyme-substrate pairs for which no product formation was detected.

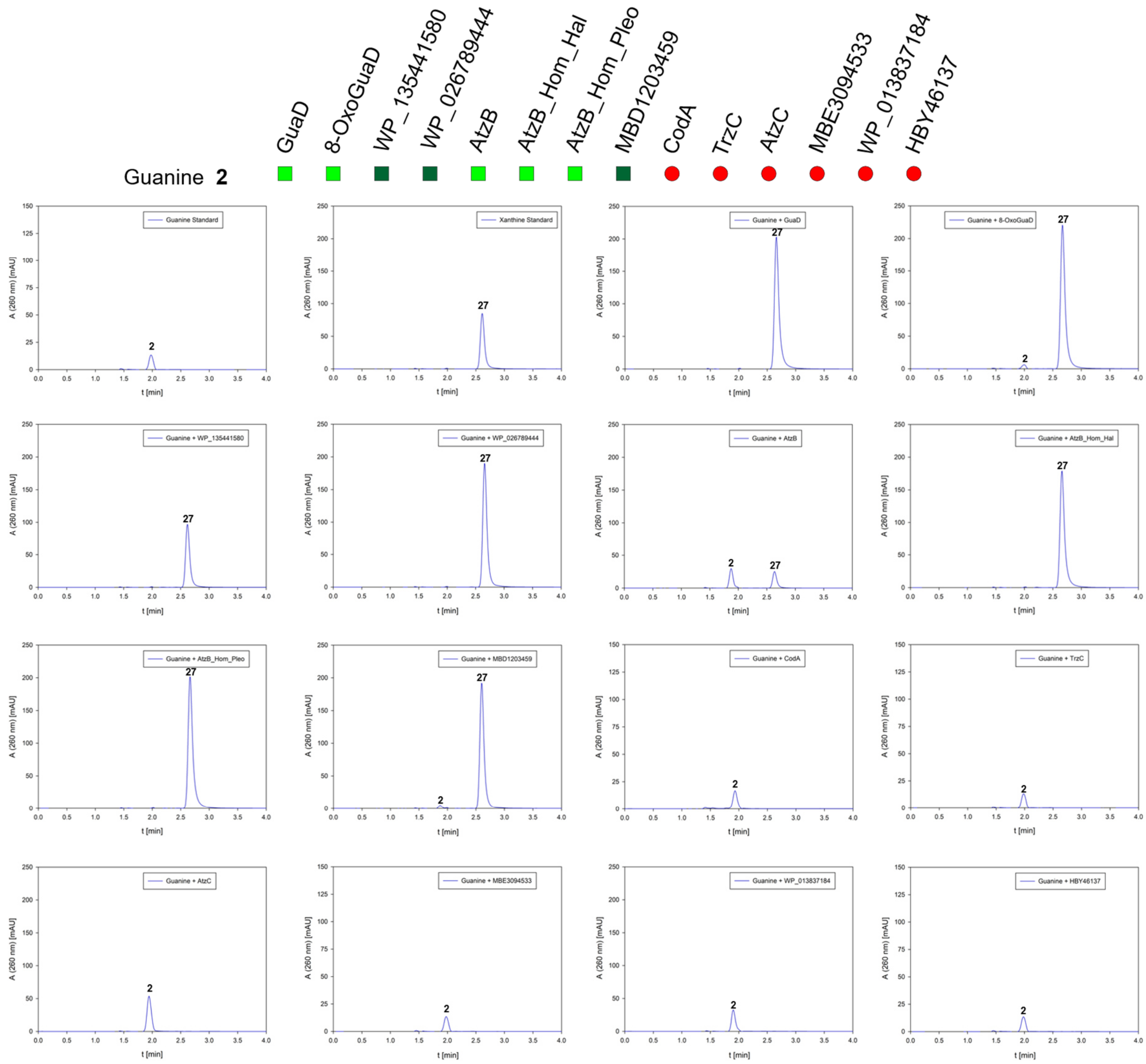

Source Data 2: HPLC-based analysis of the turnover of guanine 2 to xanthine 27 by different AHS enzymes. Substance standards as well as reaction mixtures of 500 µM **2**, 50 mM KP pH 7.5, and 2 µM of the tested enzyme were incubated at 25 °C and 500 rpm for 24 h. After centrifugation with a filter tube, the reaction products were analyzed via reversed-phase HPLC. Shown are the respective HPLC chromatograms at 260 nm. All substances were unambiguously identified using the retention times as well as the spectra of each peak. Dark green squares indicate enzyme-substrate pairs for which product formation was detected that has not been previously reported. Light green squares indicate enzyme-substrate pairs for which product formation was detected, confirming data from the literature. Red circles indicate enzyme-substrate pairs for which no product formation was detected.

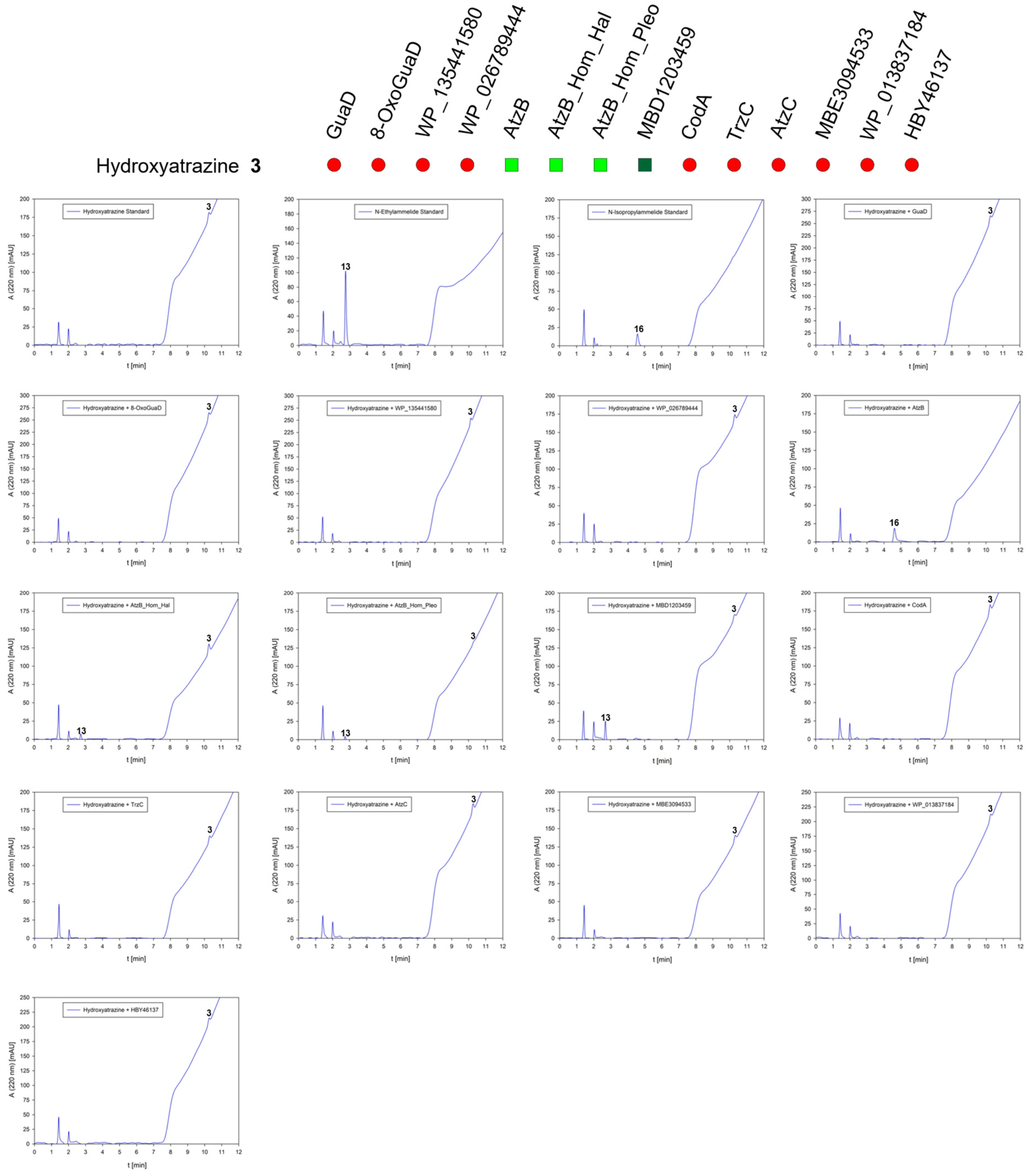

Source Data 3: HPLC-based analysis of the turnover of hydroxyatrazine 3 to N-ethylammelide 13 or N-isopropylammelide 16 by different AHS enzymes. Substance standards as well as reaction mixtures of 500 µM **3**, 50 mM KP pH 7.5, and 2 µM of the tested enzyme were incubated at 25 °C and 500 rpm for 24 h. After centrifugation with a filter tube, the reaction products were analyzed via reversed-phase HPLC. Shown are the respective HPLC chromatograms at 220 nm. All substances were unambiguously identified using the retention times as well as the spectra of each peak. Dark green squares indicate enzyme-substrate pairs for which product formation was detected that has not been previously reported. Light green squares indicate enzyme-substrate pairs for which product formation was detected, confirming data from the literature. Red circles indicate enzyme-substrate pairs for which no product formation was detected.

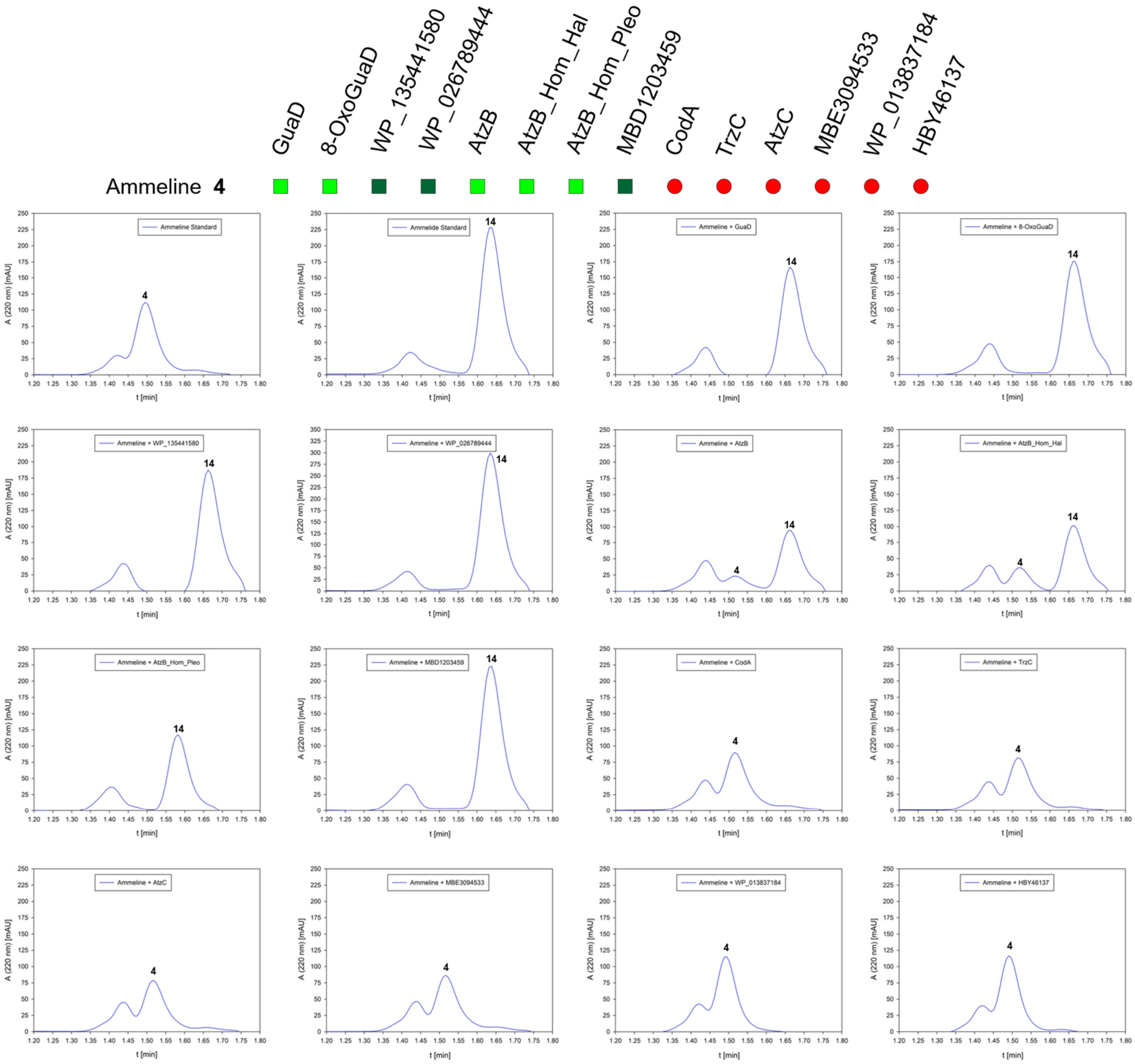

Source Data 4: HPLC-based analysis of the turnover of ammeline 4 to ammelide 14 by different AHS enzymes. Substance standards as well as reaction mixtures of 500 µM **4**, 50 mM KP pH 7.5, and 2 µM of the tested enzyme were incubated at 25 °C and 500 rpm for 24 h. After centrifugation with a filter tube, the reaction products were analyzed via reversed-phase HPLC. Shown are the respective HPLC chromatograms at 220 nm. All substances were unambiguously identified using the retention times as well as the spectra of each peak. Dark green squares indicate enzyme-substrate pairs for which product formation was detected that has not been previously reported. Light green squares indicate enzyme-substrate pairs for which product formation was detected, confirming data from the literature. Red circles indicate enzyme-substrate pairs for which no product formation was detected.

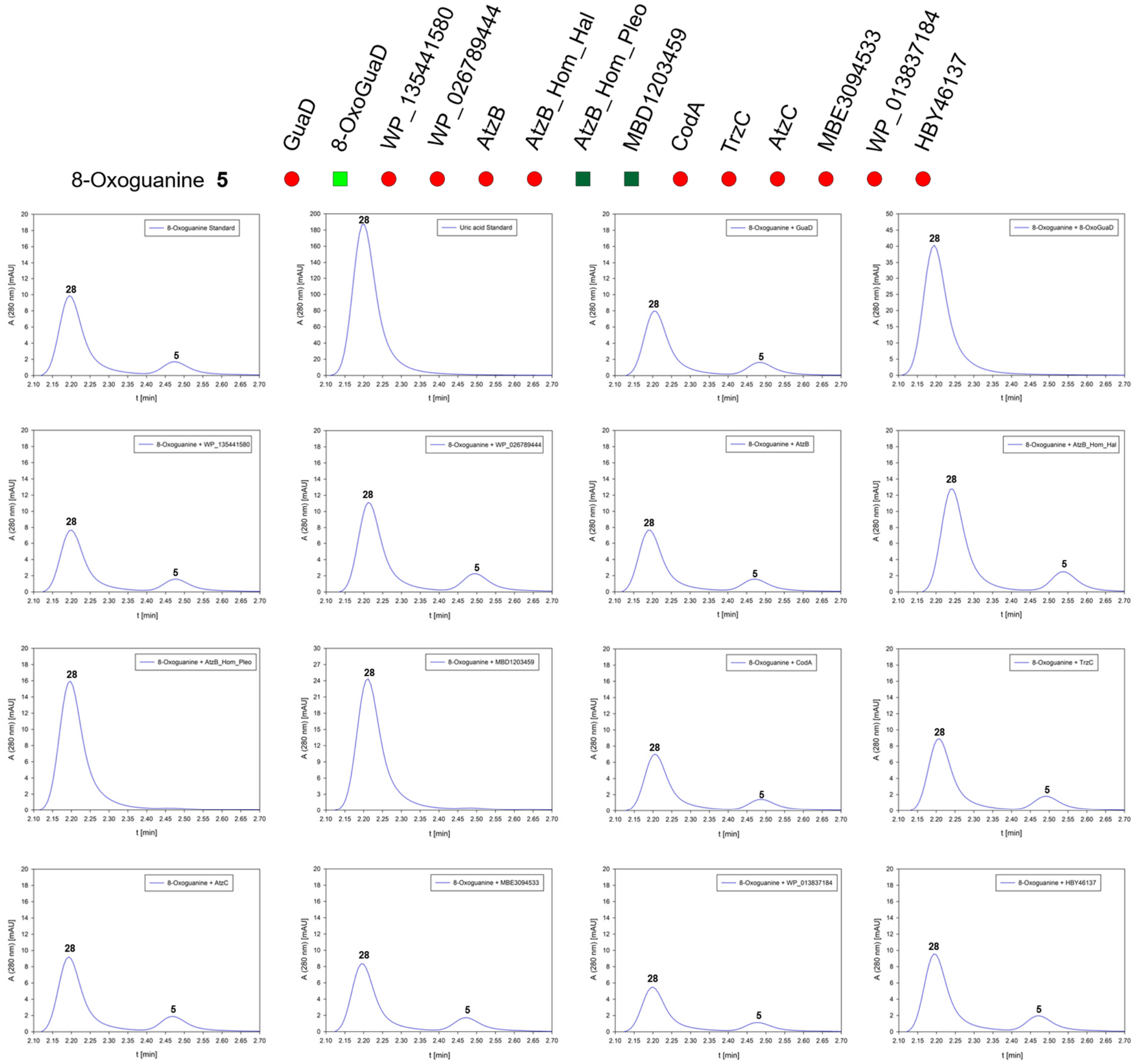

Source Data 5: HPLC-based analysis of the turnover of 8-oxoguanine 5 to uric acid 28 by different AHS enzymes. Substance standards as well as reaction mixtures of 500 µM **5**, 50 mM KP pH 7.5, and 2 µM of the tested enzyme were incubated at 25 °C and 500 rpm for 24 h. After centrifugation with a filter tube, the reaction products were analyzed via reversed-phase HPLC. Shown are the respective HPLC chromatograms at 280 nm. All substances were unambiguously identified using the retention times as well as the spectra of each peak. Due to spontaneous hydrolysis of **5**, a considerable amount of **28** is already formed in the substrate standard. Dark green squares indicate enzyme-substrate pairs for which product formation as well as substrate depletion was detected that has not been previously reported. Light green squares indicate enzyme-substrate pairs for which product formation as well as substrate depletion was detected, confirming data from the literature. Red circles indicate enzyme-substrate pairs for which the substrate:product ratio did not change.

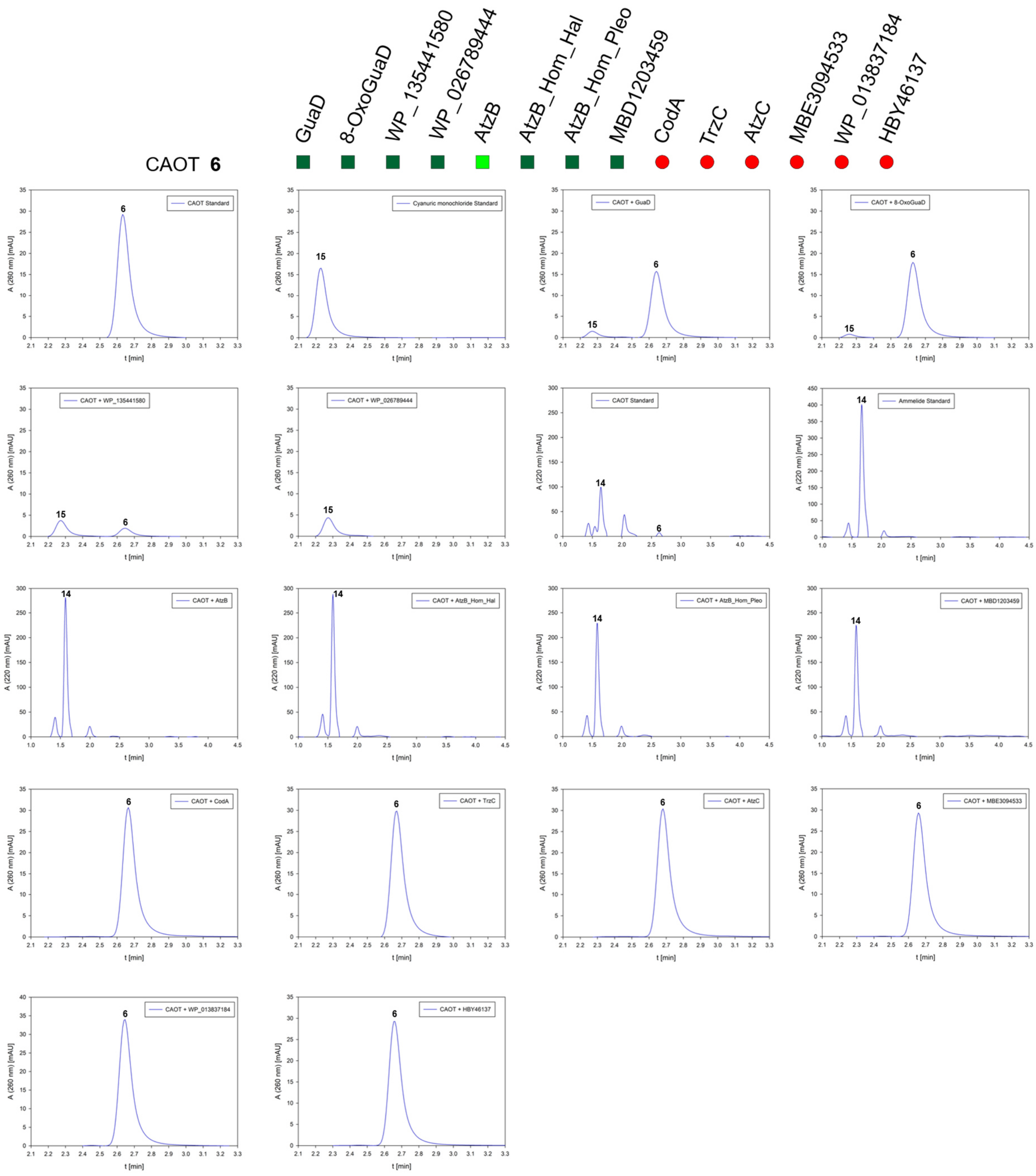

Source Data 6: HPLC-based analysis of the turnover of CAOT (2-chloro-4-amino-6-hydroxy-1,3,5-triazine) 6 to ammelide 14 or cyanuric monochloride 15 by different AHS enzymes. Substance standards as well as reaction mixtures of 500 µM **6**, 50 mM KP pH 7.5, and 2 µM of the tested enzyme were incubated at 25 °C and 500 rpm for 24 h. After centrifugation with a filter tube, the reaction products were analyzed via reversed-phase HPLC. Shown are the respective HPLC chromatograms at 220 nm (hydrolysis to **14**) and at 260 nm (hydrolysis to **15**). All substances were unambiguously identified using the retention times as well as the spectra of each peak. Dark green squares indicate enzyme-substrate pairs for which product formation was detected that has not been previously reported. Light green squares indicate enzyme-substrate pairs for which product formation was detected, confirming data from the literature. Red circles indicate enzyme-substrate pairs for which no product formation was detected.

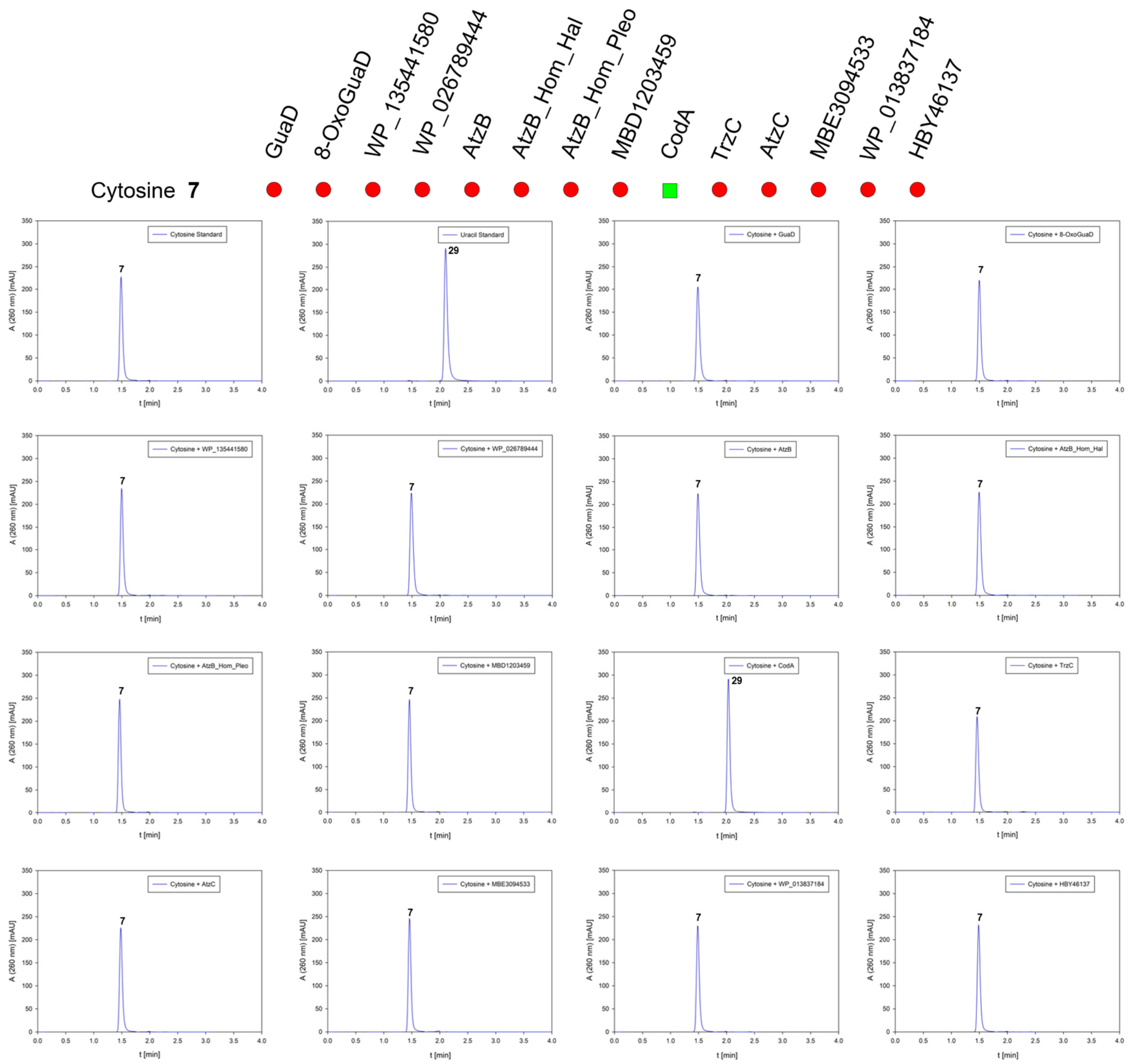

Source Data 7: HPLC-based analysis of the turnover of cytosine 7 to uracil 29 by different AHS enzymes. Substance standards as well as reaction mixtures of 500 µM **7**, 50 mM KP pH 7.5, and 2 µM of the tested enzyme were incubated at 25 °C and 500 rpm for 24 h. After centrifugation with a filter tube, the reaction products were analyzed via reversed-phase HPLC. Shown are the respective HPLC chromatograms at 260 nm. All substances were unambiguously identified using the retention times as well as the spectra of each peak. Light green squares indicate enzyme-substrate pairs for which product formation was detected, confirming data from the literature. Red circles indicate enzyme-substrate pairs for which no product formation was detected.

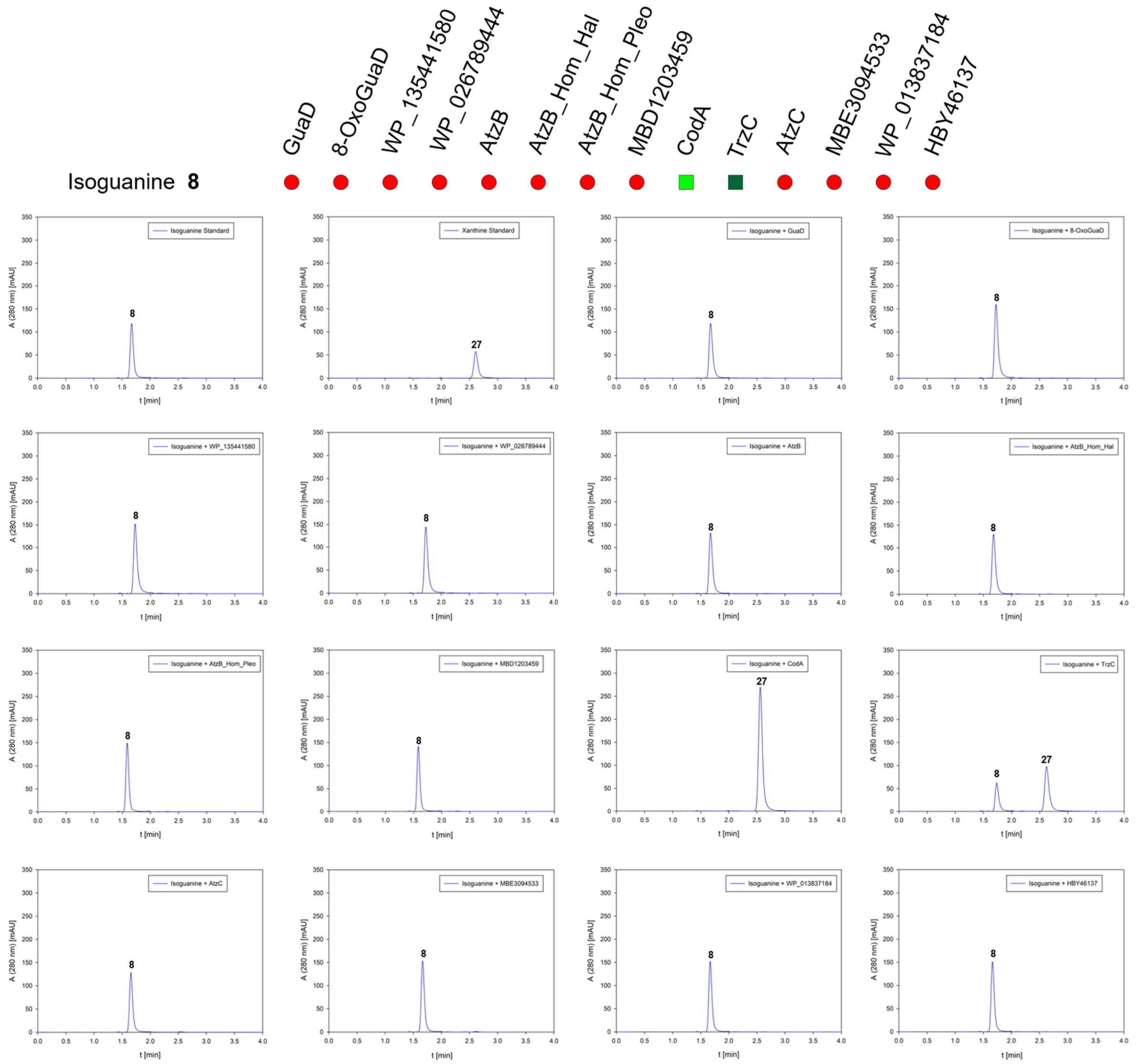

Source Data 8: HPLC-based analysis of the turnover of isoguanine 8 to xanthine 27 by different AHS enzymes. Substance standards as well as reaction mixtures of 500 µM **8**, 50 mM KP pH 7.5, and 2 µM of the tested enzyme were incubated at 25 °C and 500 rpm for 24 h. After centrifugation with a filter tube, the reaction products were analyzed via reversed-phase HPLC. Shown are the respective HPLC chromatograms at 280 nm. All substances were unambiguously identified using the retention times as well as the spectra of each peak. Dark green squares indicate enzyme-substrate pairs for which product formation was detected that has not been previously reported. Light green squares indicate enzyme-substrate pairs for which product formation was detected, confirming data from the literature. Red circles indicate enzyme-substrate pairs for which no product formation was detected.

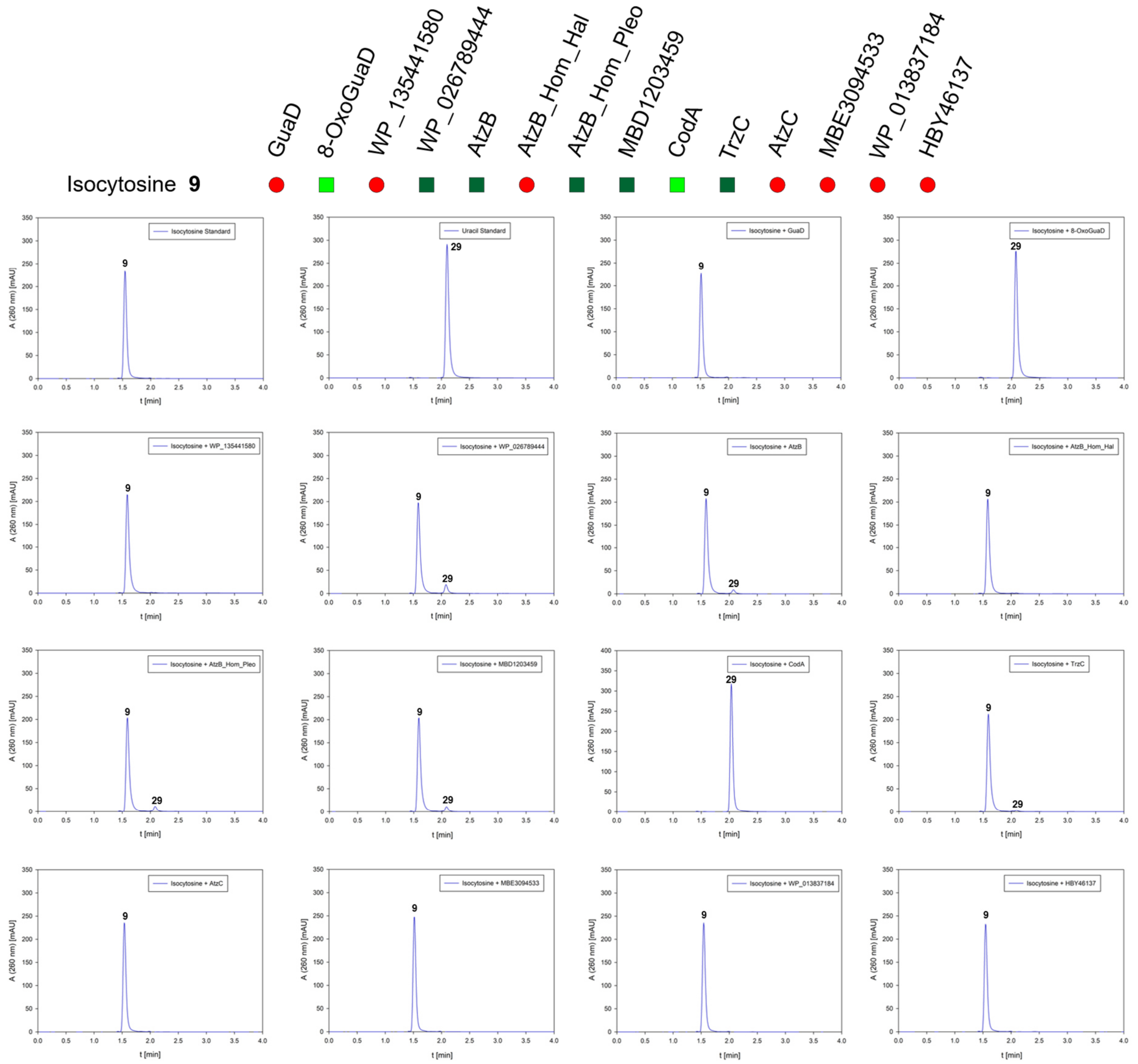

Source Data 9: HPLC-based analysis of the turnover of isocytosine 9 to uracil 29 by different AHS enzymes. Substance standards as well as reaction mixtures of 500 µM **9**, 50 mM KP pH 7.5, and 2 µM of the tested enzyme were incubated at 25 °C and 500 rpm for 24 h. After centrifugation with a filter tube, the reaction products were analyzed via reversed-phase HPLC. Shown are the respective HPLC chromatograms at 260 nm. All substances were unambiguously identified using the retention times as well as the spectra of each peak. Dark green squares indicate enzyme-substrate pairs for which product formation was detected that has not been previously reported. Light green squares indicate enzyme-substrate pairs for which product formation was detected, confirming data from the literature. Red circles indicate enzyme-substrate pairs for which no product formation was detected.

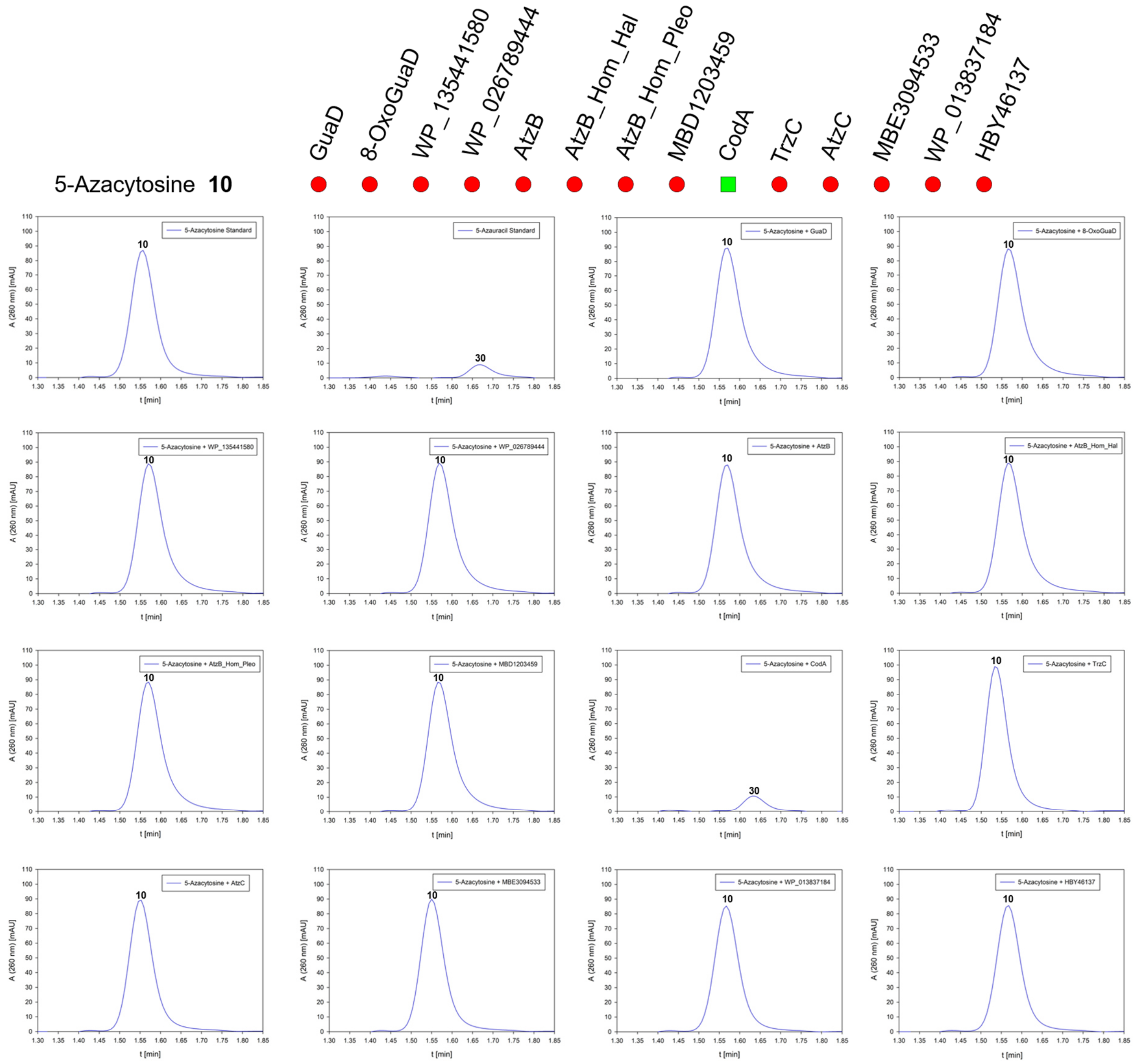

Source Data 10: HPLC-based analysis of the turnover of 5-azacytosine 10 to 5-azauracil 30 by different AHS enzymes. Substance standards as well as reaction mixtures of 500 µM **10**, 50 mM KP pH 7.5, and 2 µM of the tested enzyme were incubated at 25 °C and 500 rpm for 24 h. After centrifugation with a filter tube, the reaction products were analyzed via reversed-phase HPLC. Shown are the respective HPLC chromatograms at 260 nm. All substances were unambiguously identified using the retention times as well as the spectra of each peak. Light green squares indicate enzyme-substrate pairs for which product formation was detected, confirming data from the literature. Red circles indicate enzyme-substrate pairs for which no product formation was detected.

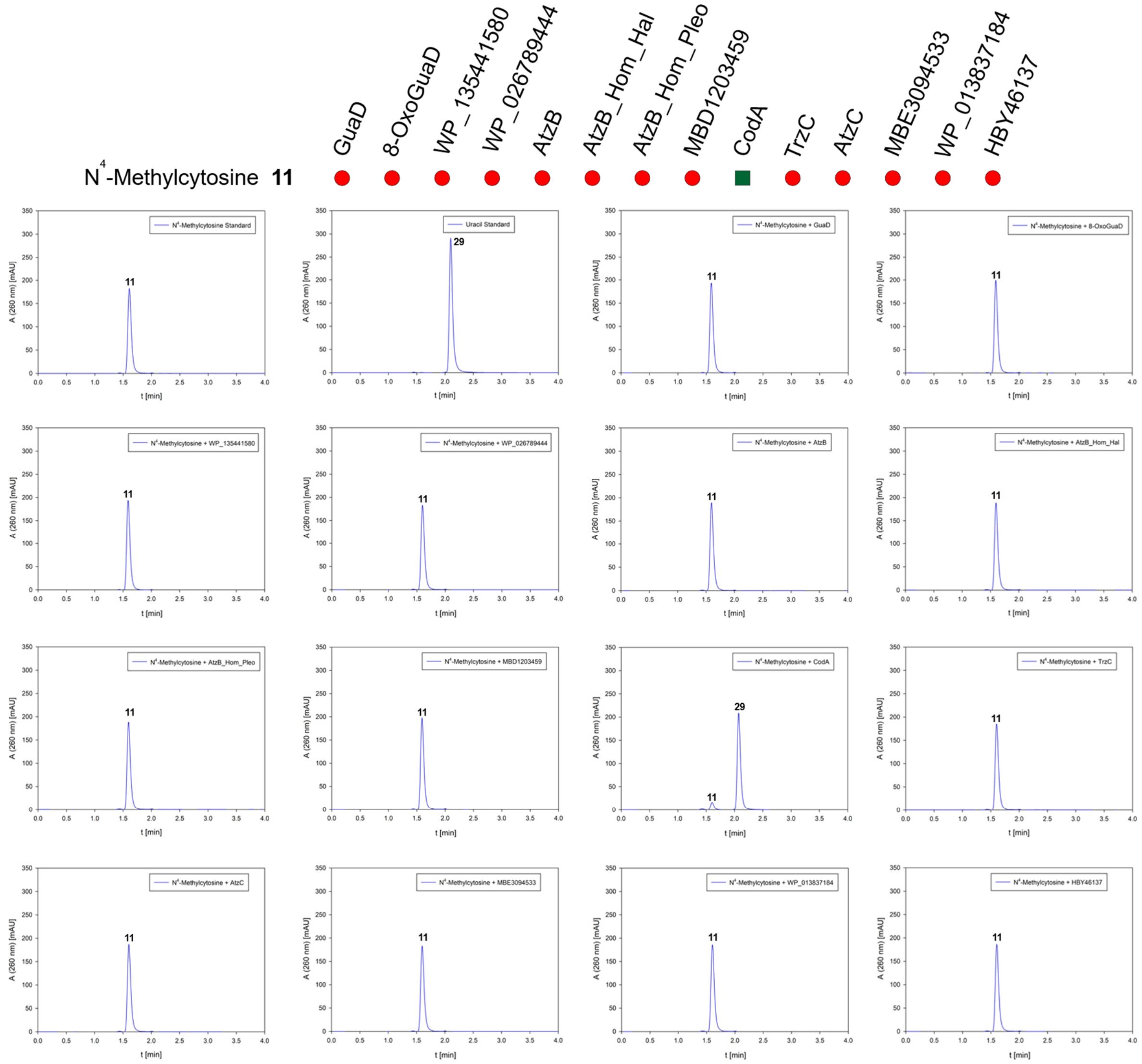

Source Data 11: HPLC-based analysis of the turnover of N^4-methylcytosine 11 to uracil 29 by different AHS enzymes. Substance standards as well as reaction mixtures of 500 µM **11**, 50 mM KP pH 7.5, and 2 µM of the tested enzyme were incubated at 25 °C and 500 rpm for 24 h. After centrifugation with a filter tube, the reaction products were analyzed via reversed-phase HPLC. Shown are the respective HPLC chromatograms at 260 nm. All substances were unambiguously identified using the retention times as well as the spectra of each peak. Dark green squares indicate enzyme-substrate pairs for which product formation was detected that has not been previously reported. Red circles indicate enzyme-substrate pairs for which no product formation was detected.

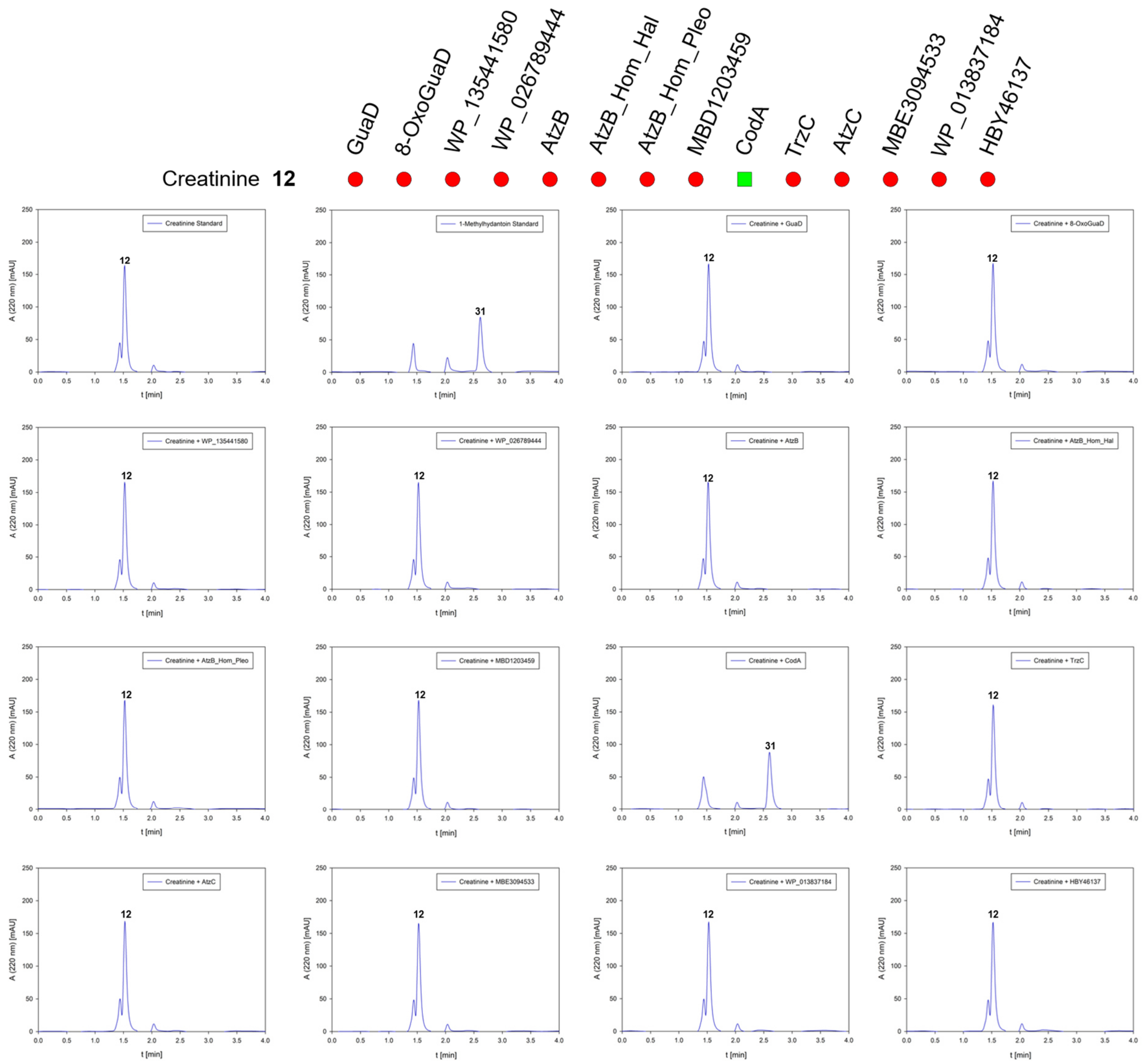

Source Data 12: HPLC-based analysis of the turnover of creatinine 12 to 1-methylhydantoin 31 by different AHS enzymes. Substance standards as well as reaction mixtures of 500 µM **7**, 50 mM KP pH 7.5, and 2 µM of the tested enzyme were incubated at 25 °C and 500 rpm for 24 h. After centrifugation with a filter tube, the reaction products were analyzed via reversed-phase HPLC. Shown are the respective HPLC chromatograms at 220 nm. All substances were unambiguously identified using the retention times as well as the spectra of each peak. Light green squares indicate enzyme-substrate pairs for which product formation was detected, confirming data from the literature. Red circles indicate enzyme-substrate pairs for which no product formation was detected.

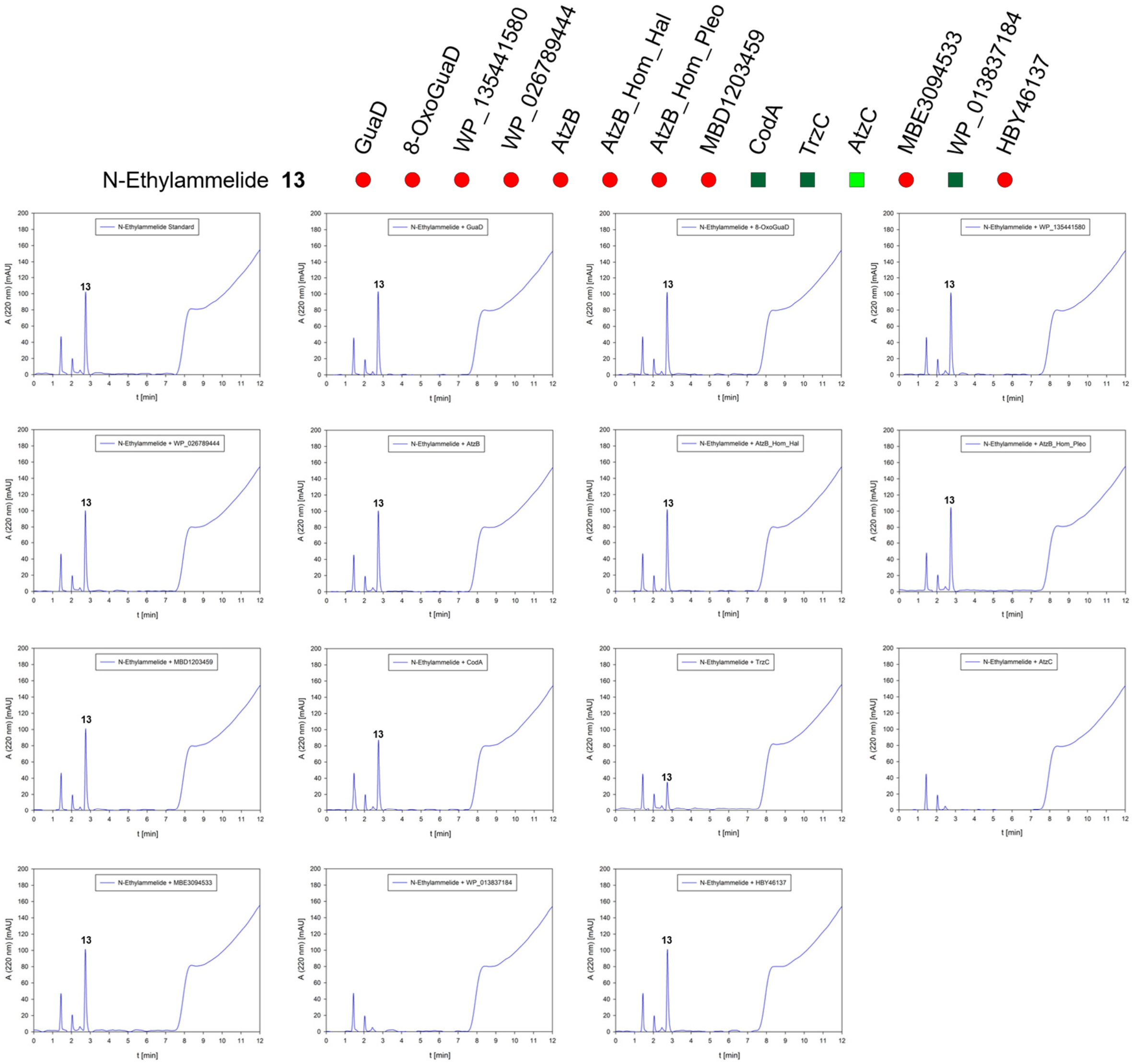

Source Data 13: HPLC-based analysis of the turnover of N-ethylammelide 13 to cyanuric acid 32 by different AHS enzymes. Substance standards as well as reaction mixtures of 500 µM **13**, 50 mM KP pH 7.5, and 2 µM of the tested enzyme were incubated at 25 °C and 500 rpm for 24 h. After centrifugation with a filter tube, the reaction products were analyzed via reversed-phase HPLC. Shown are the respective HPLC chromatograms at 220 nm. All substances were unambiguously identified using the retention times as well as the spectra of each peak. As **32** was neither detectable in its substance standard (not shown) nor in the reaction mixtures, the progress of the reaction was monitored by the decrease of the peak for **13**. Dark green squares indicate enzyme-substrate pairs for which substrate depletion was detected that has not been previously reported. Light green squares indicate enzyme-substrate pairs for which substrate depletion was detected, confirming data from the literature. Red circles indicate enzyme-substrate pairs for which no substrate depletion was detected.

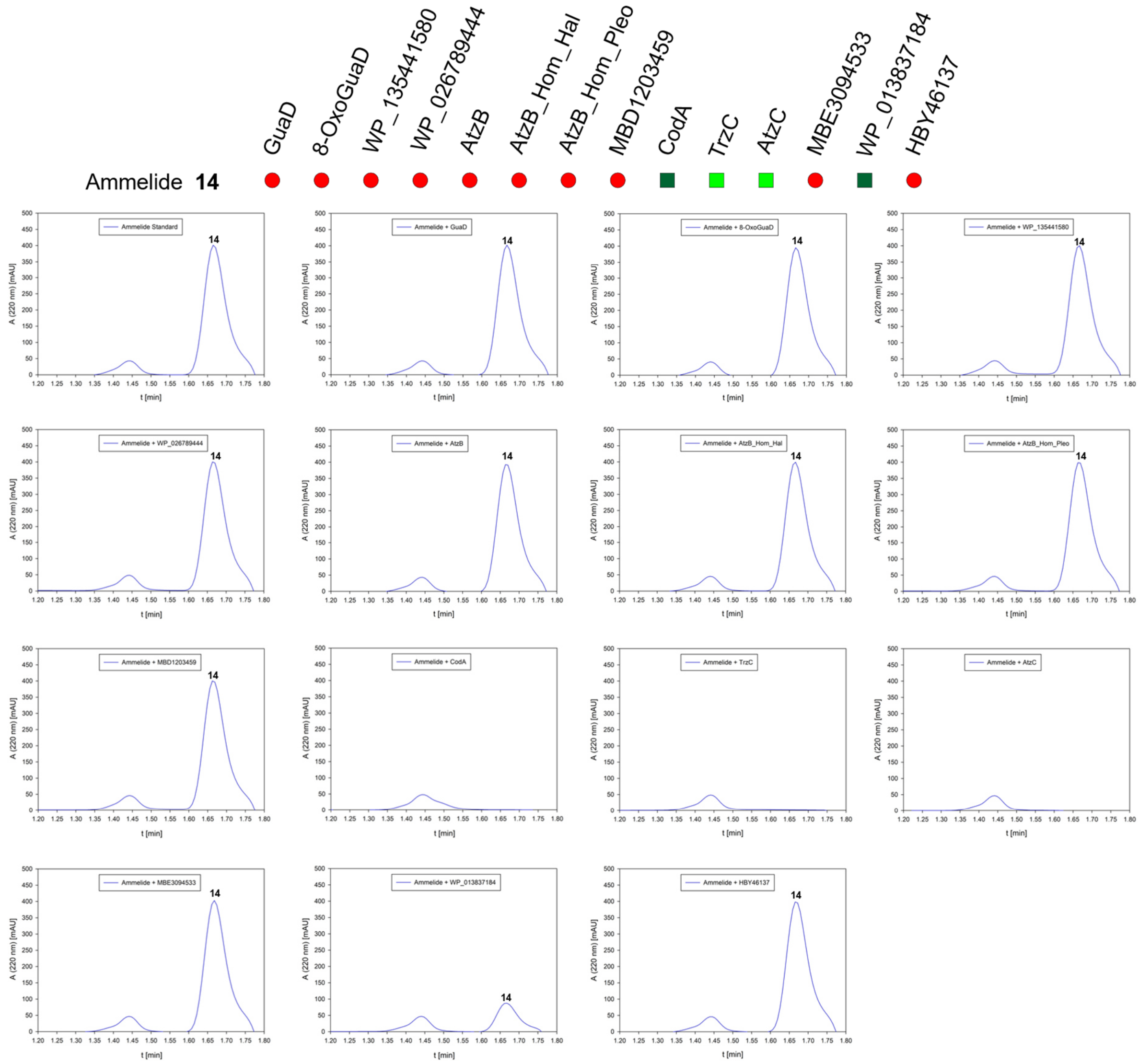

Source Data 14: HPLC-based analysis of the turnover of ammelide 14 to cyanuric acid 32 by different AHS enzymes. Substance standards as well as reaction mixtures of 500 µM **14**, 50 mM KP pH 7.5, and 2 µM of the tested enzyme were incubated at 25 °C and 500 rpm for 24 h. After centrifugation with a filter tube, the reaction products were analyzed via reversed-phase HPLC. Shown are the respective HPLC chromatograms at 220 nm. All substances were unambiguously identified using the retention times as well as the spectra of each peak. As **32** was neither detectable in its substance standard (not shown) nor in the reaction mixtures, the progress of the reaction was monitored by the decrease of the peak for **14**. Dark green squares indicate enzyme-substrate pairs for which substrate depletion was detected that has not been previously reported. Light green squares indicate enzyme-substrate pairs for which substrate depletion was detected, confirming data from the literature. Red circles indicate enzyme-substrate pairs for which no substrate depletion was detected.

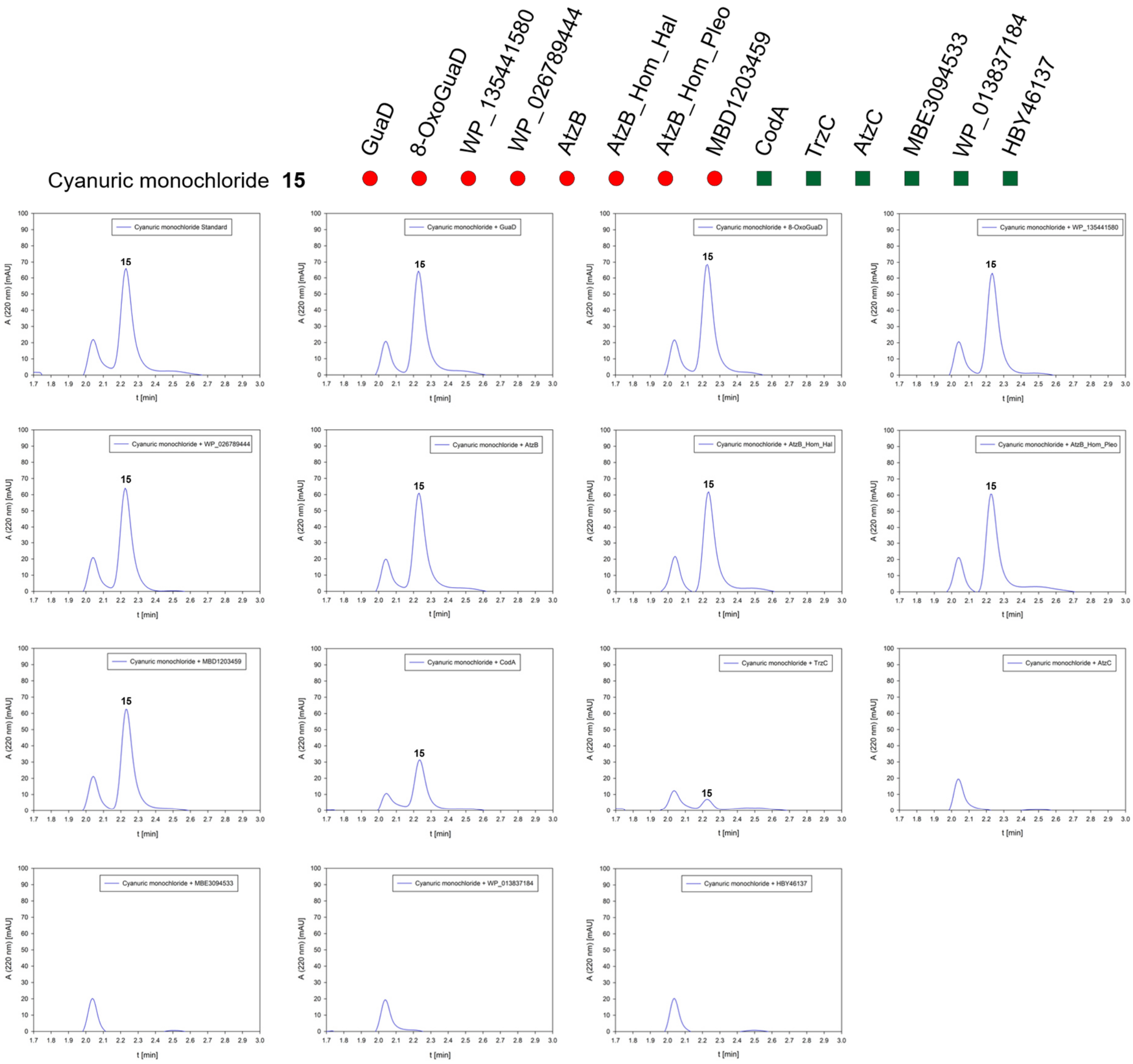

Source Data 15: HPLC-based analysis of the turnover of cyanuric monochloride 15 to cyanuric acid 32 by different AHS enzymes. Substance standards as well as reaction mixtures of 500 µM **15**, 50 mM KP pH 7.5, and 2 µM of the tested enzyme were incubated at 25 °C and 500 rpm for 24 h. After centrifugation with a filter tube, the reaction products were analyzed via reversed-phase HPLC. Shown are the respective HPLC chromatograms at 220 nm. All substances were unambiguously identified using the retention times as well as the spectra of each peak. As **32** was neither detectable in its substance standard (not shown) nor in the reaction mixtures, the progress of the reaction was monitored by the decrease of the peak for **15**. Dark green squares indicate enzyme-substrate pairs for which substrate depletion was detected that has not been previously reported. Light green squares indicate enzyme-substrate pairs for which substrate depletion was detected, confirming data from the literature. Red circles indicate enzyme-substrate pairs for which no substrate depletion was detected.

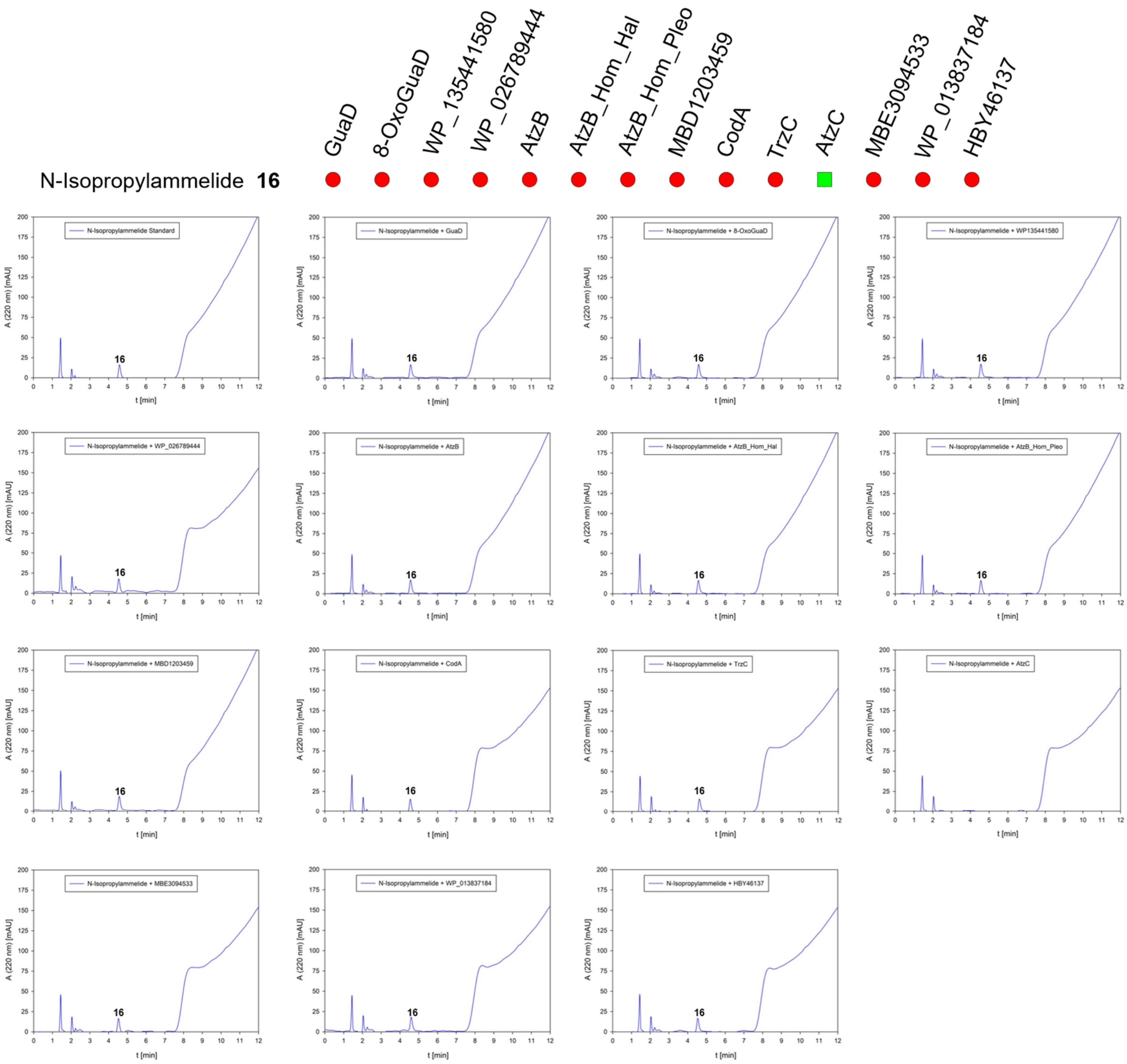

Source Data 16: HPLC-based analysis of the turnover of N-isopropylammelide 16 to cyanuric acid 32 by different AHS enzymes. Substance standards as well as reaction mixtures of 500 µM **16**, 50 mM KP pH 7.5, and 2 µM of the tested enzyme were incubated at 25 °C and 500 rpm for 24 h. After centrifugation with a filter tube, the reaction products were analyzed via reversed-phase HPLC. Shown are the respective HPLC chromatograms at 220 nm. All substances were unambiguously identified using the retention times as well as the spectra of each peak. As **32** was neither detectable in its substance standard (not shown) nor in the reaction mixtures, the progress of the reaction was monitored by the decrease of the peak for **16**. Dark green squares indicate enzyme-substrate pairs for which substrate depletion was detected that has not been previously reported. Light green squares indicate enzyme-substrate pairs for which substrate depletion was detected, confirming data from the literature. Red circles indicate enzyme-substrate pairs for which no substrate depletion was detected.

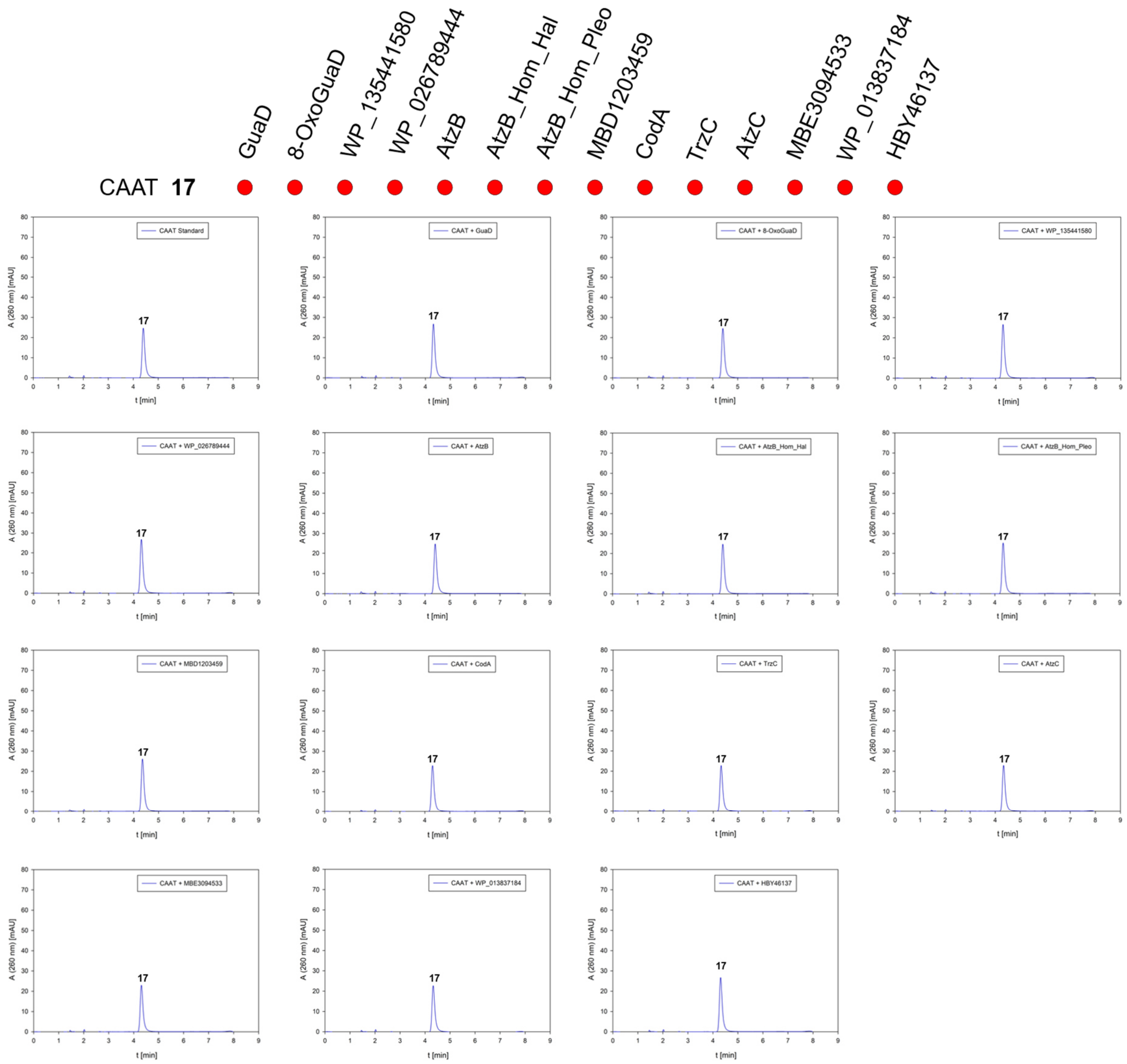

Source Data 17: HPLC-based analysis of the turnover of CAAT (2-chloro-4,6-diamino-1,3,5-triazine) 17 by different AHS enzymes. Substance standards as well as reaction mixtures of 500 µM **17**, 50 mM KP pH 7.5, and 2 µM of the tested enzyme were incubated at 25 °C and 500 rpm for 24 h. After centrifugation with a filter tube, the reaction products were analyzed via reversed-phase HPLC. Shown are the respective HPLC chromatograms at 260 nm. All substances were unambiguously identified using the retention times as well as the spectra of each peak. Red circles indicate enzyme-substrate pairs for which no product formation was detected.

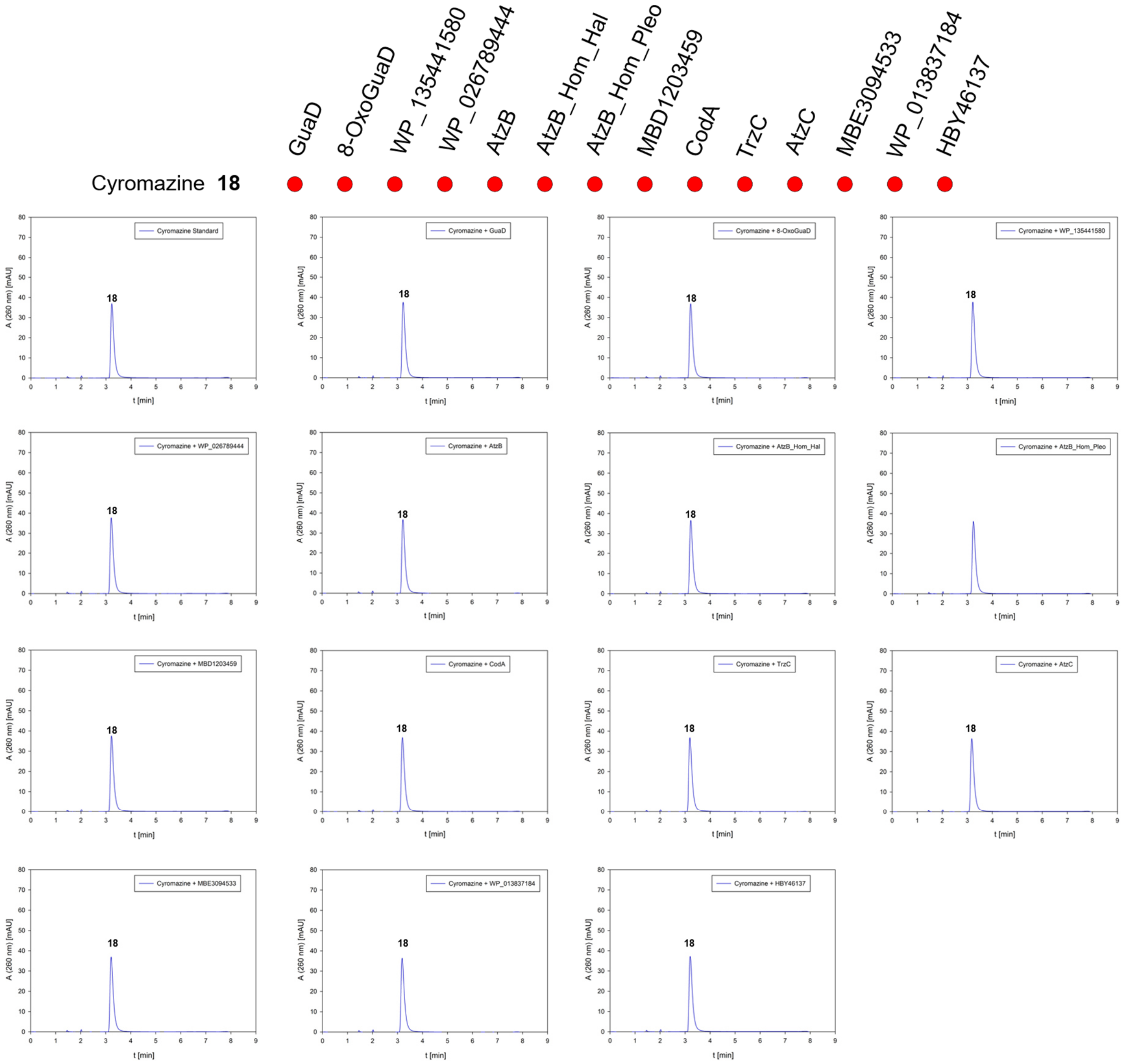

Source Data 18: HPLC-based analysis of the turnover of cyromazine 18 by different AHS enzymes. Substance standards as well as reaction mixtures of 500 µM **18**, 50 mM KP pH 7.5, and 2 µM of the tested enzyme were incubated at 25 °C and 500 rpm for 24 h. After centrifugation with a filter tube, the reaction products were analyzed via reversed-phase HPLC. Shown are the respective HPLC chromatograms at 260 nm. All substances were unambiguously identified using the retention times as well as the spectra of each peak. Red circles indicate enzyme-substrate pairs for which no product formation was detected.

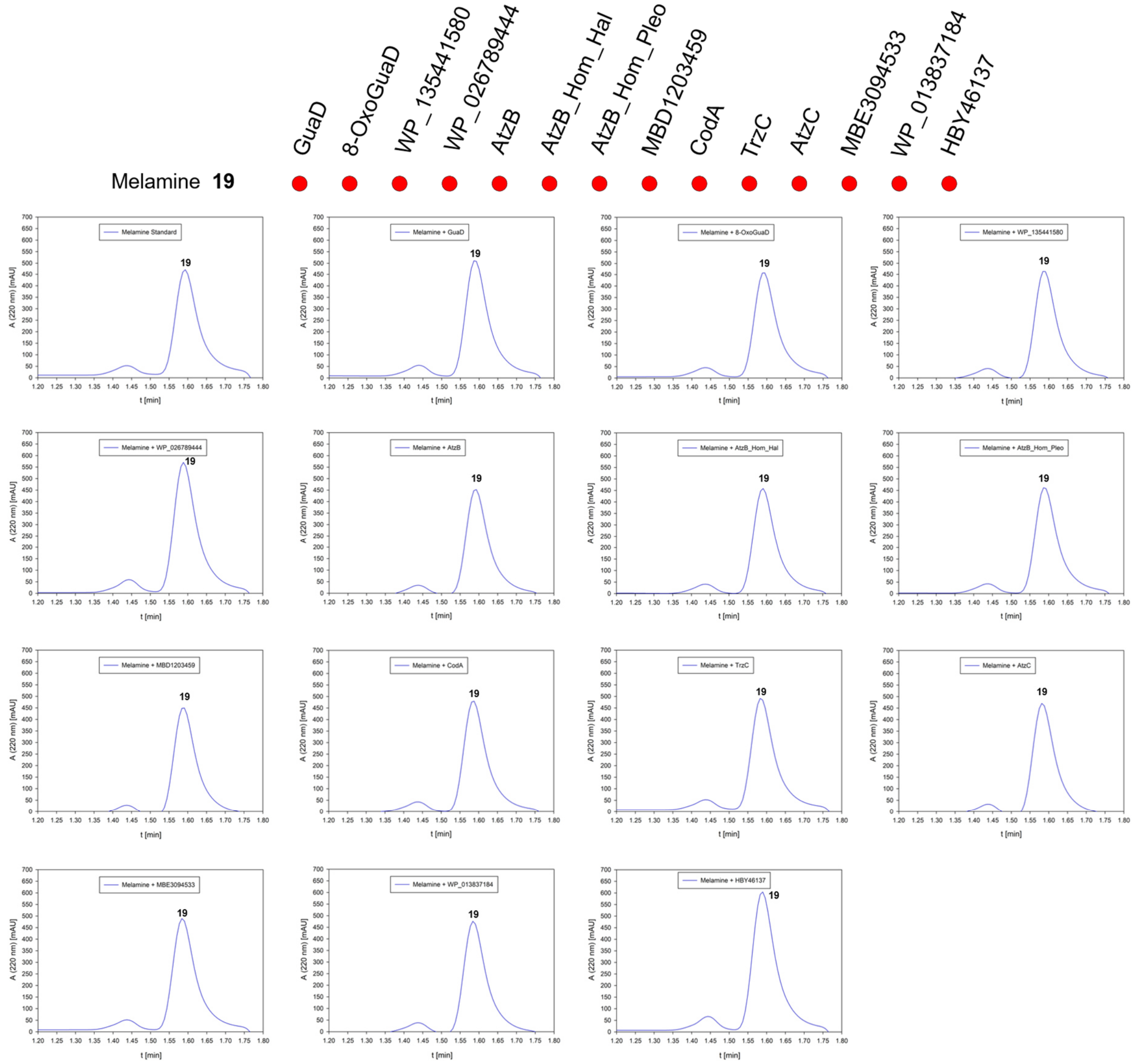

Source Data 19: HPLC-based analysis of the turnover of melamine 19 by different AHS enzymes. Substance standards as well as reaction mixtures of 500 µM **19**, 50 mM KP pH 7.5, and 2 µM of the tested enzyme were incubated at 25 °C and 500 rpm for 24 h. After centrifugation with a filter tube, the reaction products were analyzed via reversed-phase HPLC. Shown are the respective HPLC chromatograms at 220 nm. All substances were unambiguously identified using the retention times as well as the spectra of each peak. Red circles indicate enzyme-substrate pairs for which no product formation was detected.

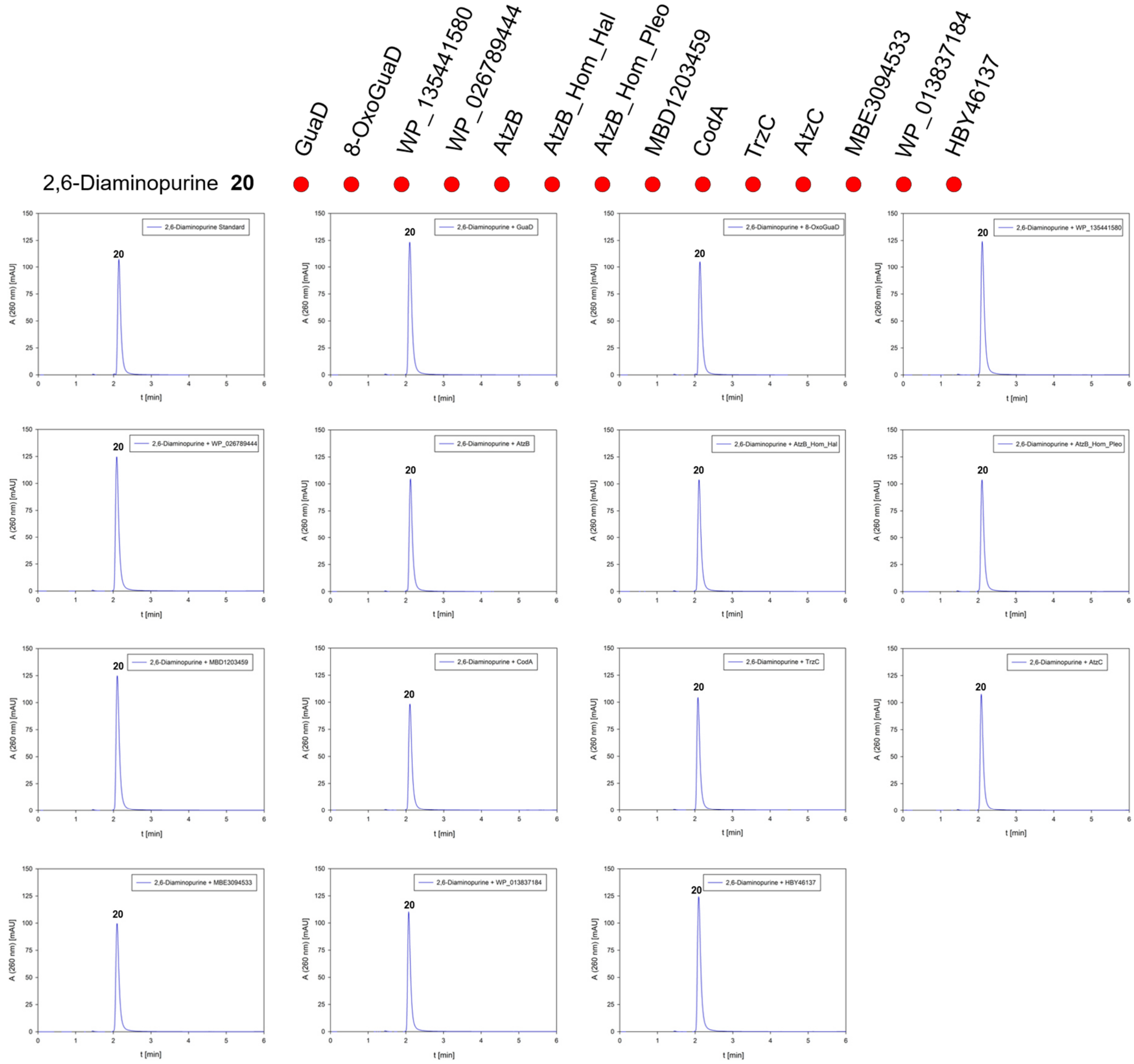

Source Data 20: HPLC-based analysis of the turnover of 2,6-diaminopurine 20 by different AHS enzymes. Substance standards as well as reaction mixtures of 500 µM **20**, 50 mM KP pH 7.5, and 2 µM of the tested enzyme were incubated at 25 °C and 500 rpm for 24 h. After centrifugation with a filter tube, the reaction products were analyzed via reversed-phase HPLC. Shown are the respective HPLC chromatograms at 260 nm. All substances were unambiguously identified using the retention times as well as the spectra of each peak. Red circles indicate enzyme-substrate pairs for which no product formation was detected.

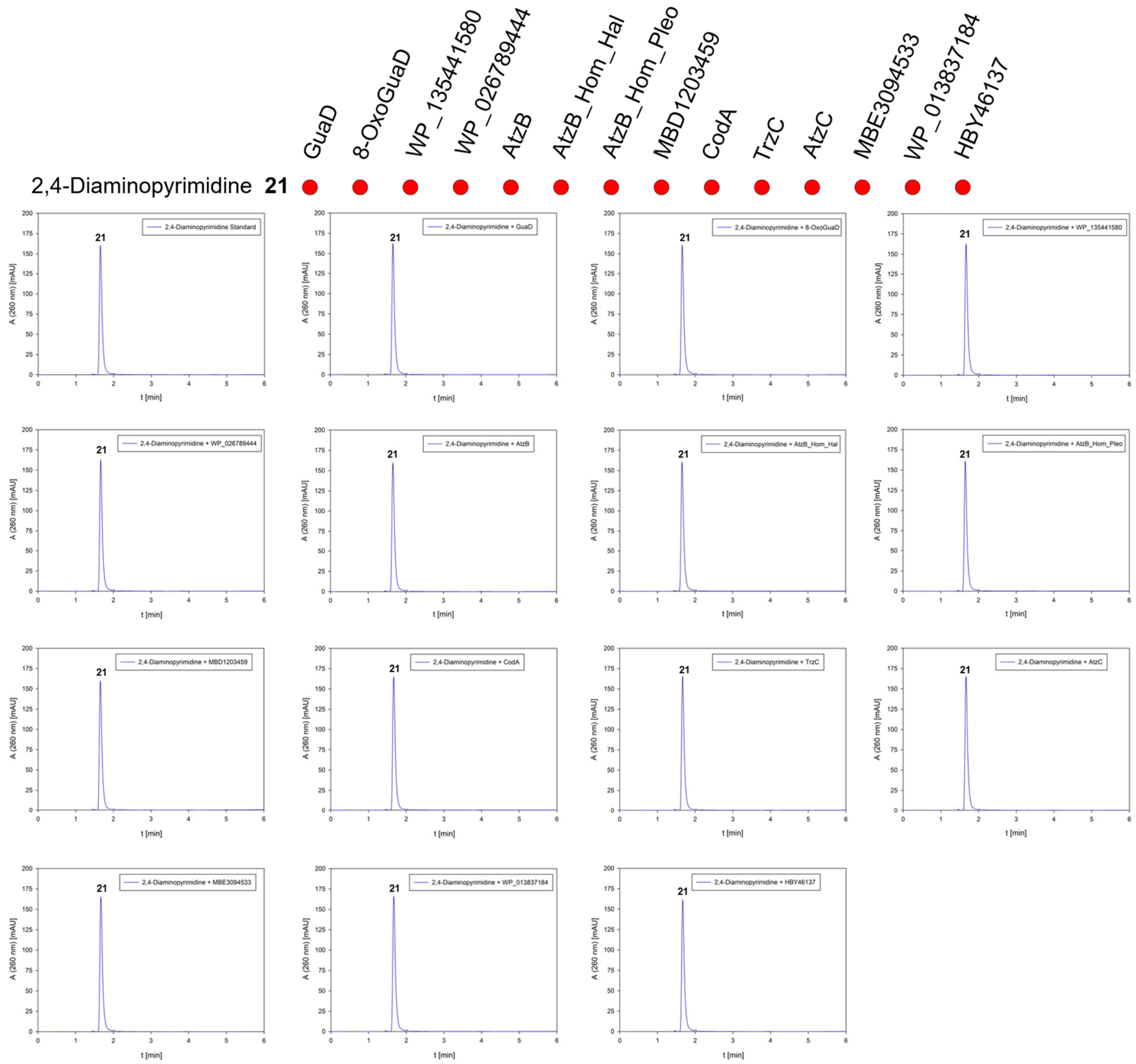

Source Data 21: HPLC-based analysis of the turnover of 2,4-diaminopyrimidine 21 by different AHS enzymes. Substance standards as well as reaction mixtures of 500 µM **21**, 50 mM KP pH 7.5, and 2 µM of the tested enzyme were incubated at 25 °C and 500 rpm for 24 h. After centrifugation with a filter tube, the reaction products were analyzed via reversed-phase HPLC. Shown are the respective HPLC chromatograms at 260 nm. All substances were unambiguously identified using the retention times as well as the spectra of each peak. Red circles indicate enzyme-substrate pairs for which no product formation was detected.

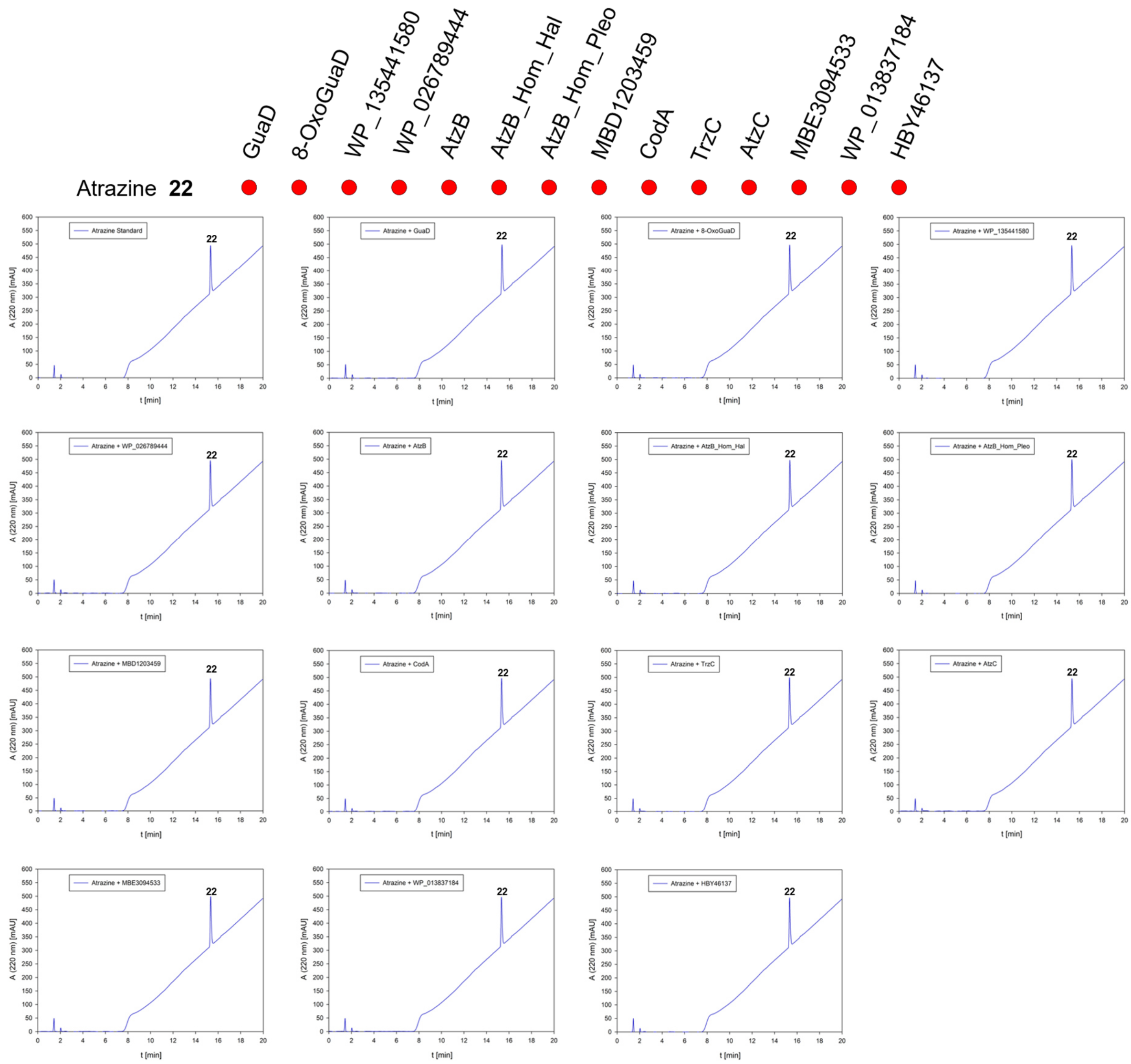

Source Data 22: HPLC-based analysis of the turnover of atrazine 22 by different AHS enzymes. Substance standards as well as reaction mixtures of 500 µM **22**, 50 mM KP pH 7.5, and 2 µM of the tested enzyme were incubated at 25 °C and 500 rpm for 24 h. After centrifugation with a filter tube, the reaction products were analyzed via reversed-phase HPLC. Shown are the respective HPLC chromatograms at 220 nm. All substances were unambiguously identified using the retention times as well as the spectra of each peak. Red circles indicate enzyme-substrate pairs for which no product formation was detected.

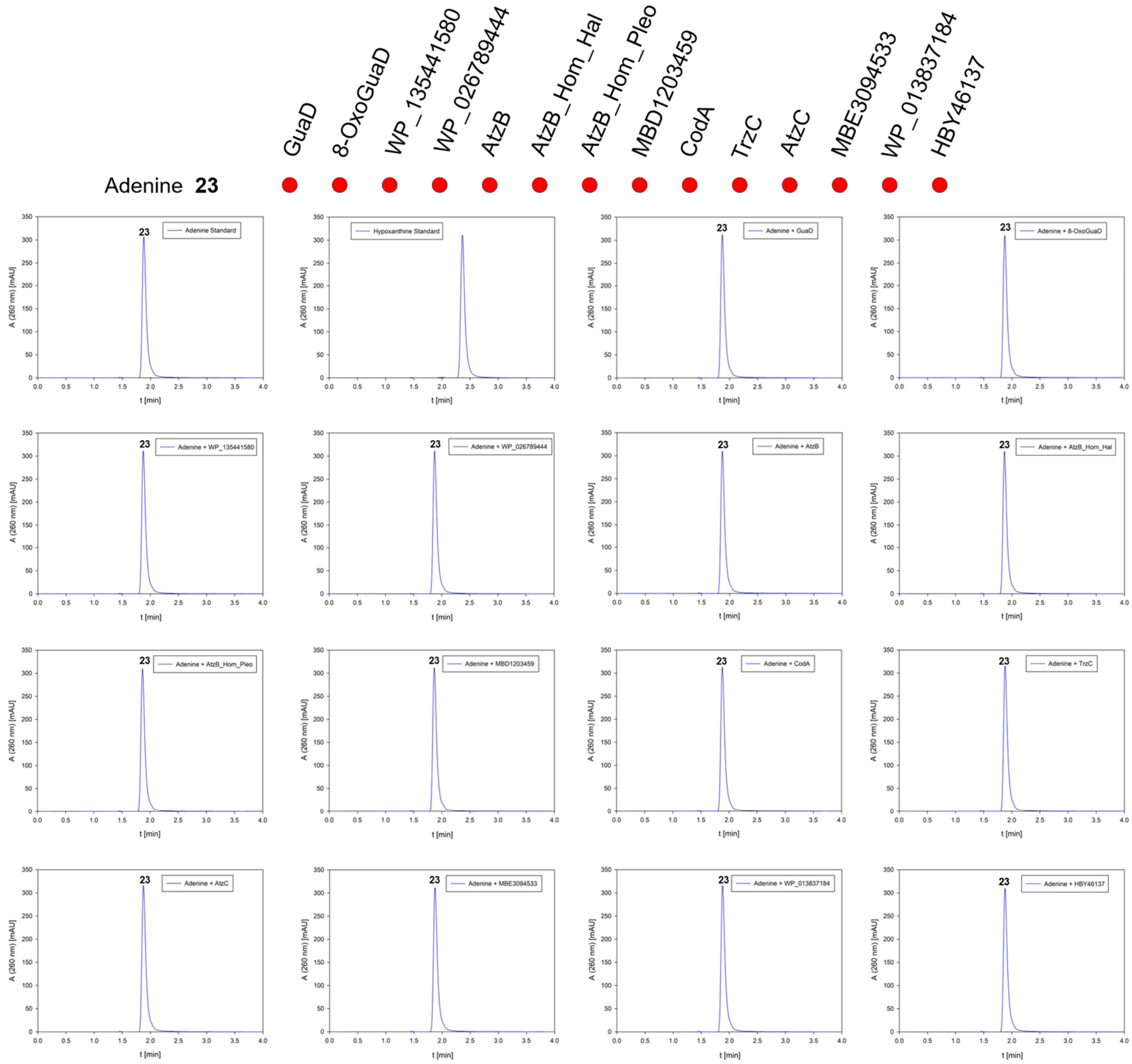

Source Data 23: HPLC-based analysis of the turnover of adenine 23 by different AHS enzymes. Substance standards as well as reaction mixtures of 500 µM **23**, 50 mM KP pH 7.5, and 2 µM of the tested enzyme were incubated at 25 °C and 500 rpm for 24 h. After centrifugation with a filter tube, the reaction products were analyzed via reversed-phase HPLC. Shown are the respective HPLC chromatograms at 260 nm. All substances were unambiguously identified using the retention times as well as the spectra of each peak. Red circles indicate enzyme-substrate pairs for which no product formation was detected.

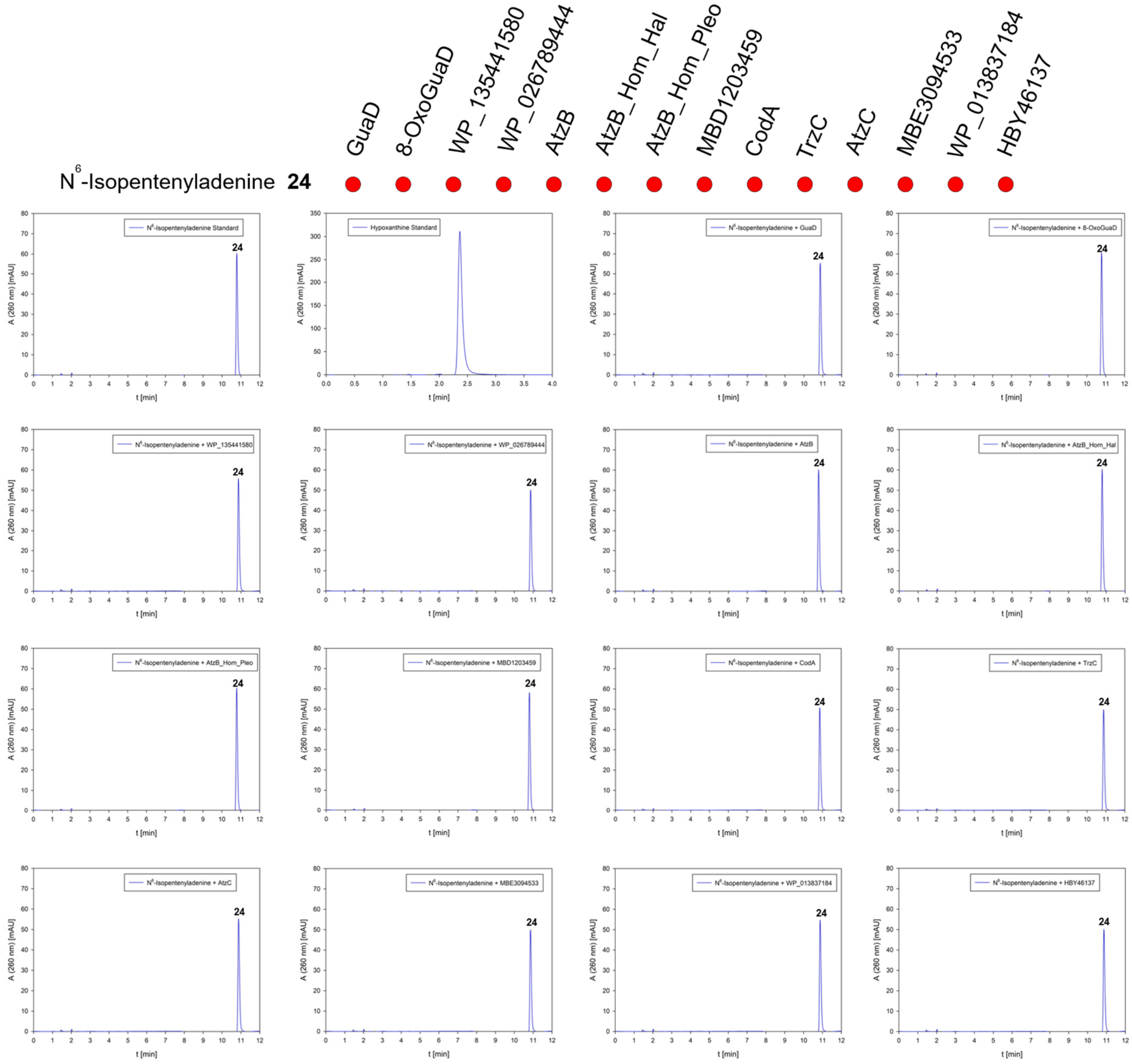

Source Data 24: HPLC-based analysis of the turnover of N⁶-isopentenyladenine 24 by different AHS enzymes. Substance standards as well as reaction mixtures of 500 µM **24**, 50 mM KP pH 7.5, and 2 µM of the tested enzyme were incubated at 25 °C and 500 rpm for 24 h. After centrifugation with a filter tube, the reaction products were analyzed via reversed-phase HPLC. Shown are the respective HPLC chromatograms at 260 nm. All substances were unambiguously identified using the retention times as well as the spectra of each peak. Red circles indicate enzyme-substrate pairs for which no product formation was detected.

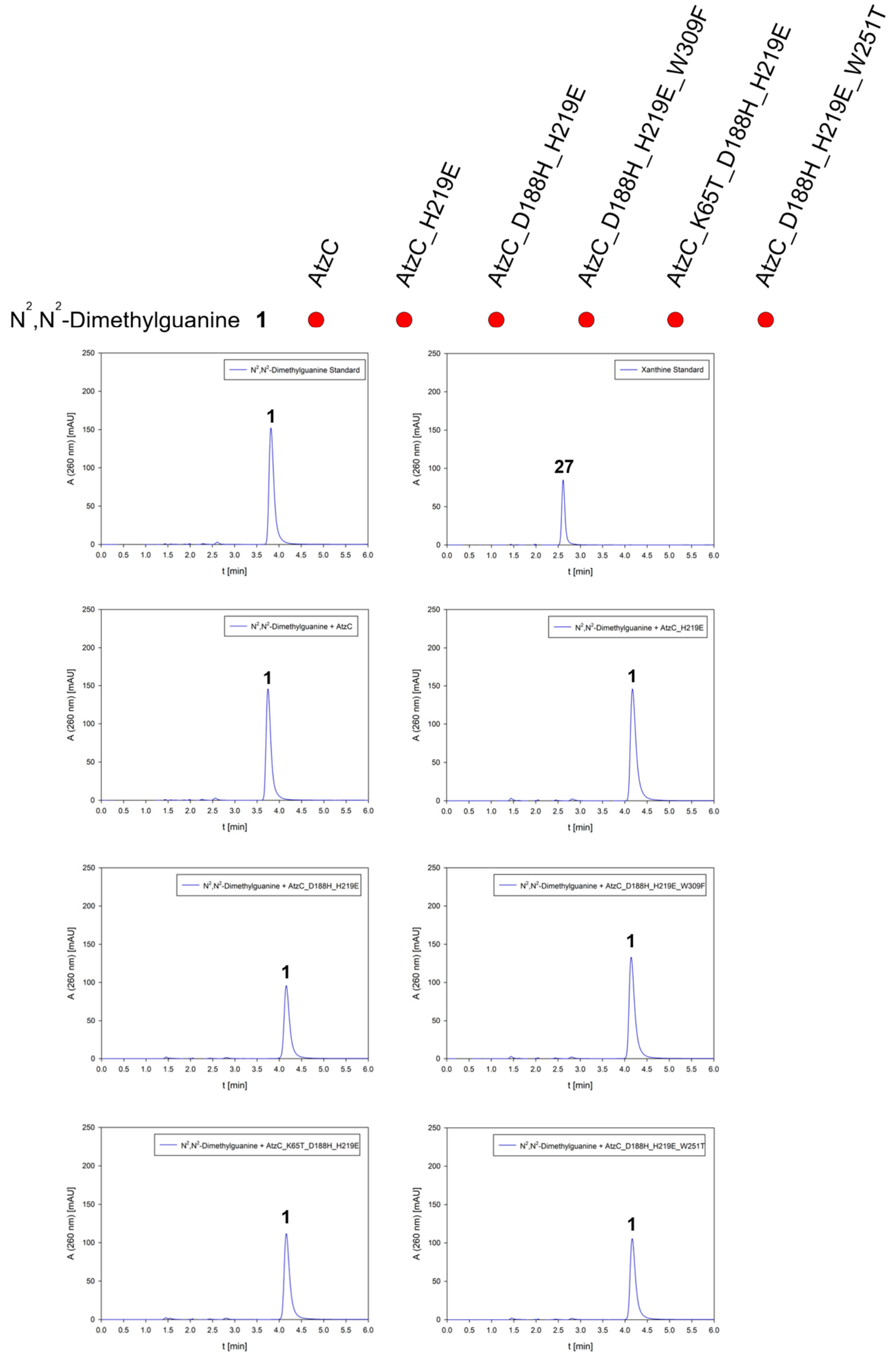

Source Data 25: HPLC-based analysis of the turnover of N^2,N^2-dimethylguanine 1 by AtzC wildtype and variants. Substance standards as well as reaction mixtures of 500 µM **1**, 50 mM KP pH 7.5, and 2 µM of the tested enzyme were incubated at 25 °C and 500 rpm for 24 h. After centrifugation with a filter tube, the reaction products were analyzed via reversed-phase HPLC. Shown are the respective HPLC chromatograms at 260 nm. All substances were unambiguously identified using the retention times as well as the spectra of each peak. Red circles indicate enzyme-substrate pairs for which no product formation was detected.

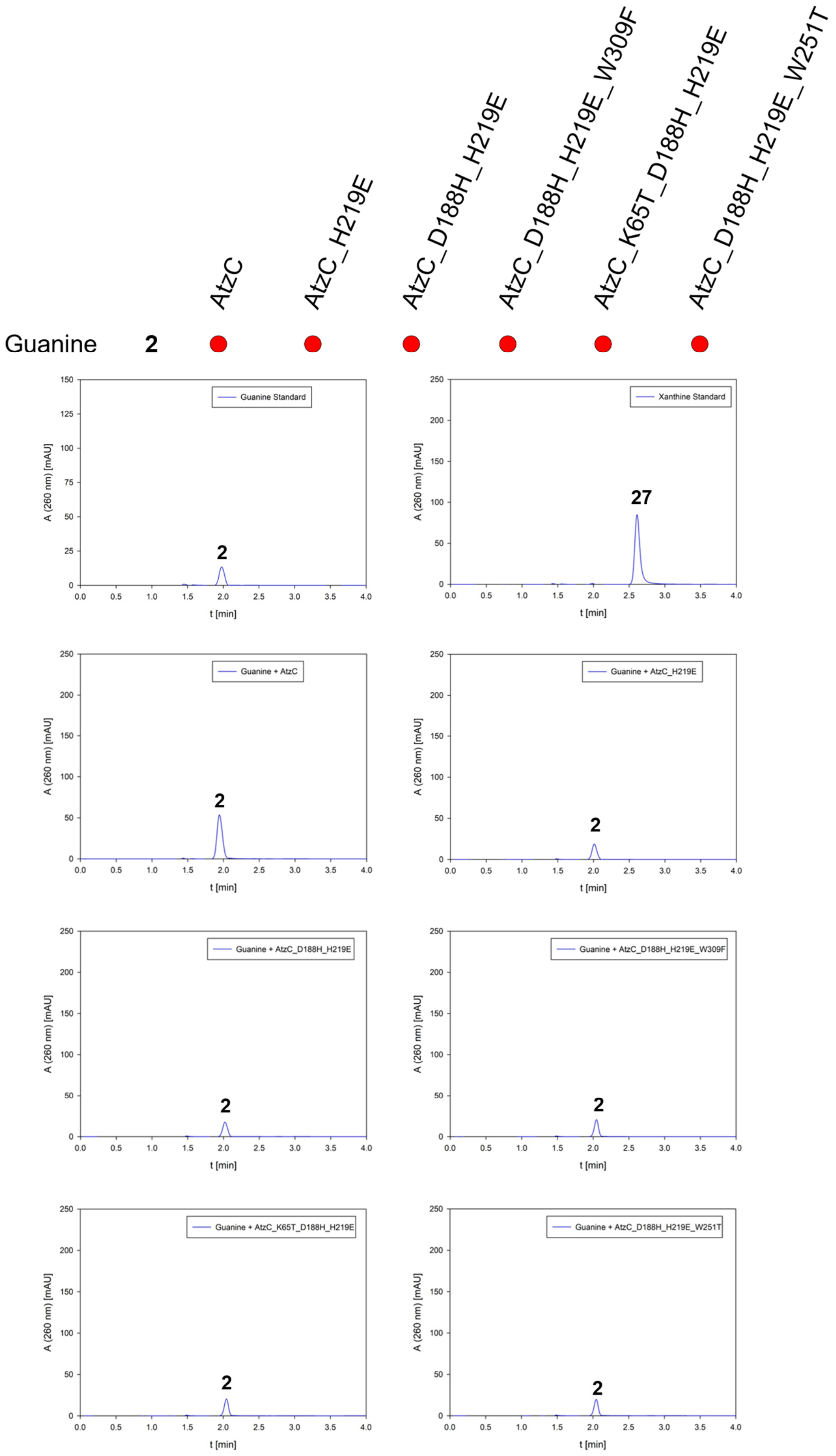

Source Data 26: HPLC-based analysis of the turnover of guanine 2 by AtzC wildtype and variants. Substance standards as well as reaction mixtures of 500 µM **2**, 50 mM KP pH 7.5, and 2 µM of the tested enzyme were incubated at 25 °C and 500 rpm for 24 h. After centrifugation with a filter tube, the reaction products were analyzed via reversed-phase HPLC. Shown are the respective HPLC chromatograms at 260 nm. All substances were unambiguously identified using the retention times as well as the spectra of each peak. Red circles indicate enzyme-substrate pairs for which no product formation was detected.

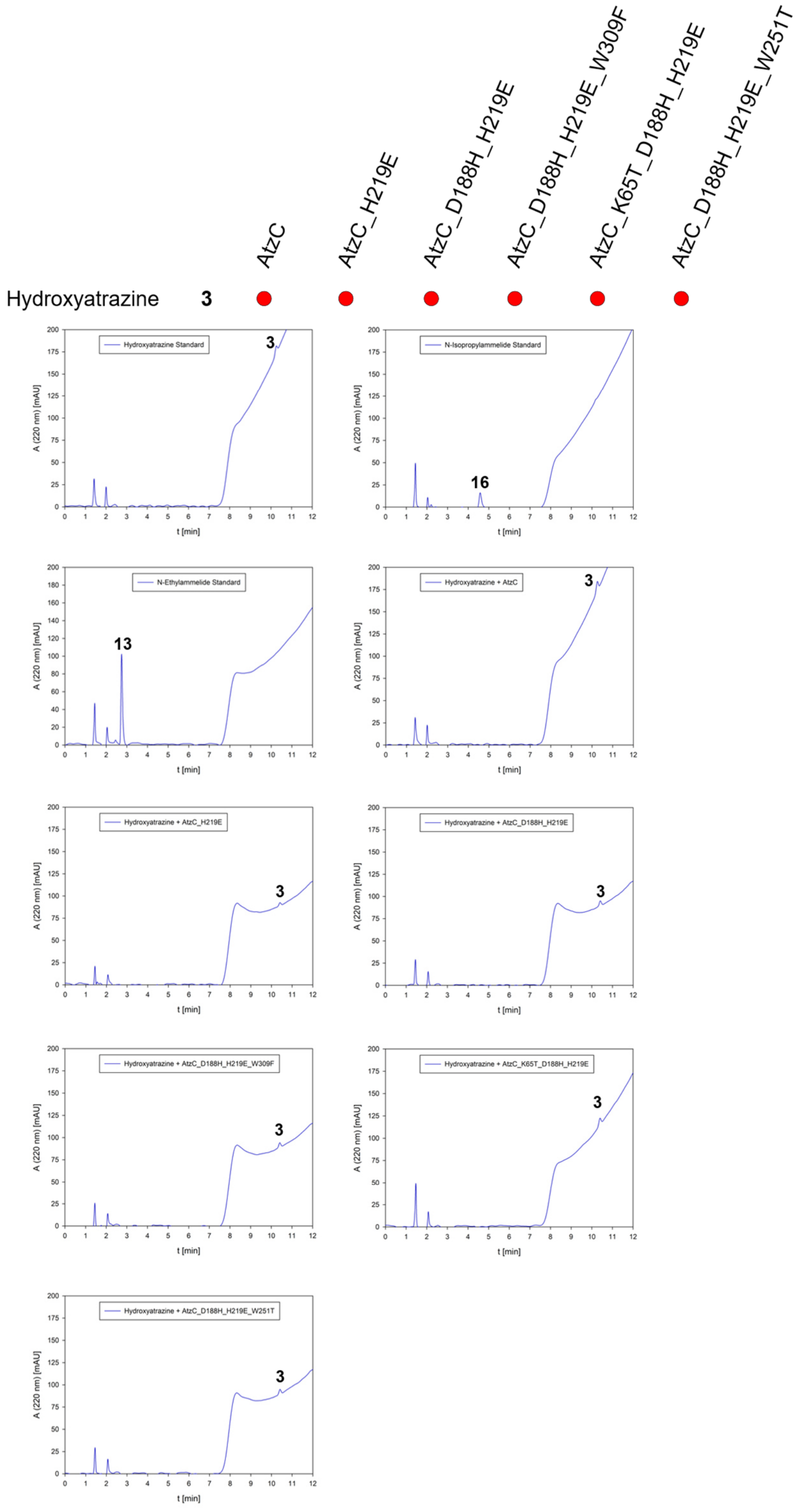

Source Data 27: HPLC-based analysis of the turnover of hydroxyatrazine 3 by AtzC wildtype and variants. Substance standards as well as reaction mixtures of 500 µM **3**, 50 mM KP pH 7.5, and 2 µM of the tested enzyme were incubated at 25 °C and 500 rpm for 24 h. After centrifugation with a filter tube, the reaction products were analyzed via reversed-phase HPLC. Shown are the respective HPLC chromatograms at 220 nm. All substances were unambiguously identified using the retention times as well as the spectra of each peak. Red circles indicate enzyme-substrate pairs for which no product formation was detected.

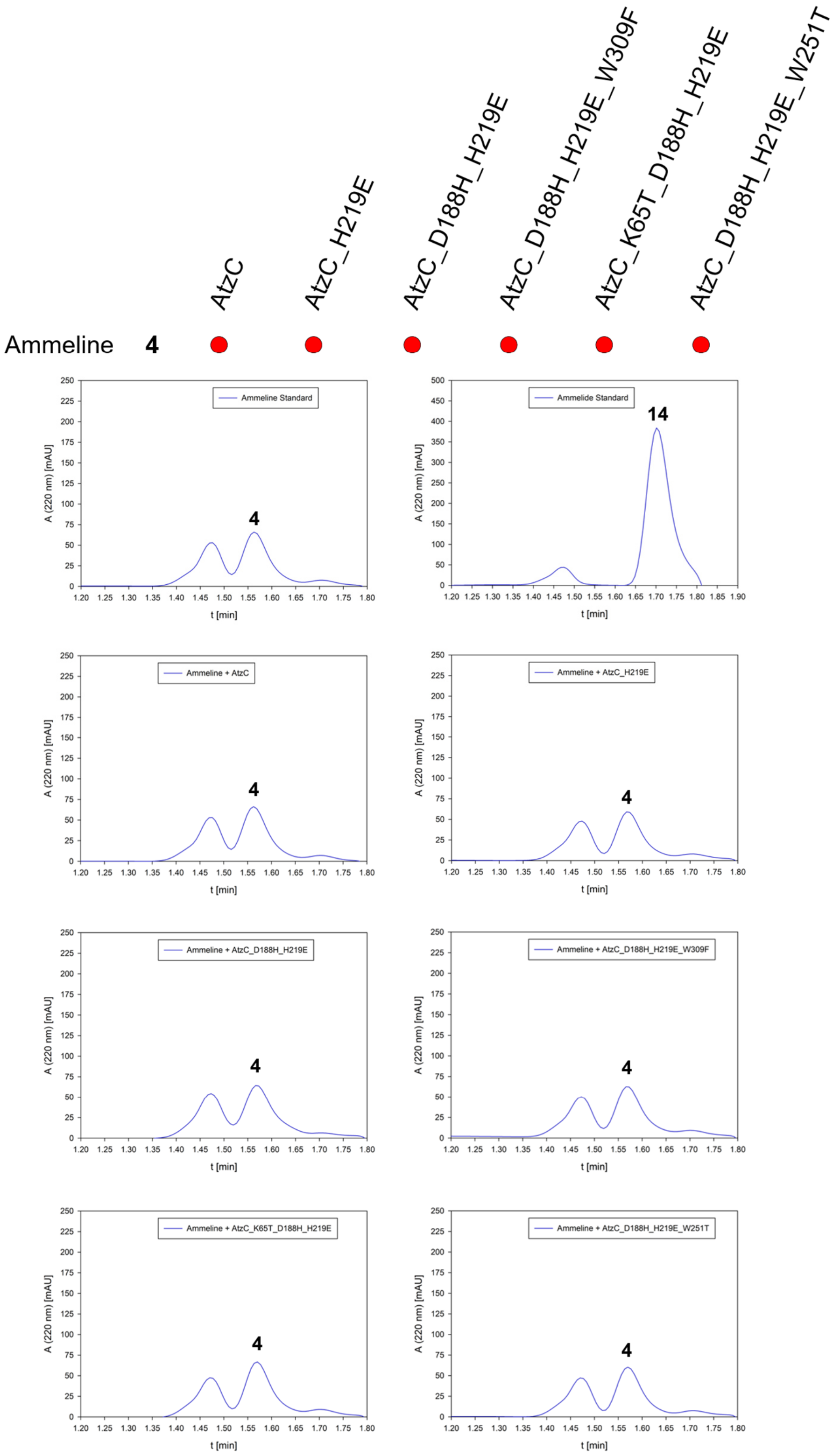

Source Data 28: HPLC-based analysis of the turnover of ammeline 4 by AtzC wildtype and variants. Substance standards as well as reaction mixtures of 500 µM **4**, 50 mM KP pH 7.5, and 2 µM of the tested enzyme were incubated at 25 °C and 500 rpm for 24 h. After centrifugation with a filter tube, the reaction products were analyzed via reversed-phase HPLC. Shown are the respective HPLC chromatograms at 220 nm. All substances were unambiguously identified using the retention times as well as the spectra of each peak. Red circles indicate enzyme-substrate pairs for which no product formation was detected.

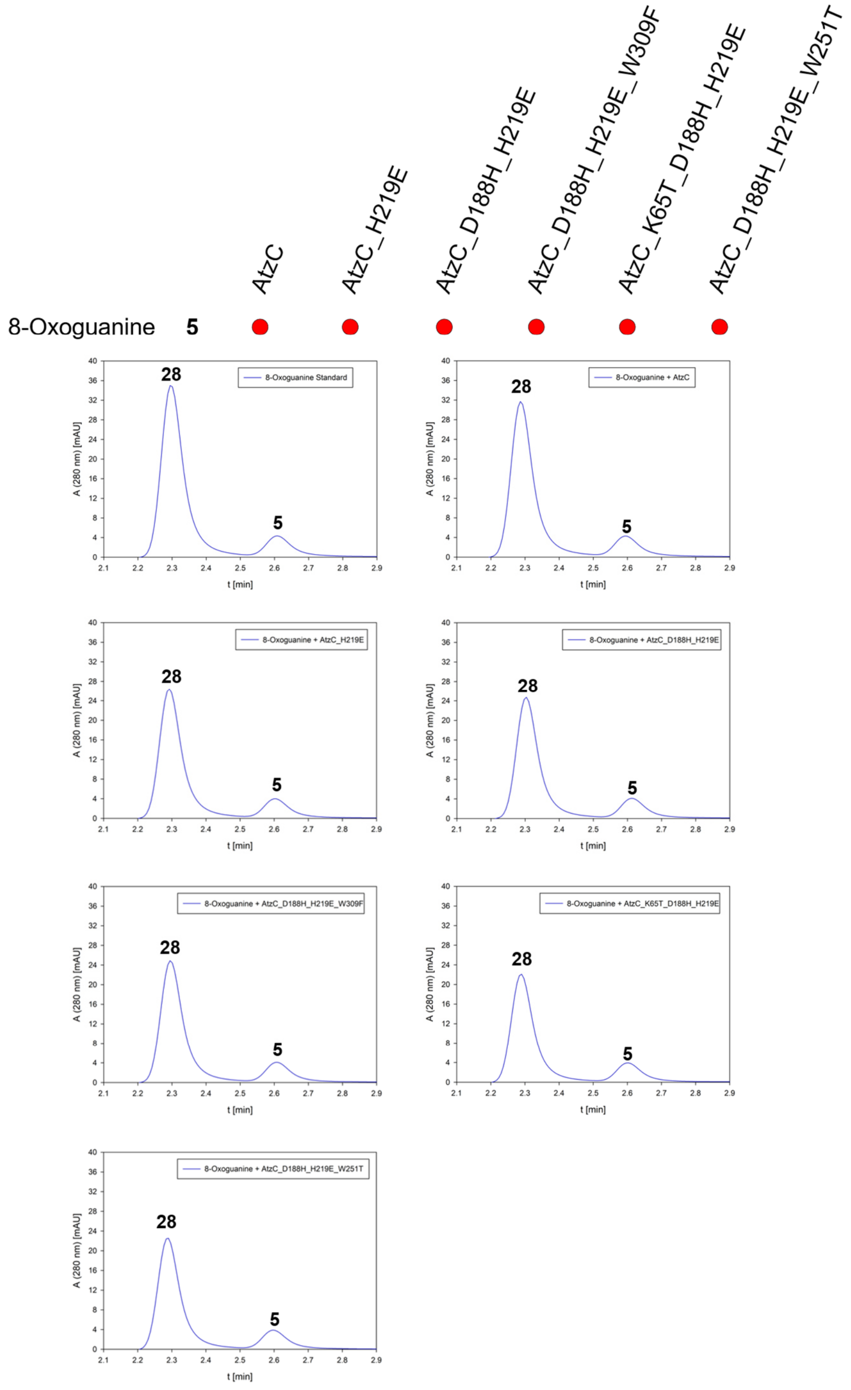

Source Data 29: HPLC-based analysis of the turnover of 8-oxoguanine 5 by AtzC wildtype and variants. Substance standards as well as reaction mixtures of 500 µM **5**, 50 mM KP pH 7.5, and 2 µM of the tested enzyme were incubated at 25 °C and 500 rpm for 24 h. After centrifugation with a filter tube, the reaction products were analyzed via reversed-phase HPLC. Shown are the respective HPLC chromatograms at 280 nm. All substances were unambiguously identified using the retention times as well as the spectra of each peak. Due to spontaneous hydrolysis of **5**, a considerable amount of **28** is already formed in the substrate standard. Red circles indicate enzyme-substrate pairs for which no product formation and no substrate depletion was detected compared to the standard.

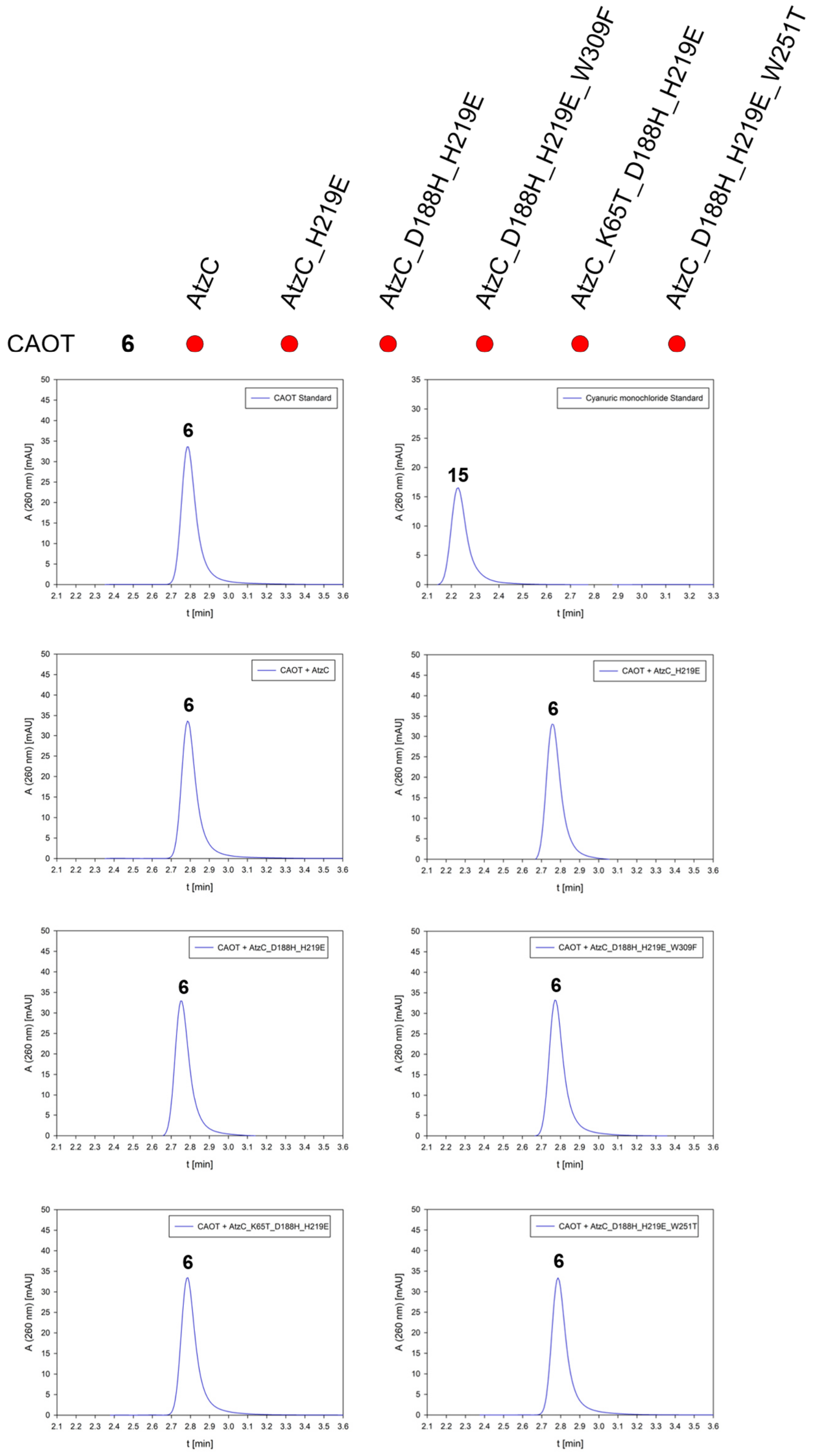

Source Data 30: HPLC-based analysis of the turnover of CAOT (2-chloro-4-amino-6-hydroxy-1,3,5-triazine) 6 by AtzC wildtype and variants. Substance standards as well as reaction mixtures of 500 µM **6**, 50 mM KP pH 7.5, and 2 µM of the tested enzyme were incubated at 25 °C and 500 rpm for 24 h. After centrifugation with a filter tube, the reaction products were analyzed via reversed-phase HPLC. Shown are the respective HPLC chromatograms at 260 nm. All substances were unambiguously identified using the retention times as well as the spectra of each peak. Red circles indicate enzyme-substrate pairs for which no product formation was detected.

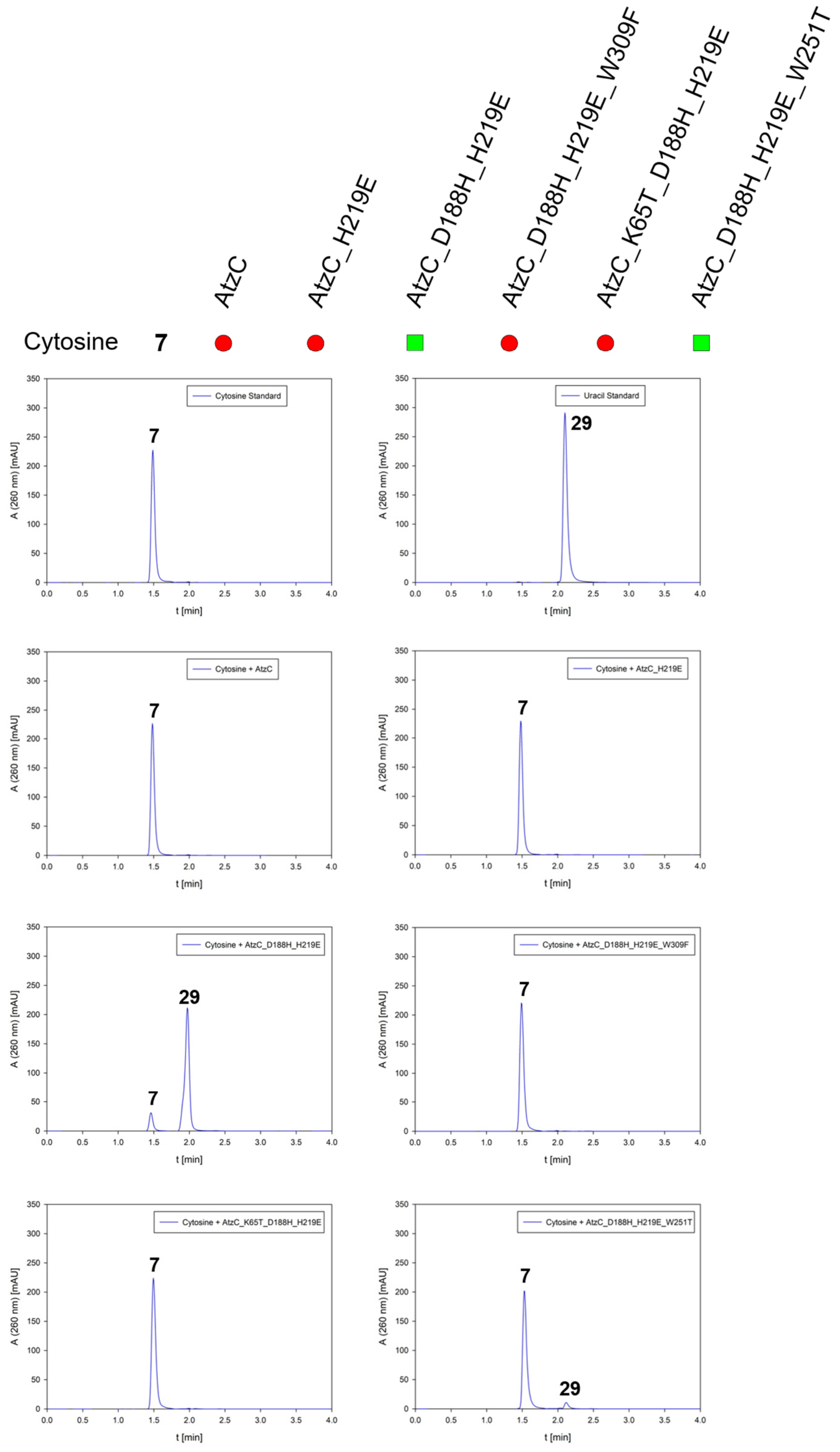

Source Data 31: HPLC-based analysis of the turnover of cytosine 7 to uracil 29 by AtzC wildtype and variants. Substance standards as well as reaction mixtures of 500 µM **7**, 50 mM KP pH 7.5, and 2 µM of the tested enzyme were incubated at 25 °C and 500 rpm for 24 h. After centrifugation with a filter tube, the reaction products were analyzed via reversed-phase HPLC. Shown are the respective HPLC chromatograms at 260 nm. All substances were unambiguously identified using the retention times as well as the spectra of each peak. Light green squares indicate enzyme-substrate pairs for which product formation was detected. Red circles indicate enzyme-substrate pairs for which no product formation was detected.

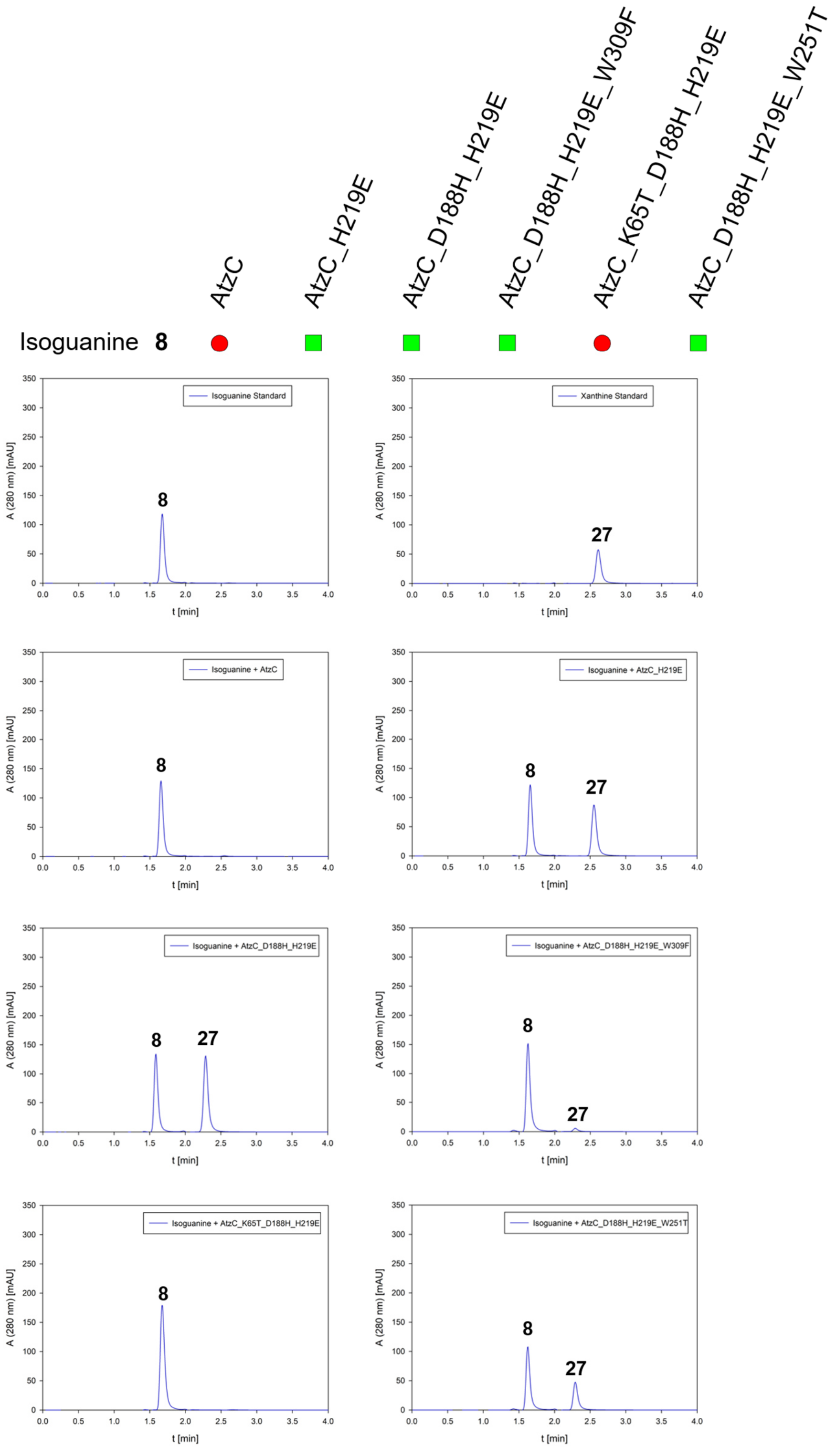

Source Data 32: HPLC-based analysis of the turnover of isoguanine 8 to xanthine 27 by AtzC wildtype and variants. Substance standards as well as reaction mixtures of 500 µM **8**, 50 mM KP pH 7.5, and 2 µM of the tested enzyme were incubated at 25 °C and 500 rpm for 24 h. After centrifugation with a filter tube, the reaction products were analyzed via reversed-phase HPLC. Shown are the respective HPLC chromatograms at 280 nm. All substances were unambiguously identified using the retention times as well as the spectra of each peak. Light green squares indicate enzyme-substrate pairs for which product formation was detected. Red circles indicate enzyme-substrate pairs for which no product formation was detected.

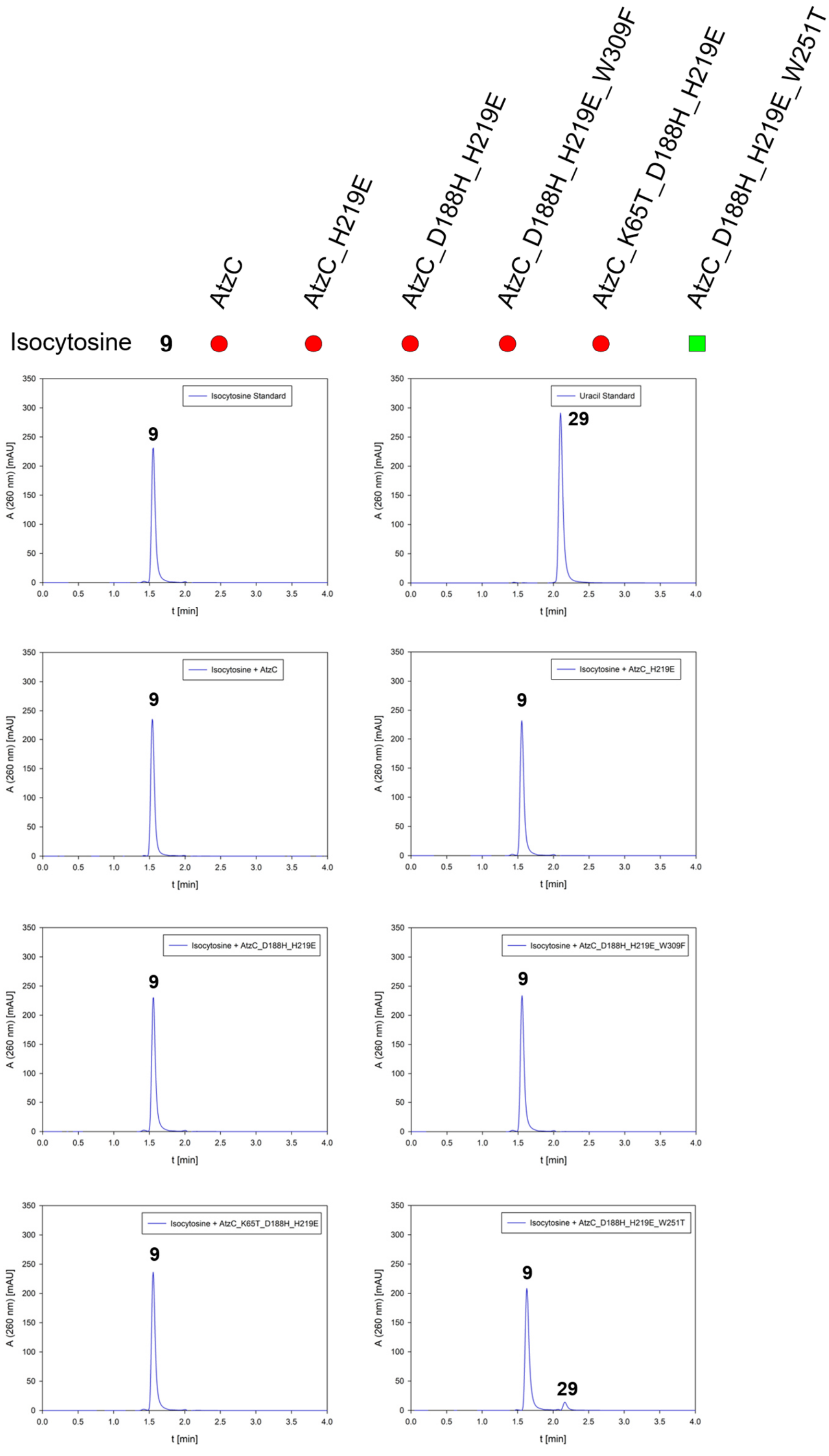

Source Data 33: HPLC-based analysis of the turnover of isocytosine 9 to uracil 29 by AtzC wildtype and variants. Substance standards as well as reaction mixtures of 500 µM **9**, 50 mM KP pH 7.5, and 2 µM of the tested enzyme were incubated at 25 °C and 500 rpm for 24 h. After centrifugation with a filter tube, the reaction products were analyzed via reversed-phase HPLC. Shown are the respective HPLC chromatograms at 260 nm. All substances were unambiguously identified using the retention times as well as the spectra of each peak. Light green squares indicate enzyme-substrate pairs for which product formation was detected. Red circles indicate enzyme-substrate pairs for which no product formation was detected.

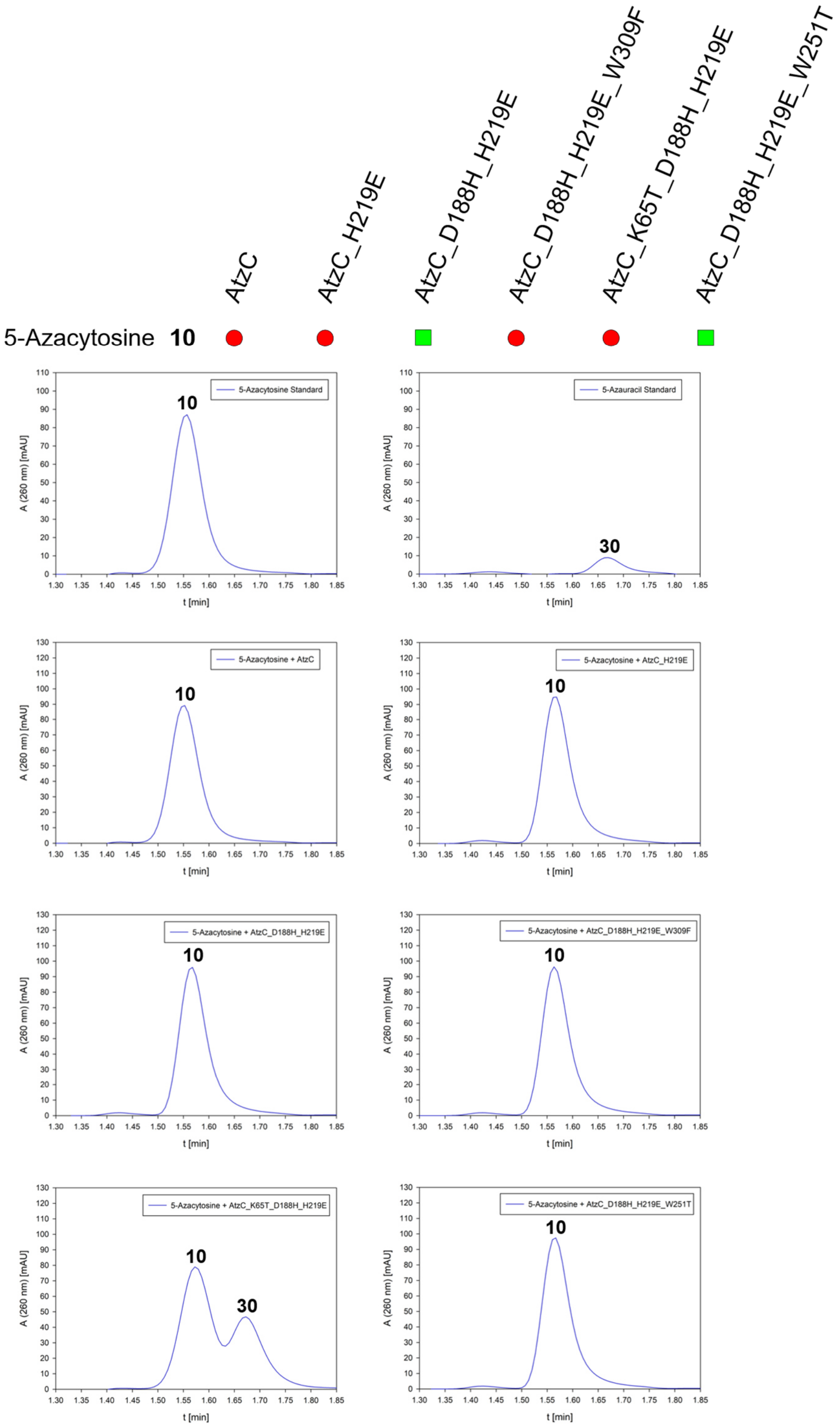

Source Data 34: HPLC-based analysis of the turnover of 5-azacytosine 10 to 5-azauracil 30 by AtzC wildtype and variants. Substance standards as well as reaction mixtures of 500 µM **10**, 50 mM KP pH 7.5, and 2 µM of the tested enzyme were incubated at 25 °C and 500 rpm for 24 h. After centrifugation with a filter tube, the reaction products were analyzed via reversed-phase HPLC. Shown are the respective HPLC chromatograms at 260 nm. All substances were unambiguously identified using the retention times as well as the spectra of each peak. Light green squares indicate enzyme-substrate pairs for which product formation was detected. Red circles indicate enzyme-substrate pairs for which no product formation was detected.

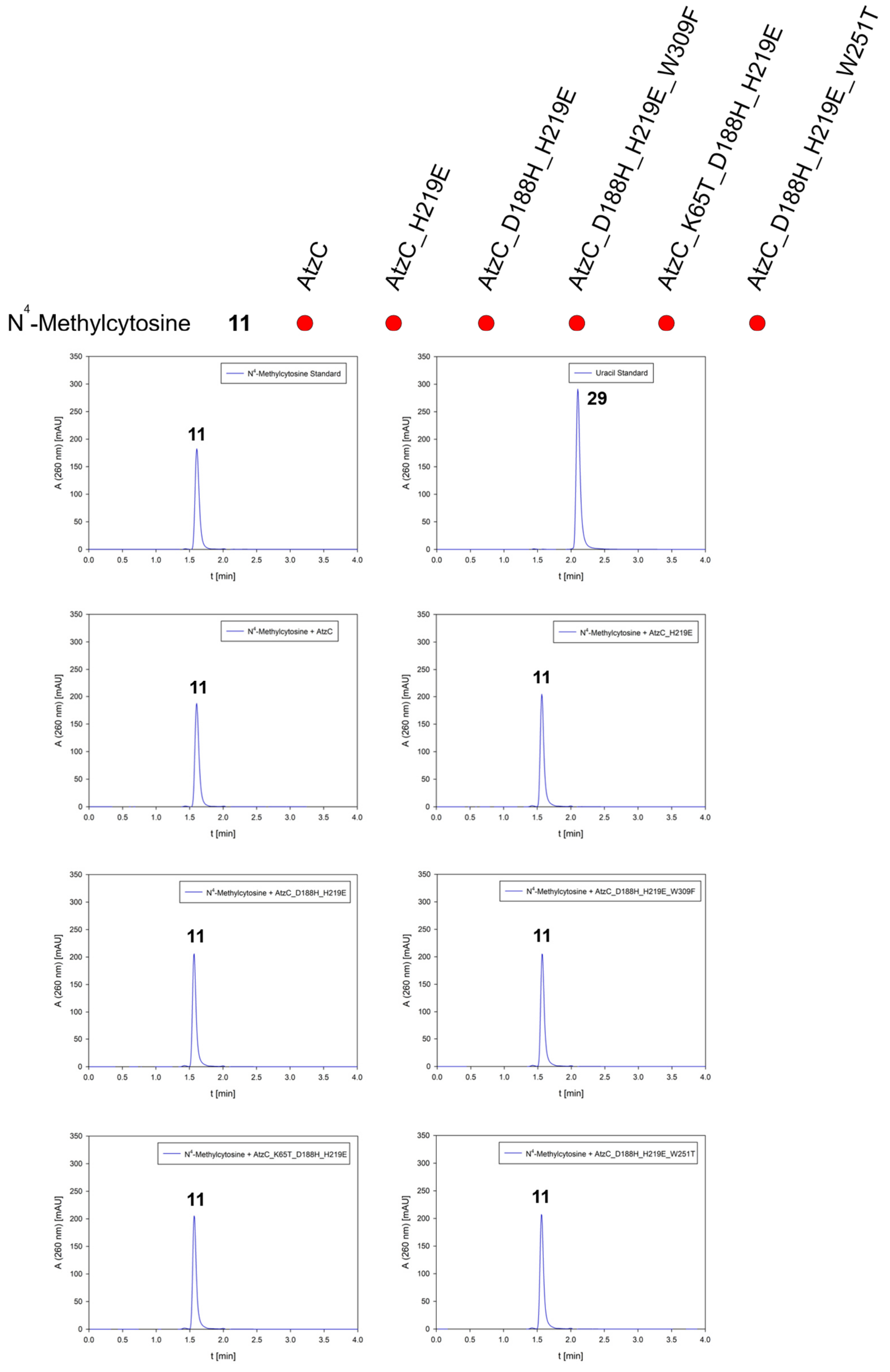

Source Data 35: HPLC-based analysis of the turnover of N^4-methylcytosine 11 by AtzC wildtype and variants. Substance standards as well as reaction mixtures of 500 µM **11**, 50 mM KP pH 7.5, and 2 µM of the tested enzyme were incubated at 25 °C and 500 rpm for 24 h. After centrifugation with a filter tube, the reaction products were analyzed via reversed-phase HPLC. Shown are the respective HPLC chromatograms at 260 nm. All substances were unambiguously identified using the retention times as well as the spectra of each peak. Red circles indicate enzyme-substrate pairs for which no product formation was detected.

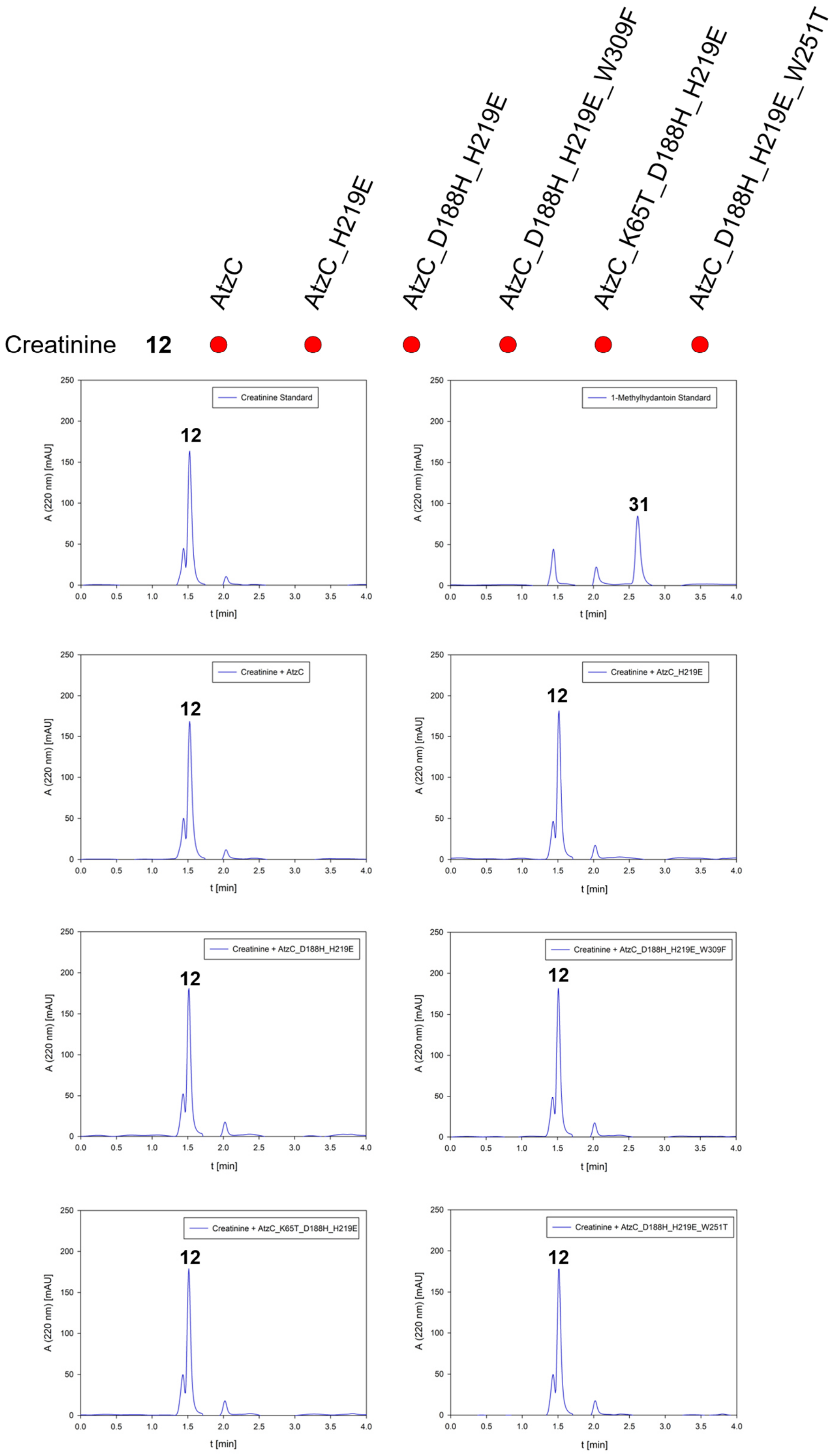

Source Data 36: HPLC-based analysis of the turnover of creatinine 12 by AtzC wildtype and variants. Substance standards as well as reaction mixtures of 500 µM **12**, 50 mM KP pH 7.5, and 2 µM of the tested enzyme were incubated at 25 °C and 500 rpm for 24 h. After centrifugation with a filter tube, the reaction products were analyzed via reversed-phase HPLC. Shown are the respective HPLC chromatograms at 220 nm. All substances were unambiguously identified using the retention times as well as the spectra of each peak. Red circles indicate enzyme-substrate pairs for which no product formation was detected.

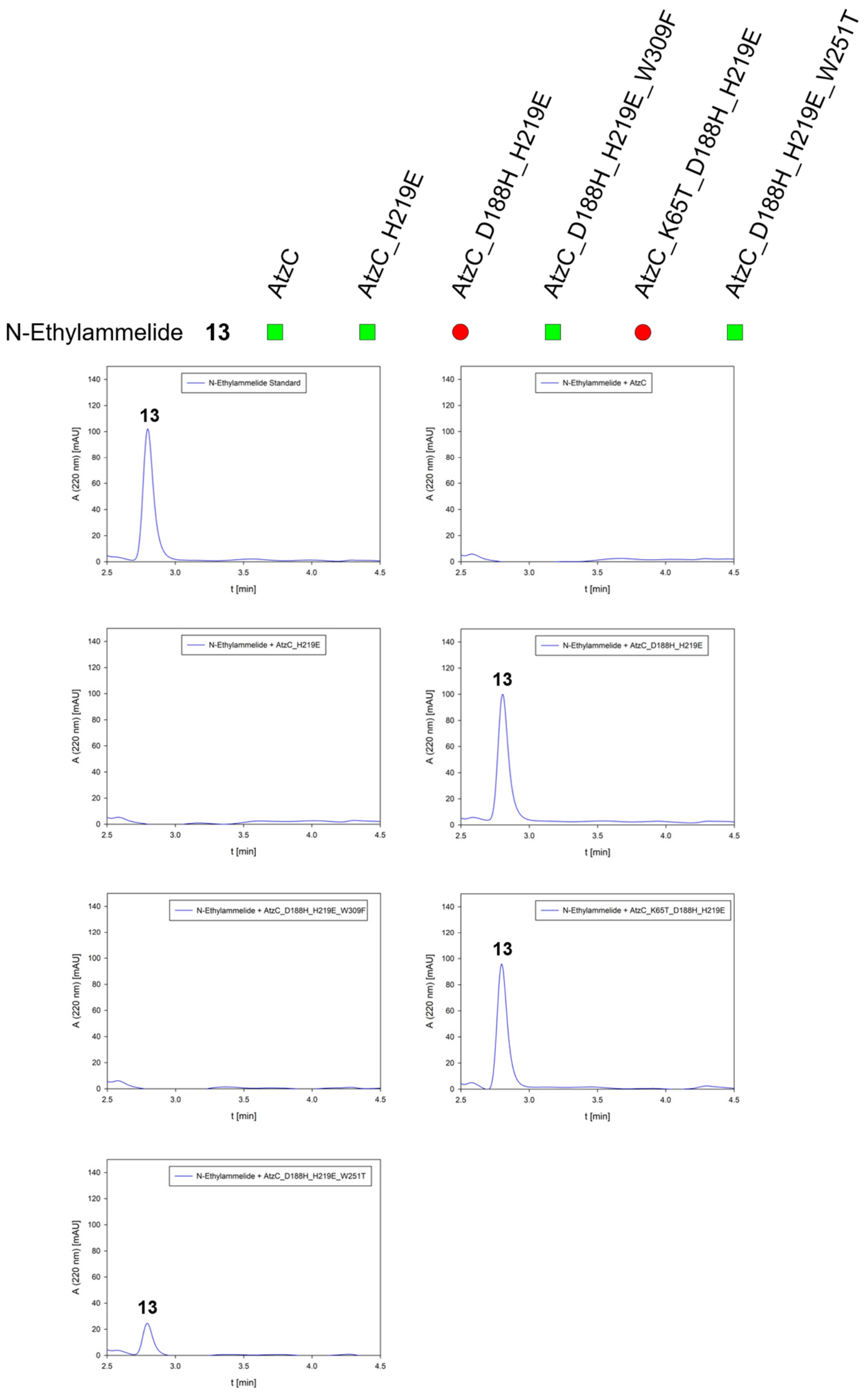

Source Data 37: HPLC-based analysis of the turnover of N-ethylammelide 13 to cyanuric acid 32 by AtzC wildtype and variants. Substance standards as well as reaction mixtures of 500 µM **13**, 50 mM KP pH 7.5, and 2 µM of the tested enzyme were incubated at 25 °C and 500 rpm for 24 h. After centrifugation with a filter tube, the reaction products were analyzed via reversed-phase HPLC. Shown are the respective HPLC chromatograms at 220 nm. All substances were unambiguously identified using the retention times as well as the spectra of each peak. As **32** was neither detectable in its substance standard (not shown) nor in the reaction mixtures, the progress of the reaction was monitored by the decrease of the peak for **13**. Light green squares indicate enzyme-substrate pairs for which substrate depletion was detected. Red circles indicate enzyme-substrate pairs for which no substrate depletion was detected.

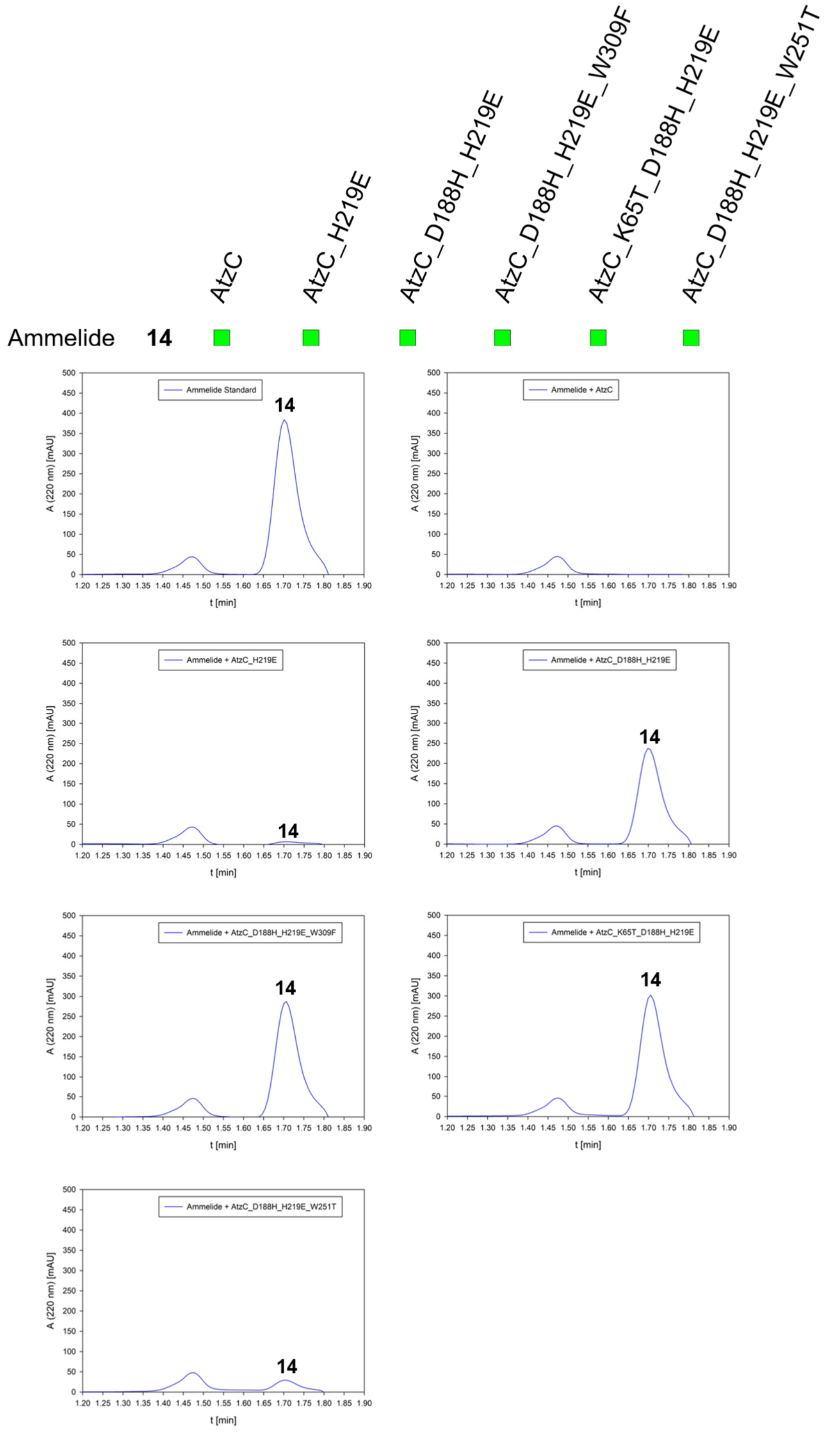

Source Data 38: HPLC-based analysis of the turnover of ammelide 14 to cyanuric acid 32 by AtzC wildtype and variants. Substance standards as well as reaction mixtures of 500 µM **14**, 50 mM KP pH 7.5, and 2 µM of the tested enzyme were incubated at 25 °C and 500 rpm for 24 h. After centrifugation with a filter tube, the reaction products were analyzed via reversed-phase HPLC. Shown are the respective HPLC chromatograms at 220 nm. All substances were unambiguously identified using the retention times as well as the spectra of each peak. As **32** was neither detectable in its substance standard (not shown) nor in the reaction mixtures, the progress of the reaction was monitored by the decrease of the peak for **14**. Light green squares indicate enzyme-substrate pairs for which substrate depletion was detected.

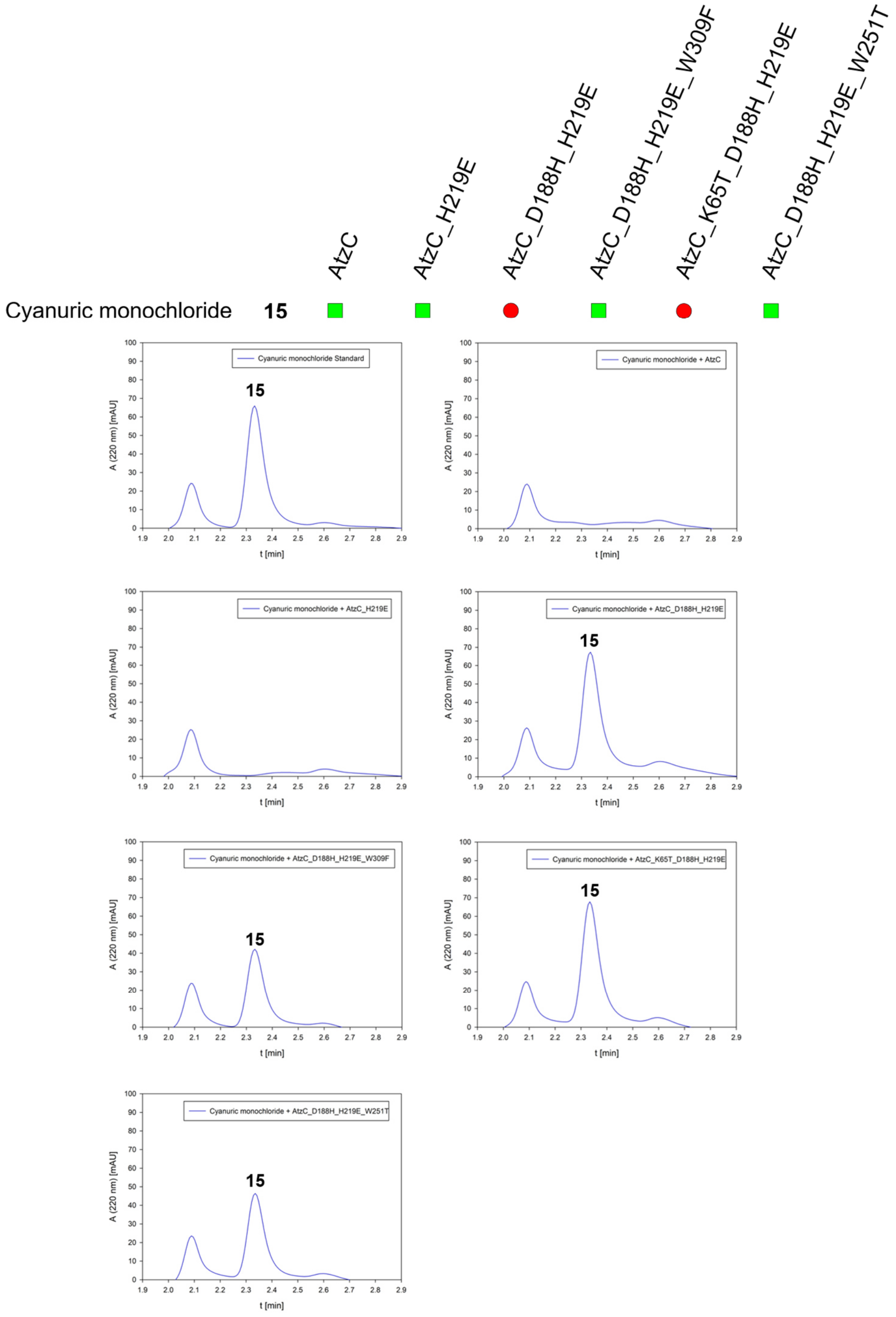

Source Data 39: HPLC-based analysis of the turnover of cyanuric monochloride 15 to cyanuric acid 32 by AtzC wildtype and variants. Substance standards as well as reaction mixtures of 500 µM **15**, 50 mM KP pH 7.5, and 2 µM of the tested enzyme were incubated at 25 °C and 500 rpm for 24 h. After centrifugation with a filter tube, the reaction products were analyzed via reversed-phase HPLC. Shown are the respective HPLC chromatograms at 220 nm. All substances were unambiguously identified using the retention times as well as the spectra of each peak. As **32** was neither detectable in its substance standard (not shown) nor in the reaction mixtures, the progress of the reaction was monitored by the decrease of the peak for **15**. Light green squares indicate enzyme-substrate pairs for which substrate depletion was detected. Red circles indicate enzyme-substrate pairs for which no substrate depletion was detected.

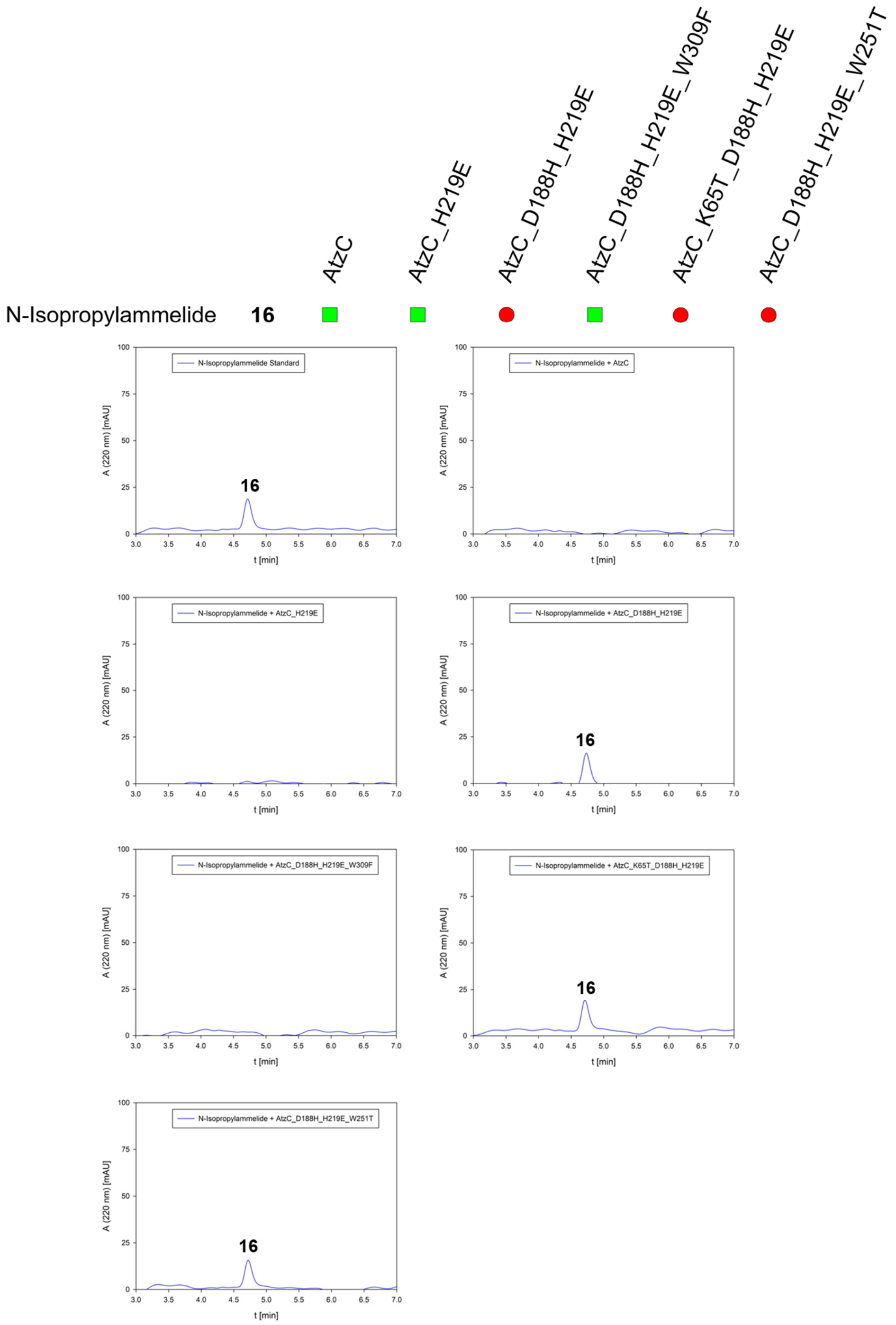

Source Data 40: HPLC-based analysis of the turnover of N-isopropylammelide 16 to cyanuric acid 32 by AtzC wildtype and variants. Substance standards as well as reaction mixtures of 500 µM **16**, 50 mM KP pH 7.5, and 2 µM of the tested enzyme were incubated at 25 °C and 500 rpm for 24 h. After centrifugation with a filter tube, the reaction products were analyzed via reversed-phase HPLC. Shown are the respective HPLC chromatograms at 220 nm. All substances were unambiguously identified using the retention times as well as the spectra of each peak. As **32** was neither detectable in its substance standard (not shown) nor in the reaction mixtures, the progress of the reaction was monitored by the decrease of the peak for **16**. Light green squares indicate enzyme-substrate pairs for which substrate depletion was detected. Red circles indicate enzyme-substrate pairs for which no substrate depletion was detected.

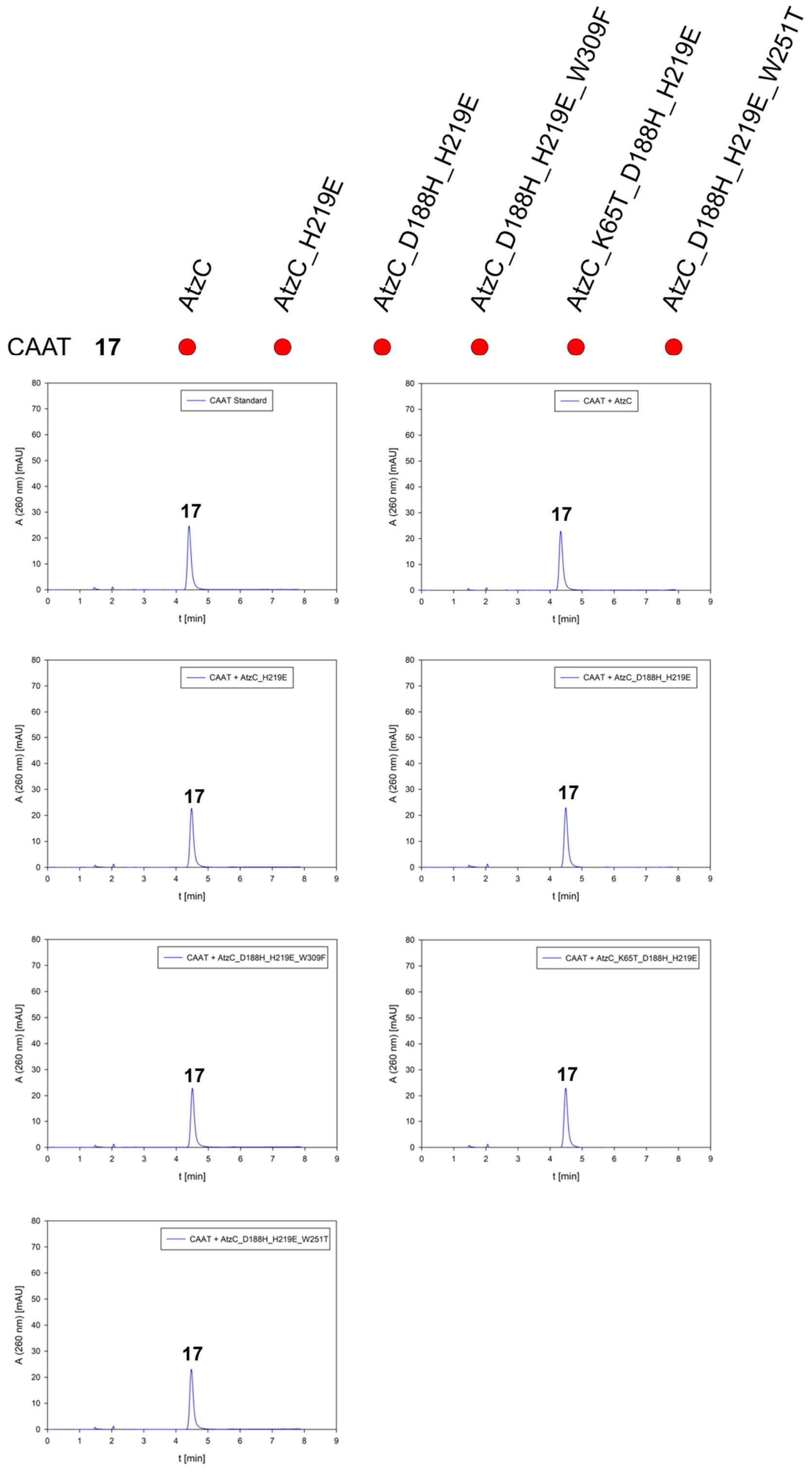

Source Data 41: HPLC-based analysis of the turnover of CAAT (2-chloro-4,6-diamino-1,3,5-triazine) 17 by AtzC wildtype and variants. Substance standards as well as reaction mixtures of 500 µM **17**, 50 mM KP pH 7.5, and 2 µM of the tested enzyme were incubated at 25 °C and 500 rpm for 24 h. After centrifugation with a filter tube, the reaction products were analyzed via reversed-phase HPLC. Shown are the respective HPLC chromatograms at 260 nm. All substances were unambiguously identified using the retention times as well as the spectra of each peak. Red circles indicate enzyme-substrate pairs for which no product formation was detected.

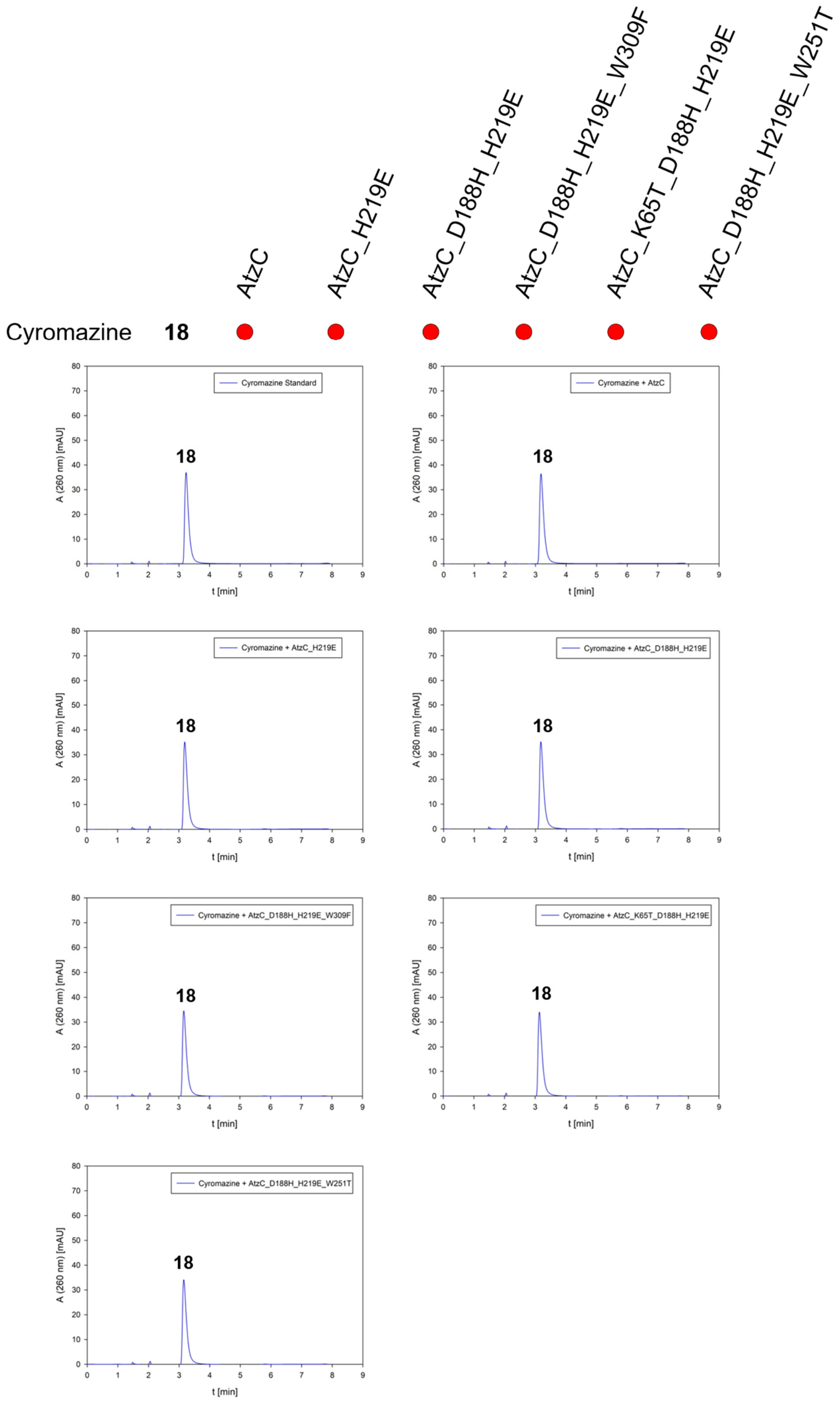

Source Data 42: HPLC-based analysis of the turnover of cyromazine 18 by AtzC wildtype and variants. Substance standards as well as reaction mixtures of 500 µM **18**, 50 mM KP pH 7.5, and 2 µM of the tested enzyme were incubated at 25 °C and 500 rpm for 24 h. After centrifugation with a filter tube, the reaction products were analyzed via reversed-phase HPLC. Shown are the respective HPLC chromatograms at 260 nm. All substances were unambiguously identified using the retention times as well as the spectra of each peak. Red circles indicate enzyme-substrate pairs for which no product formation was detected.

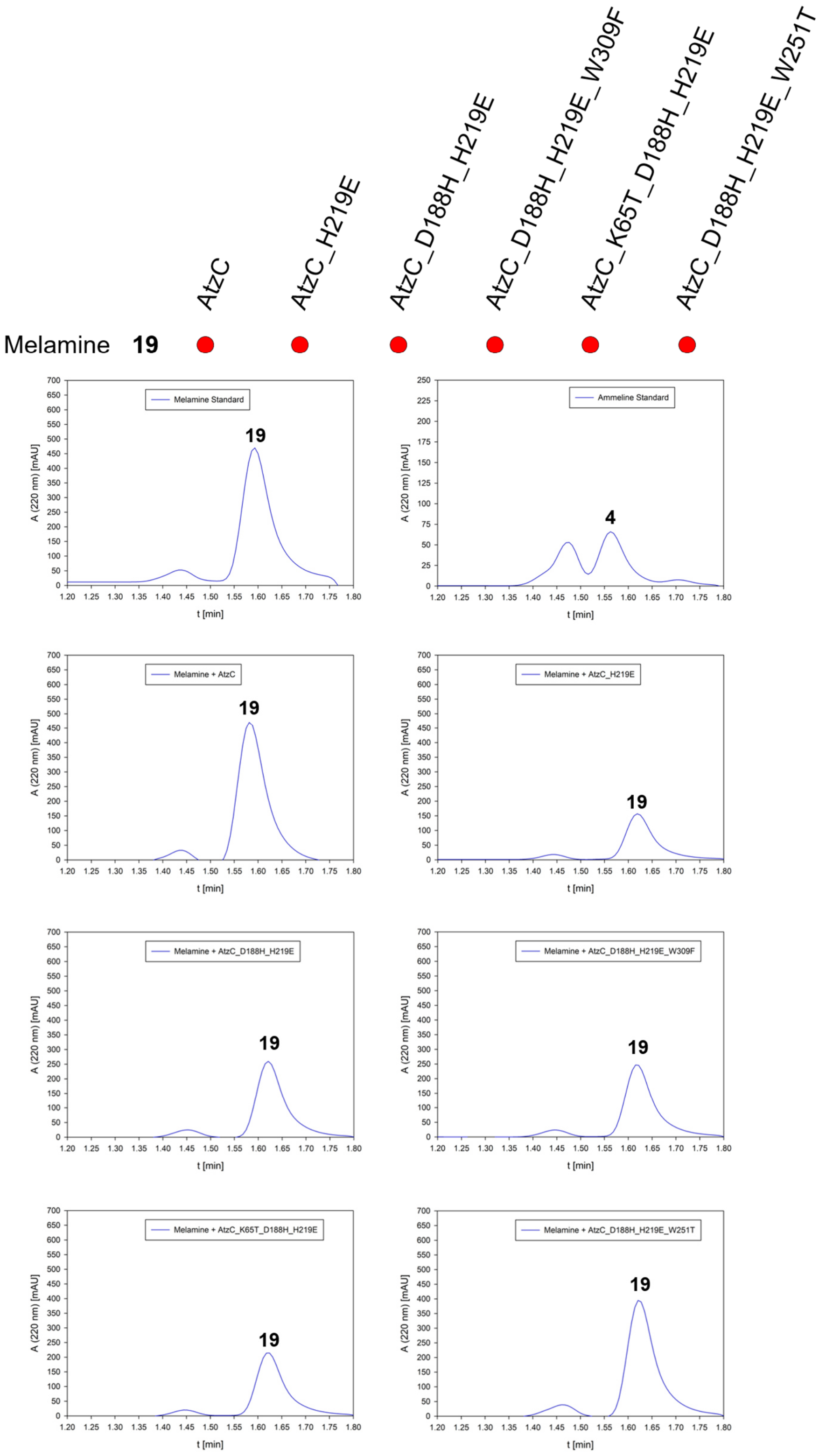

Source Data 43: HPLC-based analysis of the turnover of melamine 19 by AtzC wildtype and variants. Substance standards as well as reaction mixtures of 500 µM **19**, 50 mM KP pH 7.5, and 2 µM of the tested enzyme were incubated at 25 °C and 500 rpm for 24 h. After centrifugation with a filter tube, the reaction products were analyzed via reversed-phase HPLC. Shown are the respective HPLC chromatograms at 220 nm. All substances were unambiguously identified using the retention times as well as the spectra of each peak. Red circles indicate enzyme-substrate pairs for which no product formation was detected.

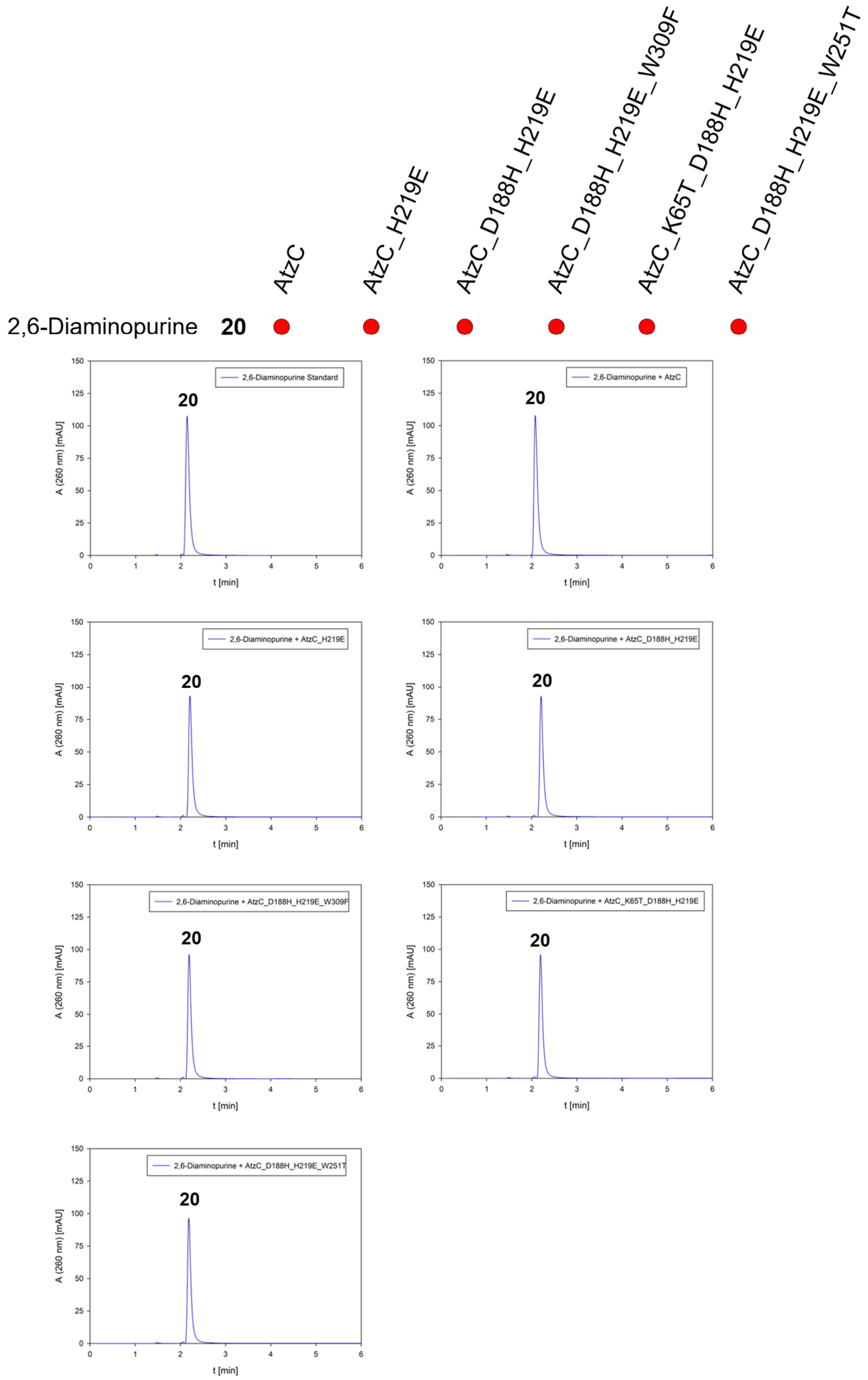

Source Data 44: HPLC-based analysis of the turnover of 2,6-diaminopurine 20 by AtzC wildtype and variants. Substance standards as well as reaction mixtures of 500 µM **20**, 50 mM KP pH 7.5, and 2 µM of the tested enzyme were incubated at 25 °C and 500 rpm for 24 h. After centrifugation with a filter tube, the reaction products were analyzed via reversed-phase HPLC. Shown are the respective HPLC chromatograms at 260 nm. All substances were unambiguously identified using the retention times as well as the spectra of each peak. Red circles indicate enzyme-substrate pairs for which no product formation was detected.

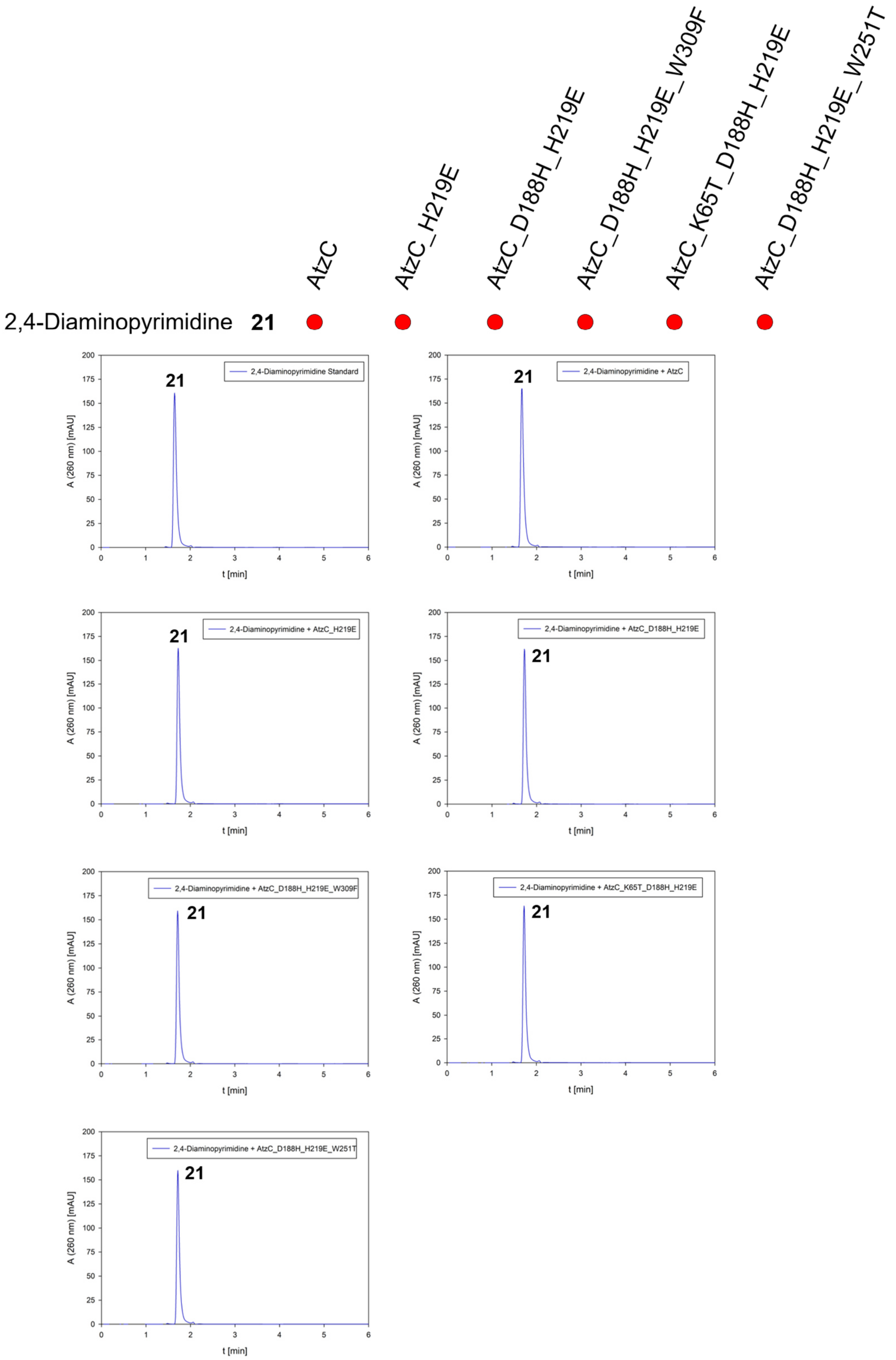

Source Data 45: HPLC-based analysis of the turnover of 2,4-diaminopyrimidine 21 by AtzC wildtype and variants. Substance standards as well as reaction mixtures of 500 µM **21**, 50 mM KP pH 7.5, and 2 µM of the tested enzyme were incubated at 25 °C and 500 rpm for 24 h. After centrifugation with a filter tube, the reaction products were analyzed via reversed-phase HPLC. Shown are the respective HPLC chromatograms at 260 nm. All substances were unambiguously identified using the retention times as well as the spectra of each peak. Red circles indicate enzyme-substrate pairs for which no product formation was detected.

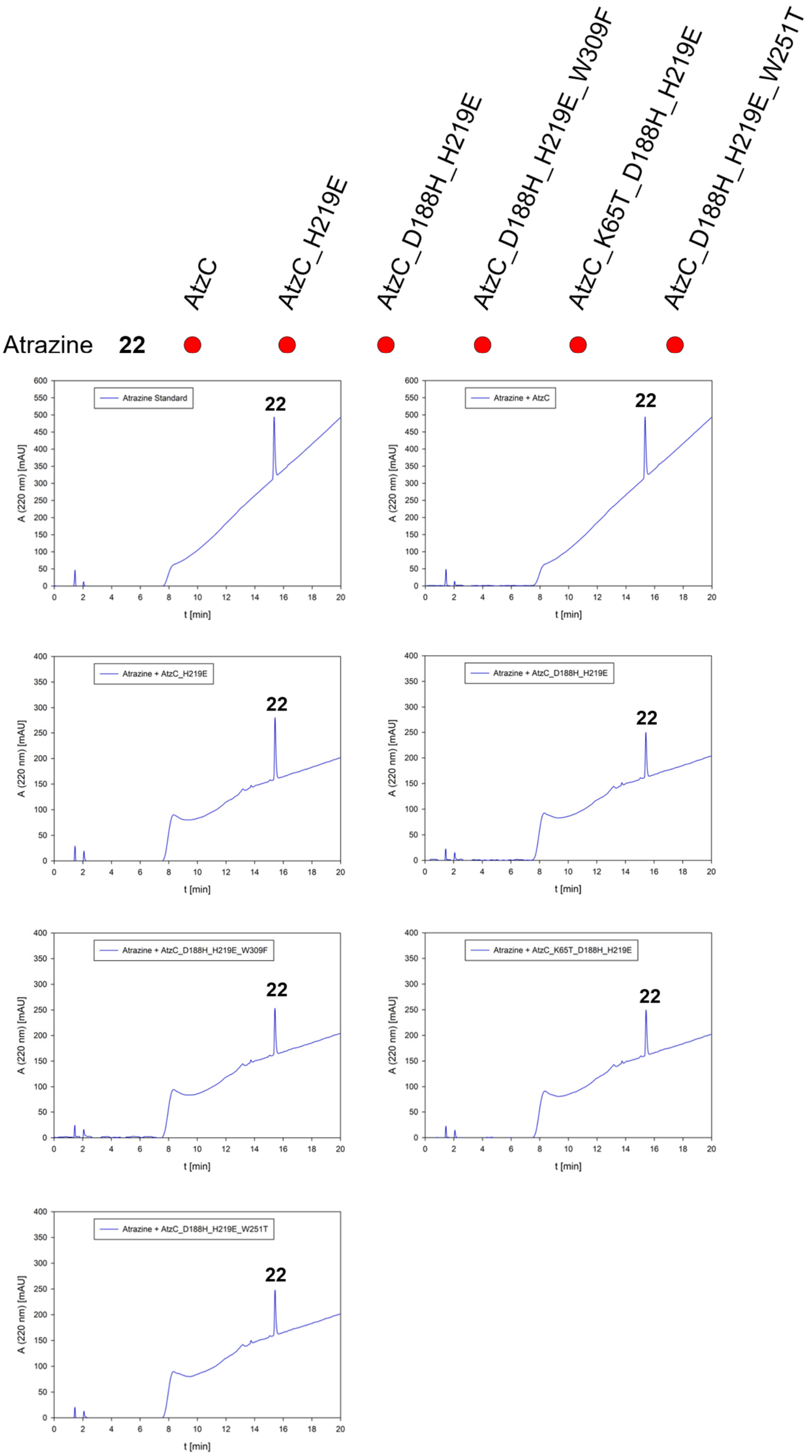

Source Data 46: HPLC-based analysis of the turnover of atrazine 22 by AtzC wildtype and variants. Substance standards as well as reaction mixtures of 500 µM **22**, 50 mM KP pH 7.5, and 2 µM of the tested enzyme were incubated at 25 °C and 500 rpm for 24 h. After centrifugation with a filter tube, the reaction products were analyzed via reversed-phase HPLC. Shown are the respective HPLC chromatograms at 220 nm. All substances were unambiguously identified using the retention times as well as the spectra of each peak. Red circles indicate enzyme-substrate pairs for which no product formation was detected.

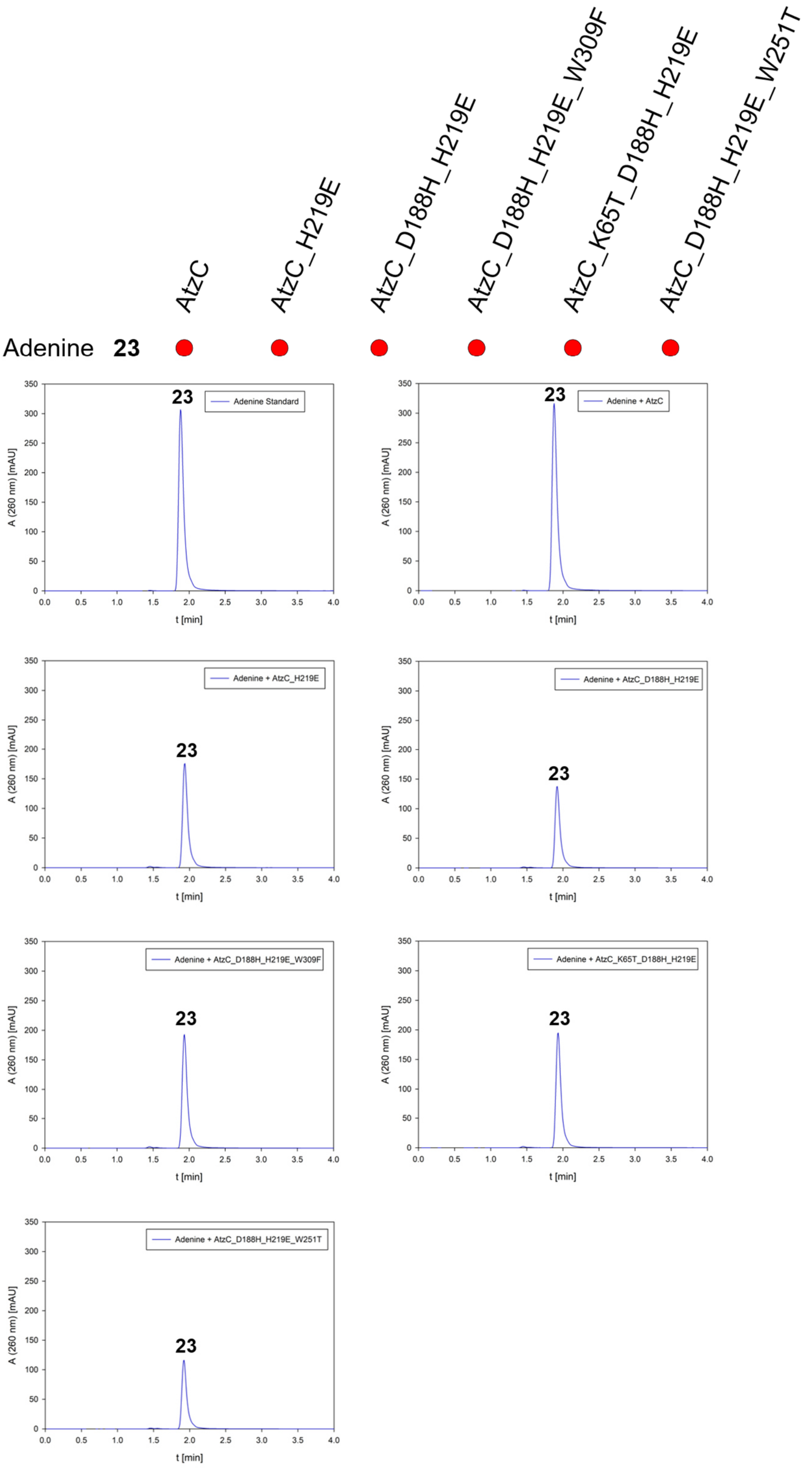

Source Data 47: HPLC-based analysis of the turnover of adenine 23 by AtzC wildtype and variants. Substance standards as well as reaction mixtures of 500 µM **23**, 50 mM KP pH 7.5, and 2 µM of the tested enzyme were incubated at 25 °C and 500 rpm for 24 h. After centrifugation with a filter tube, the reaction products were analyzed via reversed-phase HPLC. Shown are the respective HPLC chromatograms at 260 nm. All substances were unambiguously identified using the retention times as well as the spectra of each peak. Red circles indicate enzyme-substrate pairs for which no product formation was detected.

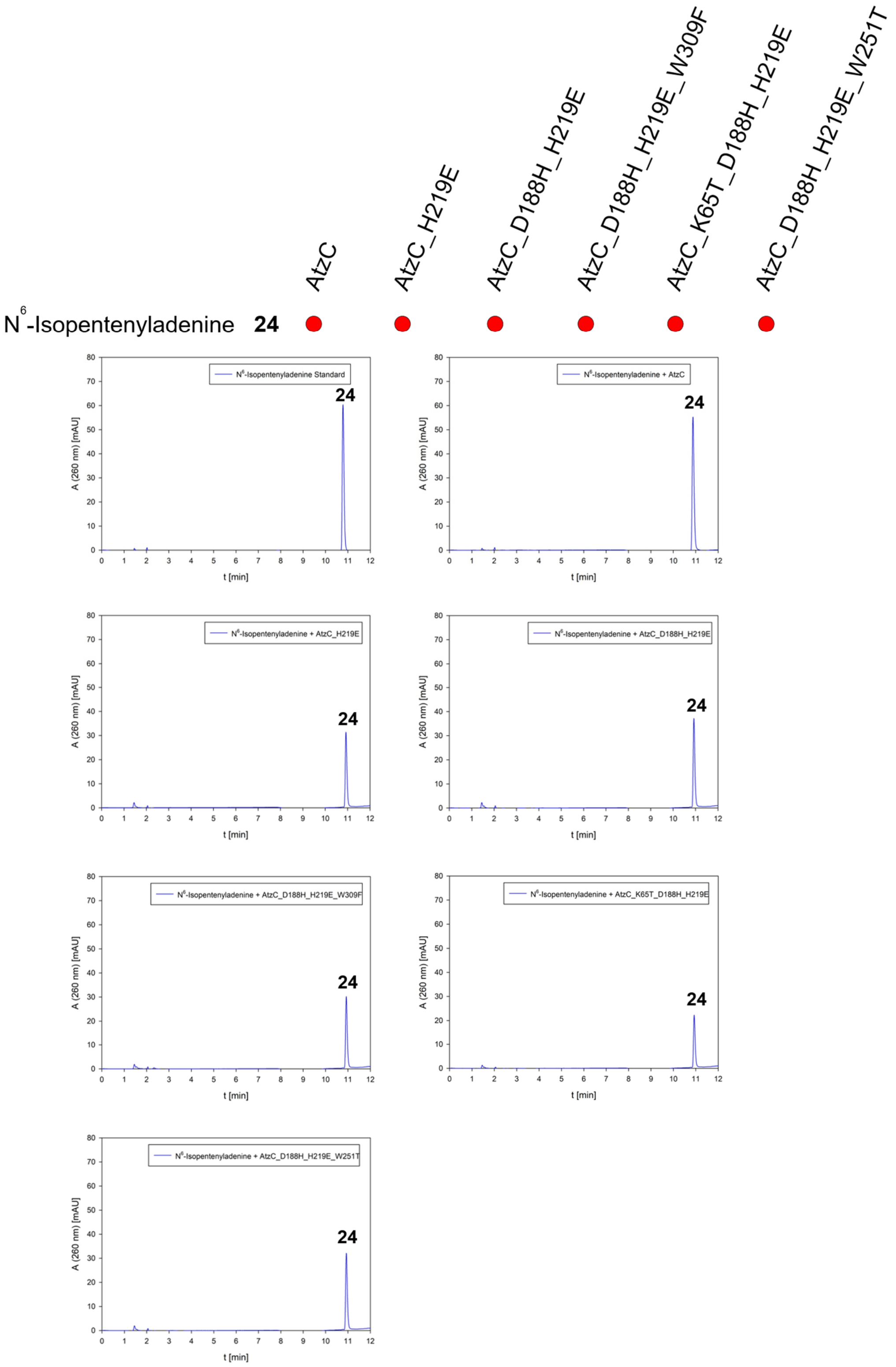

Source Data 48: HPLC-based analysis of the turnover of N⁶-isopentenyladenine 24 by AtzC wildtype and variants. Substance standards as well as reaction mixtures of 500 µM **24**, 50 mM KP pH 7.5, and 2 µM of the tested enzyme were incubated at 25 °C and 500 rpm for 24 h. After centrifugation with a filter tube, the reaction products were analyzed via reversed-phase HPLC. Shown are the respective HPLC chromatograms at 260 nm. All substances were unambiguously identified using the retention times as well as the spectra of each peak. Red circles indicate enzyme-substrate pairs for which no product formation was detected.

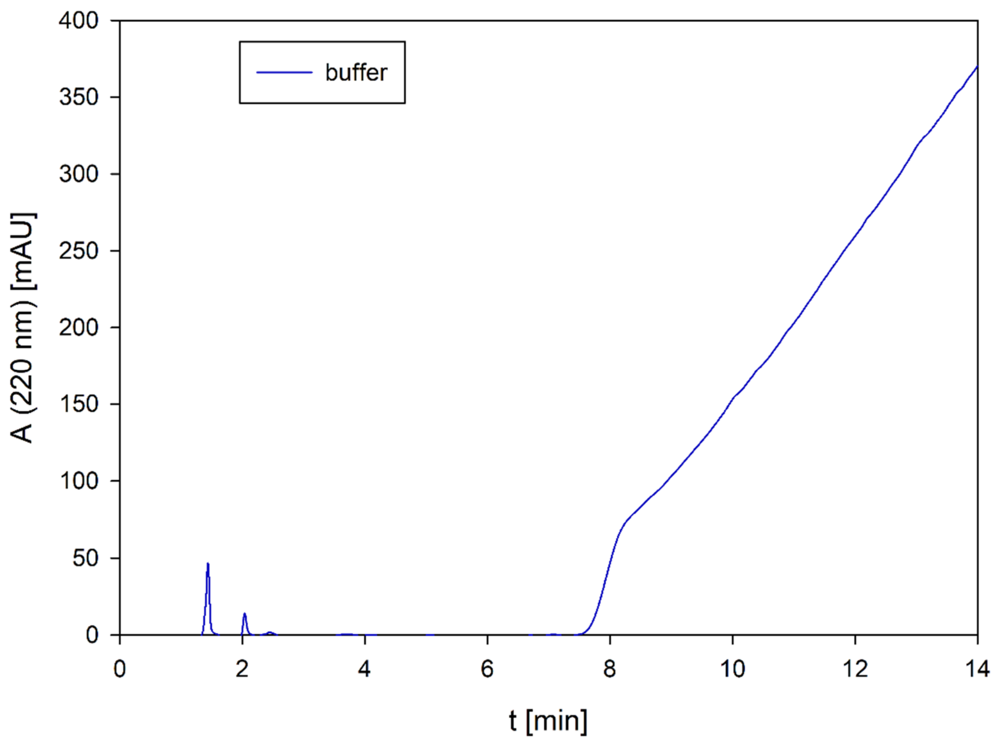

Source Data 49: HPLC-based analysis of a buffer control at 220 nm. 50 mM KP (pH 7.5) were incubated at 25 °C and 500 rpm for 24 h. After centrifugation with a filter tube, the sample was analyzed via reversed-phase HPLC. Although all substance peaks as shown in Source Data 1 - 48 were unambiguously determined using the retention times as well as the spectra of each peak, additional peaks at 220 nm were detected. However, these peaks also appear in this buffer control. Moreover, the increase in absorbance from 7.5 min onward is caused by the starting gradient elution.

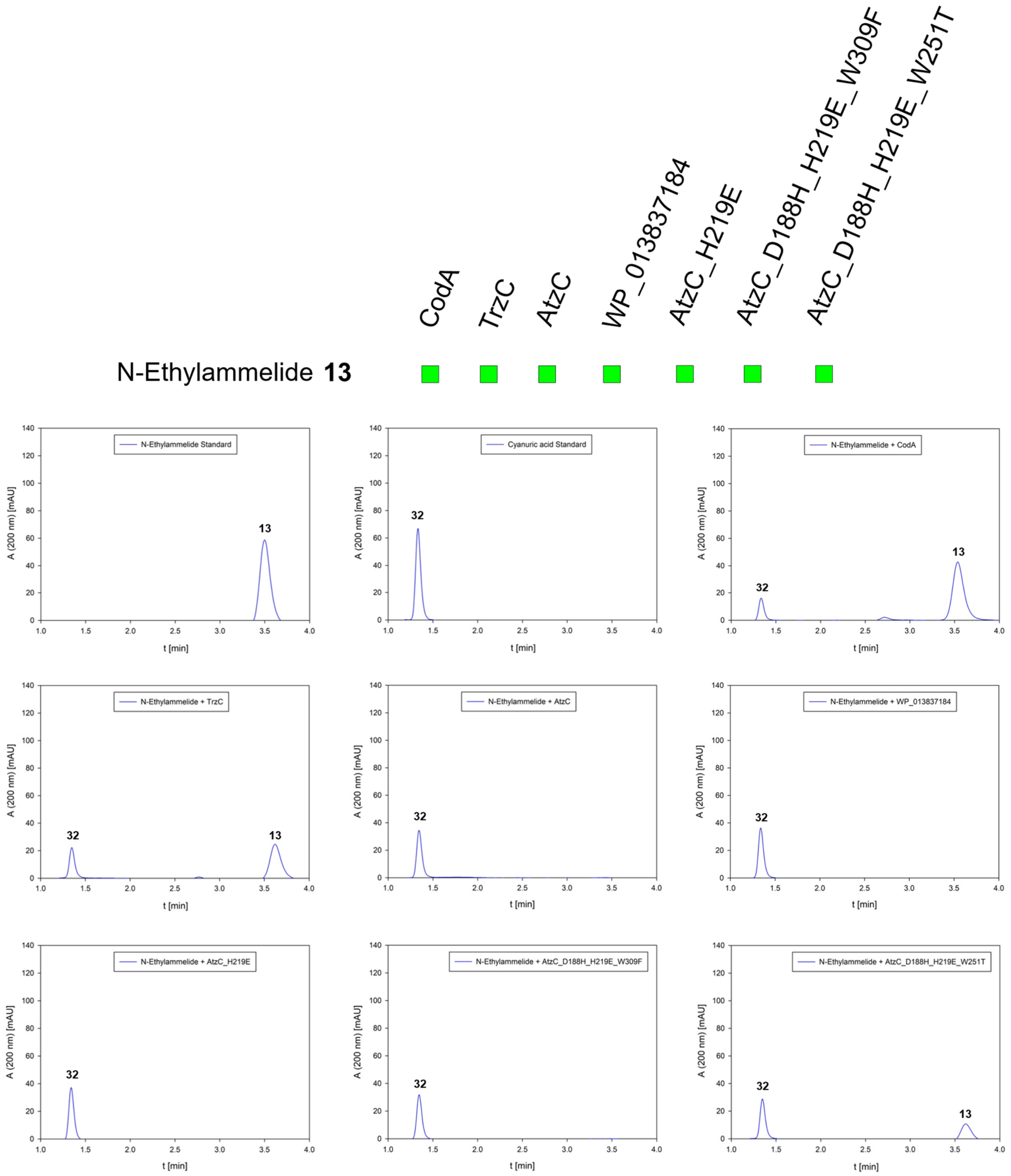

Source Data 50: HPLC-based verification of cyanuric acid 32 as product of enzymatic hydrolysis of N-ethylammelide 13. Substance standards as well as reaction mixtures of 500 µM **13**, 50 mM KP pH 7.5, and 2 µM of the tested enzyme were incubated at 25 °C and 500 rpm for 24 h. After centrifugation with a filter tube, the reaction products were analyzed by HPLC using a Luna Omega 5 µm Polar C18 100Å (150x3.0) column for direct detection of compound **32**. Shown are the respective HPLC chromatograms at 200 nm. All substances were unambiguously identified using the retention times as well as the spectra of each peak. Light green squares indicate enzyme-substrate pairs for which hydrolysis of **13** was detected in previous analyses (cf. Source Data 13, Source Data 37), and for which **32** was verified as reaction product in this analysis.

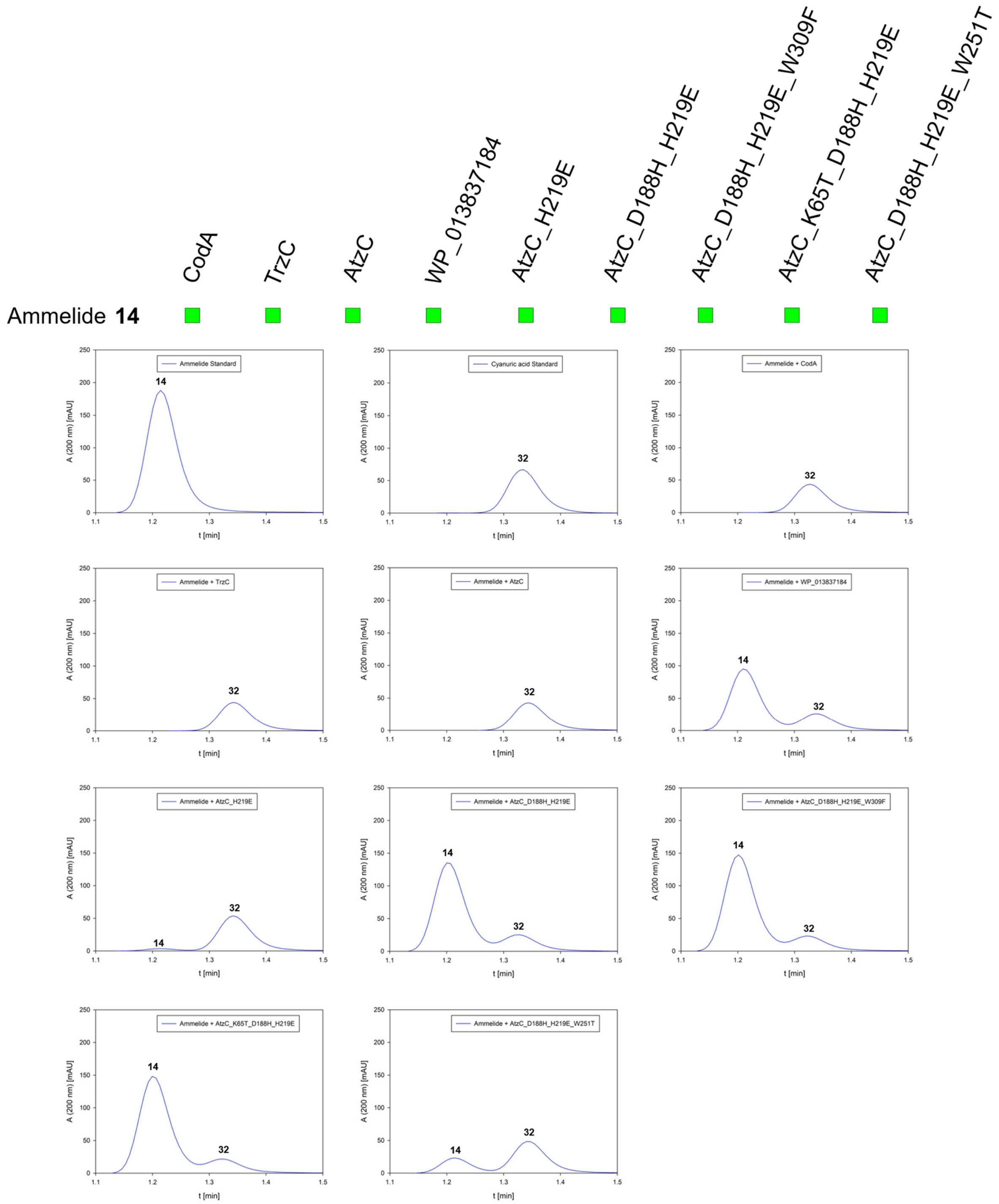

Source Data 51: HPLC-based verification of cyanuric acid 32 as product of enzymatic hydrolysis of ammelide 14. Substance standards as well as reaction mixtures of 500 µM **14**, 50 mM KP pH 7.5, and 2 µM of the tested enzyme were incubated at 25 °C and 500 rpm for 24 h. After centrifugation with a filter tube, the reaction products were analyzed by HPLC using a Luna Omega 5 µm Polar C18 100Å (150x3.0) column for direct detection of compound **32**. Shown are the respective HPLC chromatograms at 200 nm. All substances were unambiguously identified using the retention times as well as the spectra of each peak. Light green squares indicate enzyme-substrate pairs for which hydrolysis of **14** was detected in previous analyses (cf. Source Data 14, Source Data 38), and for which **32** was verified as reaction product in this analysis.

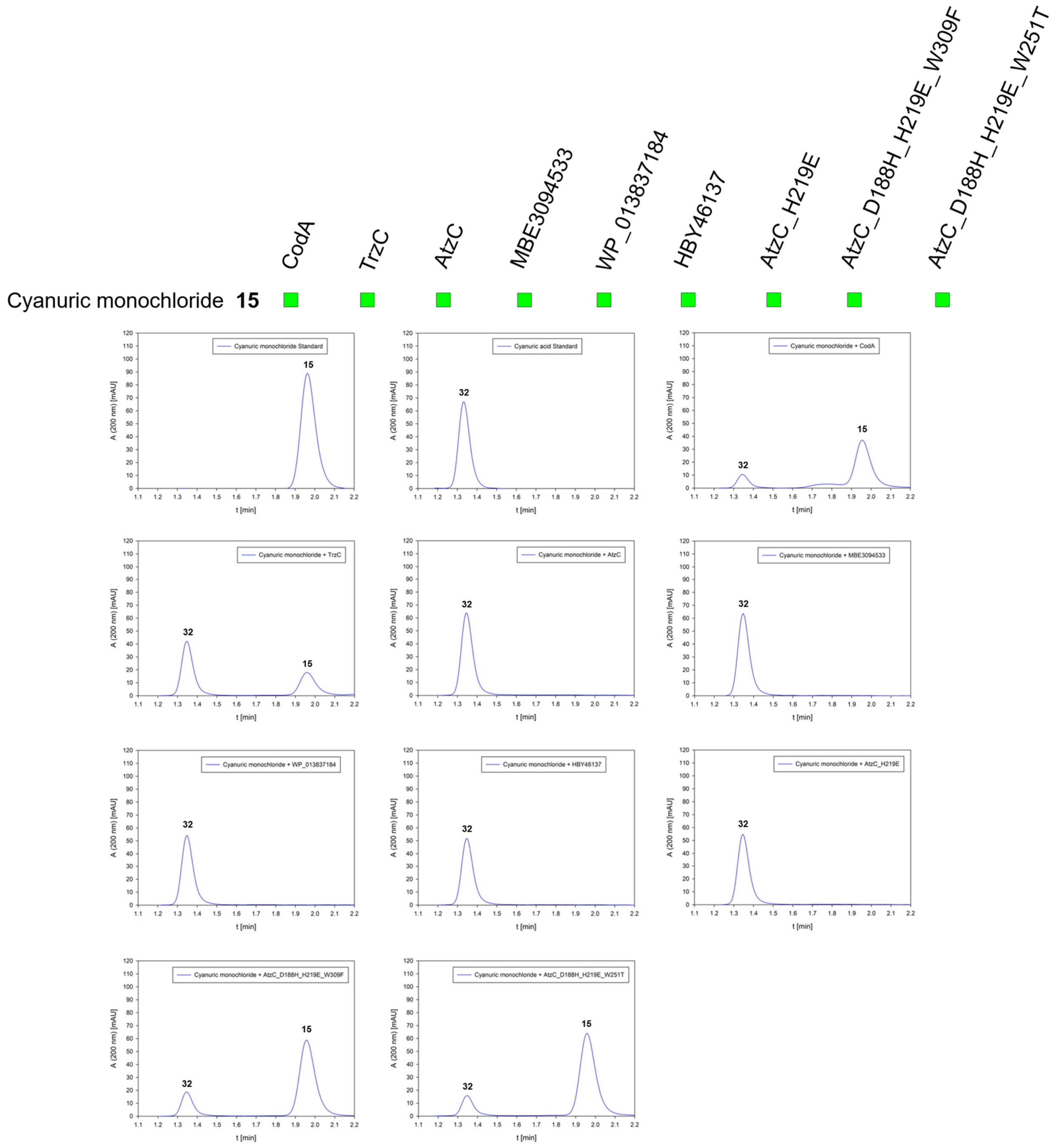

Source Data 52: HPLC-based verification of cyanuric acid 32 as product of enzymatic hydrolysis of cyanuric monochloride 15. Substance standards as well as reaction mixtures of 500 µM **15**, 50 mM KP pH 7.5, and 2 µM of the tested enzyme were incubated at 25 °C and 500 rpm for 24 h. After centrifugation with a filter tube, the reaction products were analyzed by HPLC using a Luna Omega 5 µm Polar C18 100Å (150x3.0) column for direct detection of compound **32**. Shown are the respective HPLC chromatograms at 200 nm. All substances were unambiguously identified using the retention times as well as the spectra of each peak. Light green squares indicate enzyme-substrate pairs for which hydrolysis of **15** was detected in previous analyses (cf. Source Data 15, Source Data 39), and for which **32** was verified as reaction product in this analysis.

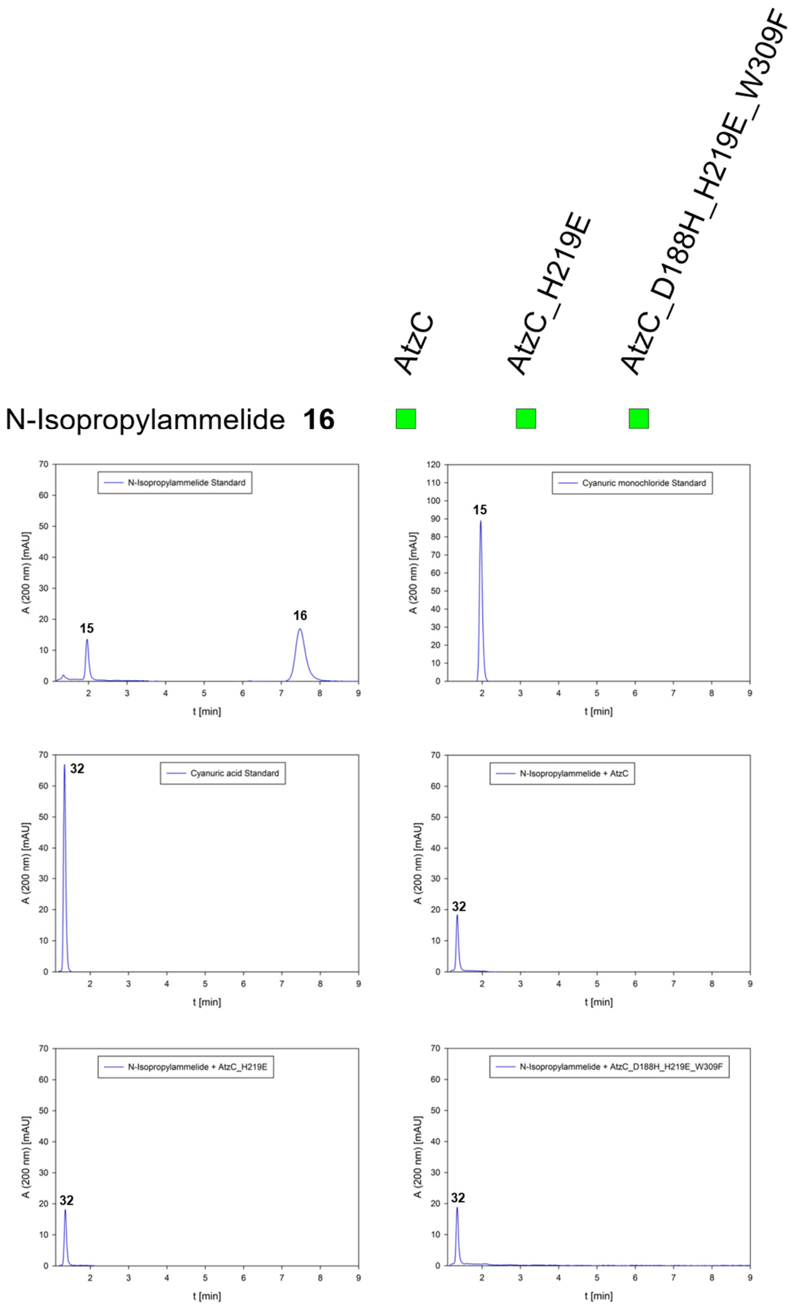

Source Data 53: HPLC-based verification of cyanuric acid 32 as product of enzymatic hydrolysis of N-isopropylammelide 16. Substance standards as well as reaction mixtures of 500 µM **16**, 50 mM KP pH 7.5, and 2 µM of the tested enzyme were incubated at 25 °C and 500 rpm for 24 h. After centrifugation with a filter tube, the reaction products were analyzed by HPLC using a Luna Omega 5 µm Polar C18 100Å (150x3.0) column for direct detection of compound **32**. Shown are the respective HPLC chromatograms at 200 nm. All substances were unambiguously identified using the retention times as well as the spectra of each peak. The standard of **16** shows residues of **15** stemming from the synthesis process. Light green squares indicate enzyme-substrate pairs for which hydrolysis of **16** was detected in previous analyses (cf. Source Data 16, Source Data 40), and for which **32** was verified as reaction product in this analysis.

References

[1] M. R. Busch, L. Drexler, D. R. Mahato, C. Hiefinger, S. Osuna, R. Sterner. Retracing the Rapid Evolution of an Herbicide-Degrading Enzyme by Protein Engineering, *ACS Catal.* **2023**, *13*, 15558–15571.

[2] S. F. Altschul, W. Gish, W. Miller, E. W. Myers, D. J. Lipman. Basic local alignment search tool, *J. Mol. Biol.* **1990**, *215*, 403–410.

[3] N. Oberg, R. Zallot, J. A. Gerlt. EFI-EST, EFI-GNT, and EFI-CGFP: Enzyme Function Initiative (EFI) Web Resource for Genomic Enzymology Tools, *J. Mol. Biol.* **2023**, *435*, 168018.

[4] K. Katoh, D. M. Standley. MAFFT multiple sequence alignment software version 7: improvements in performance and usability, *Mol. Biol. Evol.* **2013**, *30*, 772–780.

[5] G. E. Crooks, G. Hon, J.-M. Chandonia, S. E. Brenner. WebLogo: a sequence logo generator, *Genome Res.* **2004**, *14*, 1188–1190.

[6] J. Jumper, R. Evans, A. Pritzel, T. Green, M. Figurnov, O. Ronneberger, K. Tunyasuvunakool, R. Bates, A. Žídek, A. Potapenko, A. Bridgland, C. Meyer, S. A. A. Kohl, A. J. Ballard, A. Cowie, B. Romera-Paredes, S. Nikolov, R. Jain, J. Adler, T. Back, S. Petersen, D. Reiman, E. Clancy, M. Zielinski, M. Steinegger, M. Pacholska, T. Berghammer, S. Bodenstein, D. Silver, O. Vinyals, A. W. Senior, K. Kavukcuoglu, P. Kohli, D. Hassabis. Highly accurate protein structure prediction with AlphaFold, *Nature* **2021**, *596*, 583–589.

[7] J. Boitreaud, J. Dent, M. McPartlon, J. Meier, V. Reis, A. Rogozhnikov, K. Wu. Chai-1: Decoding the molecular interactions of life, *bioRxiv* **2024**, 2024.10.10.615955.

[8] CHEOPS MAXIMOBY/MOBY, version 2024.

[9] J. E. Nielsen, G. Vriend. Optimizing the hydrogen-bond network in Poisson-Boltzmann equation-based pK(a) calculations, *Proteins* **2001**, *43*, 403–412.

[10] Di Qiu, P. S. Shenkin, F. P. Hollinger, W. C. Still. The GB/SA Continuum Model for Solvation. A Fast Analytical Method for the Calculation of Approximate Born Radii, *J. Phys. Chem. A.* **1997**, *101*, 3005–3014.

[11] S. J. Weiner, P. A. Kollman, D. A. Case, U. C. Singh, C. Ghio, G. Alagona, S. Profeta, P. Weiner. A new force field for molecular mechanical simulation of nucleic acids and proteins, *J. Am. Chem. Soc.* **1984**, *106*, 765–784.

[12] M. Scheurer, P. Rodenkirch, M. Siggel, R. C. Bernardi, K. Schulten, E. Tajkhorshid, T. Rudack. PyContact: Rapid, Customizable, and Visual Analysis of Noncovalent Interactions in MD Simulations, *Biophys. J.* **2018**, *114*, 577–583.

[13] W. Humphrey, A. Dalke, K. Schulten. VMD: visual molecular dynamics, *J. Mol. Graph.* **1996**, *14*, 33-8, 27-8.

[14] B. Rohweder, F. Semmelmann, C. Endres, R. Sterner. Standardized cloning vectors for protein production and generation of large gene libraries in Escherichia coli, *BioTechniques* **2018**, *64*, 24–26.

[15] M. R. Wilkins, E. Gasteiger, A. Bairoch, J. C. Sanchez, K. L. Williams, R. D. Appel, D. F. Hochstrasser. Protein identification and analysis tools in the ExPASy server, *Methods Mol. Biol.* **1999**, *112*, 531–552.

[16] G. Estiu, K. M. Merz. The hydrolysis of urea and the proficiency of urease, *J. Am. Chem. Soc.* **2004**, *126*, 6932–6944.

[17] S. D. Aubert, Y. Li, F. M. Raushel. Mechanism for the hydrolysis of organophosphates by the bacterial phosphotriesterase, *Biochemistry* **2004**, *43*, 5707–5715.

[18] R. Shek, T. Hilaire, J. Sim, J. B. French. Structural Determinants for Substrate Selectivity in Guanine Deaminase Enzymes of the Amidohydrolase Superfamily, *Biochemistry* **2019**, *58*, 3280–3292.

[19] R. S. Hall, A. A. Fedorov, C. Xu, E. V. Fedorov, S. C. Almo, F. M. Raushel. Three-dimensional structure and catalytic mechanism of cytosine deaminase, *Biochemistry* **2011**, *50*, 5077–5085.

[20] S. S. Kamat, A. Bagaria, D. Kumaran, G. P. Holmes-Hampton, H. Fan, A. Sali, J. M. Sauder, S. K. Burley, P. A. Lindahl, S. Swaminathan, F. M. Raushel. Catalytic mechanism and three-dimensional structure of adenine deaminase, *Biochemistry* **2011**, *50*, 1917–1927.

[21] C. M. Seibert, F. M. Raushel. Structural and catalytic diversity within the amidohydrolase superfamily, *Biochemistry* **2005**, *44*, 6383–6391.

[22] M. J. Sadowsky, Z. Tong, M. L. de Souza, L. P. Wackett. AtzC is a new member of the amidohydrolase protein superfamily and is homologous to other atrazine-metabolizing enzymes., *J. Bacteriol.* **1998**, *180*, 152–158.

[23] S. Balotra, A. C. Warden, J. Newman, L. J. Briggs, C. Scott, T. S. Peat. X-ray structure and mutagenesis studies of the N-isopropylammelide isopropylaminohydrolase AtzC., *Plos One* **2015**, *10*, e0137700.

[24] J. R. Štoček, M. Dračínský. Tautomerism of Guanine Analogues, *Biomolecules* **2020**, *10(2)*, 170.

[25] J. L. Seffernick, A. G. Dodge, M. J. Sadowsky, J. A. Bumpus, L. P. Wackett. Bacterial ammeline metabolism via guanine deaminase., *J. Bacteriol.* **2010**, *192*, 1106–1112.
